# Mechanism of Astragaloside-Brucea javanica oil nanoemulsion against oral squamous cell carcinoma through CDK1/MTFR2: Network pharmacology, bioinformatics, and experimental studies

**DOI:** 10.1371/journal.pone.0329622

**Published:** 2025-08-01

**Authors:** Yihan Lai, Runqiang Liu, Huajie Shao, Juan Zhan, Yujie Ma, Lanfei Zhou, Zhichao Wan, Sicheng Li, Wei Wang, Lin Jiang, Yisen Shao

**Affiliations:** 1 Jiangxi University of Chinese Medicine, Nanchang, Jiangxi Province, China; 2 School of Stomatology, Nanchang University, Nanchang, Jiangxi Province, China; 3 Department of Oral and Maxillofacial Surgery, Key Laboratory of Oral Diseases of Traditional Chinese Medicine, Affiliated Hospital of Jiangxi University of Chinese Medicine, Nanchang, Jiangxi Province, China; 4 Department of Otorhinolaryngology, Affiliated Hospital of Jiangxi University of Chinese Medicine, Nanchang, Jiangxi Province, China; Marshall University, UNITED STATES OF AMERICA

## Abstract

**Objective:**

This study aims to investigate the core target of Astragaloside-Brucea javanica oil nanoemulsion (AS/BJO-NEs) against oral squamous cell carcinoma (OSCC) through network pharmacology, bioinformatics, and in vivo/in vitro experiments, elucidating its effects on epithelial-mesenchymal transition (EMT) mediated via MTFR2 and the underlying molecular mechanisms.

**Methods:**

By integrating network pharmacology and weighted gene co-expression network analysis, critical gene modules linked to tumor phenotypes, EMT-related adverse prognosis, and elevated MTFR2 expression were identified, pinpointing the core target gene. Gene Ontology, Kyoto Encyclopedia of Genes and Genomes, Gene Set Enrichment Analysis, molecular docking and molecular dynamics simulation were utilized for analysis and verification. OSCC cells (SCC9 and CAL27) were cultured, and stable cell lines with CDK1 knockdown or overexpression were established. The effects of AS/BJO-NEs on cell proliferation, migration, invasion, as well as the expression levels of related genes and proteins were evaluated. In vivo, OSCC xenograft models were established in nude mice. Following treatment with AS/BJO-NEs, tumor growth inhibition and changes in the expression of relevant genes were assessed.

**Results:**

CDK1 is the core target gene of AS/BJO-NEs against OSCC, and the two can bind stably. CDK1 and MTFR2 showed highly expressed in OSCC and strongly correlated with poor prognosis and EMT. In vitro experiments revealed that AS/BJO-NEs could suppress the proliferation, migration and invasion of OSCC, and down-regulate CDK1, MTFR2 and N-cadherin, whereas up-regulate E-cadherin expression. Knockdown and overexpression of CDK1 further confirmed its effects on OSCC cell phenotype, its regulatory relationship with MTFR2, and the intervention effects of AS/BJO-NEs. In vivo experiments confirmed that AS/BJO-NEs significantly inhibited tumor growth and reduced the expression of CDK1 and MTFR2 in tumor tissues.

**Conclusion:**

This study demonstrated that AS/BJO-NEs inhibited the proliferation, migration, invasion and EMT process of OSCC by down-regulating CDK1 and subsequently reducing MTFR2 expression, exerting anti-tumor effects and providing new possibilities and a theoretical foundation for OSCC treatment.

## 1. Introduction

Oral squamous cell carcinoma (OSCC) occurs in the oral mucosa and is a common malignant tumor of the head and neck region [1].Constituting roughly 90% of oral malignancies, OSCC is characterized by rapid disease progression, formidable invasion and metastatic capabilities, and dismal prognosis [2]. Although the combined use of new surgical approaches and various new drugs has enhanced the survival and life quality of patients to a certain extent, the 5-year survival rate of OSCC patients is still stays below 65% because of prominent side-effects and the problem of drug resistance [3].In recent years, a growing number of studies have shown that traditional Chinese medicine (TCM) formulas and monomers possess notable advantages in terms of multi-component, multi-pathway, and multi-target characteristics [4]. Research indicates that the traditional Chinese medicinal herbs Brucea javanica and Astragalus membranaceus exhibit antitumor activity and demonstrate significant effects in immunomodulation and anti-inflammatory responses [5,6]. The combined use of Brucea javanica and Astragalus membranaceus in the treatment of oral squamous cell carcinoma holds promising potential and merits further exploration.

Our previous studies have proved that Brucea Javanica Oil (BJO) can suppress the proliferation, invasion, and metastasis of OSCC cell lines [7]. The primary component of BJO, oleic acid, suppresses OSCC cell proliferation by inducing apoptosis and autophagy [8]. BJO, an extract from the Chinese herb Brucea javanica, is a novel, targeted, pure herbal anticancer agent [9]. It is highly effective with minimal side effects and has been widely used clinically to treat colorectal cancer [10], lung cancer [11], and gastric cancer [12]. Astragalus membranaceus, a classic herb known for benefiting Qi (the vital energy maintaining physiological balance) and consolidating the exterior, is particularly effective in tonifying the Qi of the spleen and lungs. In traditional Chinese medicine formulations, Astragalus can be combined with various other herbs to achieve diverse therapeutic effects and is widely used in cancer treatment. One of its active components, Astragaloside IV(AS-IV), inhibits the proliferation, migration, invasion, and epithelial-mesenchymal transition (EMT) of oral cancer cells by regulating the AMPK and AKT/mTOR pathways [13].

Additionally, we have identified that BJO can suppress invasion and metastasis of OSCC cells by downregulating Mitochondrial Fission Regulator 2 (MTFR2) [14].Studies have shown that MTFR2 promotes mitochondrial fission and is involved in tumorigenesis [15], playing a facilitating role in various cancers, including gastric cancer, hepatocellular carcinoma, and lung cancer [16,17].In our previous research, through searches of the TCGA and GEO databases as well as clinical sample analyses, we identified that MTFR2 is expressed at a high level in tumor tissues and linked to an unfavorable prognosis in OSCC patients. Experiments have demonstrated that upregulation of MTFR2 can promote the proliferation, migration, invasion, and EMT of OSCC cells [18]. EMT represents a biological process during which epithelial cells shed their typical morphological traits and take on the characteristics of mesenchymal cells [19]. This process is crucial in tumorigenesis, especially in OSCC, where it plays a key oncogenic role [20,21]. E-cadherin and N-cadherin are cell adhesion molecules belonging to the cadherin family and play critical roles in the EMT process. BJO can inhibit the invasion, metastasis, and EMT process of OSCC cells by down-regulating MTFR2, while AS-IV can also suppress the EMT process of OSCC cells. The potential synergy in their mechanisms of action provides important clues for the study of combination therapy targeting MTFR2.

Currently, breakthroughs have been made in the basic research of TCM nanomedicines [22], which can translate the action mechanism of TCM from macroscopic level to molecular and microscopic levels. Thereby, they can enhance the targeting of TCM and increase the utilization rate of active ingredients, effectively compensating for the deficiencies in the application of TCM [23]. Studies have also shown that BJO has promising potential as a nanocarrier, extending circulation time in the body and improving bioavailability [24]. Based on our team’s long-term clinical observations, the combination of BJO and AS-IV can enhance the therapeutic efficacy of OSCC treatment. Inspired by this finding, we prepared AS-IV (Chengdu Pufei De Biotech Co., Ltd) and BJO (Dalian Meilun Biotechnology Co., Ltd) into Astragaloside-Brucea Javanica Oil nanoemulsions (AS/BJO-NEs) by high-pressure homogenization. We have detected the physical and chemical properties of AS/BJO-NEs, including appearance, morphology, particle size, polydispersity index, zeta potential, stability, and in vitro release, confirming its good stability. The high-performance liquid chromatography (HPLC) chromatograms also demonstrated that the established liquid chromatography method exhibited good specificity. Meanwhile, the results of in vivo experiments demonstrated that AS/BJO-NEs exhibited notable anti-tumor efficacy along with a favorable safety profile [25]. However, the exact mechanisms by which AS/BJO-NEs exert anti-tumor effects on OSCC remain to be elucidated.

During the advancement of computational biology, systems biology, and pharmacology, the integration of network pharmacology and bioinformatics has shown significant advantages in tumor research and personalized treatment. Network pharmacology is a powerful method that applies network analysis and machine learning to predict disease targets and drug mechanisms from a holistic and systemic perspective [26]. Bioinformatics analysis is essential for pinpointing and assessing genes implicated in carcinogenesis and tumor progression [27]. Weighted gene co-expression network analysis (WGCNA) clusters genes into modules based on their expression correlations, facilitating gene function inference, clinical association studies, and the identification of key genes [28]. In addition, molecular docking can be employed to simulate and predict the binding mode as well as affinity between receptors and ligands. Molecular dynamics simulation (MDS) can simulate the movement of molecular systems in a system based on Newtonian mechanics and is often used to assess the steadiness and flexibility of the system and to discover the key dynamic processes by which drugs exert their effects [29].

Currently, the potential core targets and mechanisms by which AS/BJO-NEs exert anti-OSCC effects, as well as their association with MTFR2, remain unclear and warrant further investigation. Therefore, in the present study, we integrated network pharmacology and bioinformatics methods to clarify the key target of AS/BJO-NEs in mediating anti-OSCC via the MTFR2 gene. Additionally, we investigated a series of tumor processes, especially EMT, triggered by the regulation of MTFR2 by this target. Furthermore, we validated the molecular mechanisms accounting for the inhibitory effects of AS/BJO-NEs on OSCC through in vitro experiments.

## 2. Materials and methods

### 2.1. Network pharmacology and Bioinformatics

#### 2.1.1. Screening of active ingredients and targets of AS/BJO-NEs.

The main chemical constituents of “YA DAN ZI” (Brucea javanica) were screened using the Platform (TCMSP, https://tcmsp-e.com/tcmsp.php). Moreover, according to the pharmacokinetic characteristics of ADME, the following parameters were set: oral bioavailability(OB)≥30% and drug-likeness(DL)≥0.18 to screen out the main chemical components of Brucea javanica.The SMILE numbers of main chemical components of Brucea javanica and the active component AS-IV of Astragalus were acquired via Pubchem database (https://pubchem.ncbi.nlm.nih.gov/). The putative targets were initially retrieved through the SwissTargetPrediction platform (http://www.swisstargetprediction.ch/index.php). Subsequently, the UniProt (https://www.uniprot.org/) ID mapping module was employed to validate target specificity by inputting the gene symbols of identified targets, with Homo sapiens designated as the biological species and UniProtKB selected as the reference database to obtain the mapping results. The targets that could not be mapped were considered as blank targets. Subsequently, the unmappable targets were deleted and the data was organized.

#### 2.1.2. Screening of related target genes of AS/BJO-NEs against OSCC.

All OSCC disease target genes were obtained through GeneCards (https://www.genecards.org/) and DisGeNET(http://www.disgenet.org/) databases and combined with UniProt database to normalize the acquired target names. The intersection of AS/BJO-NEs action target genes and the related genes of OSCC was acquired on the jvenn platform (https://jvenn.toulouse.inrae.fr/app/example.html) [30].

#### 2.1.3. Protein-protein interaction(PPI) network.

The effective component targets of AS/BJO-NEs and the targets of OSCC that were obtained were employed to generate a visualization map of the drug-disease-target pathway using Cytoscape 3.10.1 software. Subsequently, the overlapping targets of AS/BJO-NEs and OSCC were inputted into the GeneMANIA database (https://genemania.org/) to obtain a PPI visualization network map.

#### 2.1.4. Data collection and differentially expressed gene (DEG) analysis.

The TCGA-HNSC dataset were sourced from the TCGA database (https://portal.gdc.cancer.gov/). After preprocessing, the gene expression data and clinical information were obtained. Samples from oral sites were selected, and finally the gene expression data and clinical data of TCGA-OSCC (including 32 normal control samples and 325 OSCC samples) were acquired. The TCGA-PanCancer transcriptome data was retrieved from the UCSC Xena database (https://xenabrowser.net/datapages/) to serve as the pan-cancer differential analysis data of genes. The original expression profile datasets of the OSCC group and the normal control group, namely the GSE37991 dataset (which includes 40 control samples and 40 OSCC samples), were downloaded from the GEO database (https://www.ncbi.nlm.nih.gov/geo/). Subsequently, the “Limma” package, the“DESeq2”package and the “ggplot2” package were employed to carry out differential analysis and draw volcano plots for visualizing the expression patterns of the differential genes.

#### 2.1.5. Acquisition and unsupervised clustering of EMT-related genes and modeling of EMT prognosis.

Gene sets related to EMT were collected from the GSEA database (http://www.gsea-msigdb.org/gsea/index.jsp) using “EMT” and “epithelial- mesenchymal transition” as search terms, with the species restricted to human. Univariate analysis was conducted on EMT-related genes using the transcriptome data and survival data of TCGA-OSCC samples. The obtained results were adjusted using the Benjamini-Hochberg procedure. Subsequently, multivariate Cox regression analysis incorporating clinical information was performed for each gene in the results, yielding genes with P < 0.05. Using the “ConsensusClusterPlus” R software, an unsupervised clustering analysis was conducted on the TCGA-OSCC dataset to classify TCGA-OSCC into distinct EMT subgroups. The clustering process employed the “km” clustering method, with distances calculated using the “Euclidean” method, and a sampling frequency of 1000 times. Kaplan-Meier (KM) analysis was performed using Survminer and R survival software package to compare the survival prognosis among different subgroups of the cohort.

#### 2.1.6. Weighted Gene Co-expression Network Analysis (WGCNA).

Analysis of differential genes obtained from the TCGA-OSCC transcriptome dataset using the “WGCNA” R package. We figured out the soft – threshold β, and then we set the minimum module size at 50 and the cutting height at 0.25. A correlation matrix between gene modules and phenotypes was established, with EMT clustering subtypes, sample types, and gene expression groups selected as related phenotypes. The modules significantly correlated with the phenotypes were screened out, and the genes contained in these modules were crossed with the intersection target genes of the proposed anti-OSCC for further analysis.

#### 2.1.7. Relevant analysis and visualization of core genes.

Correlation analysis and visualization were performed using the “ggPrism”, “ggExtra”, and “ggplot2” packages in R language. Meanwhile, the core genes expression in normal control group and tumor group samples was examined through the TCGA-OSCC and GSE37991 datasets. Based on the survival data from TCGA-OSCC, Kaplan-Meier analysis and univariate/multivariate Cox analysis were conducted using the “Survminer” and “ggplot2” software packages, and the results were visualized. The “pROC” package was utilized for conducting receiver operating characteristic curve (ROC) analysis, and the area under the curve (AUC) value was acquired.

#### 2.1.8. Gene Ontology(GO) and Kyoto Encyclopedia of Genes and Genomes(KEGG) Enrichment Analysis.

GO and KEGG enrichment analyses were conducted by the DAVID Functional Annotation Bioinformatics Microarray Analysis platform (https://david.ncifcrf.gov/), encompassing the three components of GO analysis. A portion of GO terms and some KEGG pathways were selected and imported into the SangerBox platform (http://www.sangerbox.com/home.html) [31] for the drawing of visualization graphs.

#### 2.1.9. Gene Set Enrichment Analysis(GSEA).

GSEA is employed to ascertain whether a pre-defined set of gene sets exhibits significant differences in enrichment among diverse biological states. Relying on transcriptome data, the pre-defined gene sets, namely h.all.v2024.1.Hs.symbols.gmt, was extracted from the GSEA database. Utilizing the “GSEA” package, GSEA analysis was conducted to obtain enrichment scores and significance of enrichment. This enables the determination of the correlation trends of genes in different biological states, assessment of the crucial pathways and biological processes that might impact diseases, and visualization of some of the outcomes.

#### 2.1.10. Molecular docking technology.

The mol2 files of the effective component ligands associated with the core genes were downloaded from the TCMSP database. The structures of the small molecule ligands were optimized by means of Chem3D software, and the optimized files were saved in the mol2 format. The core target gene receptor files originated from the Protein Data Bank (PDB) (https://www.rcsb.org/), and were processed in AutoDock4, involving the removal of water molecules, addition of charges, and parameterization. Subsequently, the components and targets with favorable binding activity were screened in accordance with affinity, and further visualized using PyMOL2.

#### 2.1.11. Molecular Dynamics Simulation (MDS).

MDS were conducted using GROMACS 2023.2. The complex was input into the Solution Builder module of CHARMM-GUI [32,33] to establish a solvent box at least 1 nm away from the complex boundary. Water molecules (TIP3), Na+(sodium ions), and Cl-(chloride ions) were added to balance the system’s charge and maintain the physiological ion concentration at 0.15 mol/mol. Set periodic boundary conditions, automatically generate PME-FFT grid information for the system, and add CHARMM36m force field parameters for the proteins and small molecules in the system to generate topology files.Energy minimization was accomplished through 5000 steps of the steepest descent method, with long-range electrostatic interactions calculated using the PME method. The energy minimization process was halted when the absolute value of the maximum force (emtol) acting on any atom in the system was less than 1000 kJ/mol·nm. After completing the energy optimization, a 125 ps pre-equilibration (NPT) was conducted under constant volume and a constant heating rate. The final MD simulation lasted for 100 ns with a step size of 2 fs. Images were generated using DuIvyTools 0.6.0 [34]. The protein-ligand binding free energy in aqueous solution was computed utilizing Molecular Mechanics Poisson-Boltzmann Surface Area (MMPBSA), and this was accomplished via gmxtools [35]. (MMPBSA Computational formula ΔGbind = ΔEMM+ΔGsolv-TdS; ΔEMM = ΔEcovalent + ΔEelectrostatic + ΔEvdW; ΔEcovalent = ΔEbond + ΔEangle + ΔEtorsion; ΔGsolv = ΔGpolar + ΔGnonpolar)

### 2.2. Experimental validation section

#### 2.2.1. Cell culture and materials.

OSCC cell lines (SCC9 and CAL27) were acquired from Wuhan Puresyn Biotechnology Co.,Ltd. and routinely cultured in T25 cell culture flasks containing DMEM/F-12 medium (G4612,Servicebio,China) or DMEM medium (G4524, Servicebio, China) supplemented with 10% fetal bovine serum (30067334, Gibico, USA), 100 U/mL penicillin and 100 μg/ml streptomycin at 37°C in a 5% CO2 incubator. Logarithmic phase cells were subsequently harvested for experimentation.

#### 2.2.2. Cell transfection and establishment of stable cell lines.

Using lentiviral vectors designed and constructed by Zolgene Biotechnology Co., Ltd for overexpression and knockdown RNA of CDK1 (SH-CDK1–1, SH-CDK1–2, SH-CDK1–3 and OE-CDK1), along with negative controls (SH-NC, OE-NC),detailed information in S1 Table. The aforementioned lentiviruses were transfected into CAL27 cells, and after transfection, the cell supernatant was collected, centrifuged, and subjected to viral titer detection. Different concentration gradients of the virus were then used to transfect the cells to determine the optimal Multiplicity of Infection (MOI). Stable oral squamous carcinoma cell lines were selected using puromycin to achieve stable overexpression and knockdown of CDK1 in the CAL27 cell line.

#### 2.2.3. Cell counting kit-8 (CCK-8) assay.

Suspensions of CAL27 and SCC9 cells were inoculated into 96-well plates at a concentration of 1 × 103 cells per well and cultured for 24 hours until cells adhered. Different concentrations (2 μg/ml, 4 μg/ml, 6 μg/ml, 8 μg/ml, 10 μg/ml) were added, with untreated cells serving as the control group. After incubating for 24 and 48 hours, 10 μl of CCK-8 solution (APExBIO Technology LLC, USA) was added. Following a 2-hour incubation in the dark, the absorbance at 450 nm was measured using a multifunctional microplate reader (EnSight-PerkinElmer,USA). The experiment was repeated 3 times. Cell viability (%)= (OD treatment-OD blank)/(OD control-OD blank) × 100%. The median inhibitory concentration (IC50) was determined by fitting growth inhibition curves to the data using GraphPad Prism 9.0.

#### 2.2.4. Colony formation assay.

In each experimental group, 1,000 cells were plated at a density of one well per 1,000 cells in 6-well plates and then incubated for 14 days. Once the cloning process was completed, the cells were fixed with 1mL of 4% paraformaldehyde per well, followed by a single wash with phosphate-buffer solution (PBS). Subsequently, each well was treated with 1mL of crystal violet staining solution for colony staining. Once dried, images were captured using a digital camera, and cell colonies were manually counted. Statistical bar graphs were subsequently generated using Prism 9.0 software.

#### 2.2.5. Wound healing assay.

12 hours subsequent to seeding, a confluent monolayer of OSSC cells was established on the bottom of a 6-well plate. Subsequently, a vertical scratch was introduced onto the cell-covered surface using a sterile 200-µL plastic pipette tip. Thereafter, the debris was eliminated through washing with PBS. The scratched monolayers were then incubated at 37°C for 24 hours. Wound closure was assessed using an inverted fluorescence microscope (Leica DMi1, Germany) at three random fields under 200x magnification. Images were analyzed using ImageJ software (version 1.53, National Institutes of Health, USA) to quantify in vitro scratch wound healing assays. The wound closure percentage was calculated as follows: Scratch width = Scratch area/ Scratch length; Wound closure percentage = [(T0 hour scratch width - T24/48 hour scratch width)/ T0 hour scratch width] × 100%.

#### 2.2.6. Transwell matrigel invasion assay.

Transwell chambers with 8.0 µm pore membranes (3428, Corning, USA) in a 48-well format were used to assess the invasion capabilities of OSCC cells. Prior to cell seeding, each transwell insert was coated with 100 µg/cm^2^ Matrigel (Corning, USA) at 37°C for 2 hours. In total, 3 × 10⁴ cells, which were suspended in 200 µL of serum – free medium, were introduced into the upper chamber. Concurrently, 600 µL of complete medium was added to the lower chamber to act as a chemoattractant. Following a 24-hour incubation period at 37°C, the culture medium was aspirated from the transwell chambers. Cells that had traversed the membrane via invasion were subjected to fixation on the inferior surface of the membrane using a 4% paraformaldehyde solution. Subsequently, these cells were stained with a crystal violet solution. Five random fields were selected, and the number of invading cells per field was counted. Experiments were repeated three times, and the results were averaged.

#### 2.2.7. RT-qPCR assay.

The total RNA from the OSCC cells was extracted using Total cellular RNA extraction kit (G3640-50T,Servicebio,China). cDNA was generated using the Swescript Allin-one RT SuperMix for qPCR (G3337,Servicebio,China). RT-qPCR was performed with 2xUniversal Blue SYBR Green qPCR Master Mix(G3326,Servicebio,China) using the PCR Thermal Cycler (T100, Bio-Rad Laboratories,USA) following the manufacturer’s instructions. The relative gene expressions were measured via the 2^−ΔΔCt^ method and standardized to the expression of β-Actin. Table 1 shows the sequences of the forward and reverse primers of hub genes.

**Table 1:** The hub genes’ primer sequences were used for RT-qPCR assay.

Gene symbol	Primer	Primer Sequence (5’-3’)
CDK1	Primer_F	AGGAACAATTAAACTGGCTGATTT
	Primer_R	TGCCTATACTCCAAATGTCAACT
MTFR2	Primer_F	GAGTGTGGACCCAGATCAGC
	Primer_R	GCCAGGTGACCGCTCAAT
E-cadherin	Primer_F	CGAGAGCTACACGTTCACGG
	Primer_R	GGGTGTCGAGGGAAAAATAGG
N-cadherin	Primer_F	AGCCAACCTTAACTGAGGAGT
	Primer_R	GGCAAGTTGATTGGAGGGATG
β-Actin	Primer_F	TGACGTGGACATCCGCAAAG
	Primer_R	CTGGAAGGTGGACAGCGAGG

#### 2.2.8. Western blot analysis.

Proteins extracted from OSCC cells were subjected to 10% sodium dodecyl sulfate-polyacrylamide gel electrophoresis (SDS-PAGE) and subsequently transferred to PVDF membranes (Beyotime, China). After blocking for 10 minutes with protein-free rapid blocking solution (03891800, Epizyme Biotech, China), the primary antibodies were added and incubated at 4°C overnight. The secondary antibody was subjected to an incubation process at ambient temperature for a duration of 2 hours. Finally, the protein bands were determined under the Ultrasensitive ELC chemiluminescence kit(G2020, Servicebio).Bands were semi-quantified using densitometric analysis with ImageJ software.

The antibodies: CDK1(1:1000,33−1800), MTFR2(1:500, MA5−27461) were purchased from ThermoFisher. β-Actin (1:20000, HA722023), E-cadherin (1:5000, ET1607−75), N-cadherin (1:1000, ET1607−37) antibodies were purchased from HUABIO. HRP Conjugated AffiniPure Goat Anti-mouse IgG (H + L) and HRP Conjugated AffiniPure Goat Anti-rabbit IgG(H + L) were purchased from BOSTER Biological Technology.

#### 2.2.9. OSCC xenografts in Nude mice.

The nude mice were sourced from Hangzhou Ziyuan Laboratory Animal Technology Co., Ltd. (Hangzhou, China). The Animal Welfare Ethics Committee of Jiangxi University of Chinese Medicine approved this experiment (Approval No.20250303026). CAL 27 cells (1 × 10^7^/0.2 ml) were subcutaneously injected into the right flank of 4-week-old female BALB/c nude mice. When the maximum tumor diameter reached 5 mm, the tumor-bearing nude mice were randomly divided into 4 groups: the control group, the Blank nanoemulsion (Blank-NEs) treated group, the AS/BJO-NEs treated group, and the Cisplatin treated group (n = 3/group). The control group (normal saline 50 mg/kg), the Blank-NEs group (Blank-NEs 50 mg/kg), the AS/BJO-NEs group (AS/BJO-NEs 50 mg/kg), and the Cisplatin group (Cisplatin 5 mg/kg) were intraperitoneally injected every two days for a total of 10 times. During this period, all mice were examined every 3 days to evaluate their health status and any evidence of drug toxicity. Tumors were measured every 3 days using standard calipers, and tumor volume was calculated as ½length×width^2^. Tumor growth curves were obtained as previously described. At the end of the experiment, the tumors were separated from the surrounding muscle and dermis and then weighed.

#### 2.2.10. Immunohistochemical (IHC) staining.

Tissue sections from xenografts were fixed and subjected to IHC analysis. Following dewaxing and rehydration, the tissue sections were immersed in sodium citrate buffer (10 mM, pH 6.0) and boiled for 10 minutes. Subsequently, the tissue sections were incubated in 3% H_2_O_2_ in methanol for 10 minutes and washed with PBS. Blocking of the slides was then performed using 5% goat serum albumin, followed by incubation with the primary antibody at 4°C for 12 hours and the corresponding secondary antibody at room temperature for 45 minutes. The immune reaction was visualized using DAB substrate. Finally, hematoxylin was applied for counterstaining.

#### 2.2.11. Statistical analysis.

Kaplan-Meier analysis was undertaken via the log-rank tests in R software, and differences in gene expression were assessed using the Student’s t-tests. All statistical analyses and data visualization were carried out with the utilization of GraphPad Prism 9 software. Data are presented as the mean ± SD from at least three independent experiments. For quantitative comparisons, independent sample t-tests were used for two-group comparisons, while one-way ANOVA was utilized for conducting comparisons among multiple groups. P values< 0.05 were considered statistically significant. In the graphs, ns indicates no significance, * p < 0.05, ** p < 0.01, *** p < 0.001, and **** p < 0.0001.

## 3. Result

### 3.1. Part of network pharmacology

**Fig 1:**
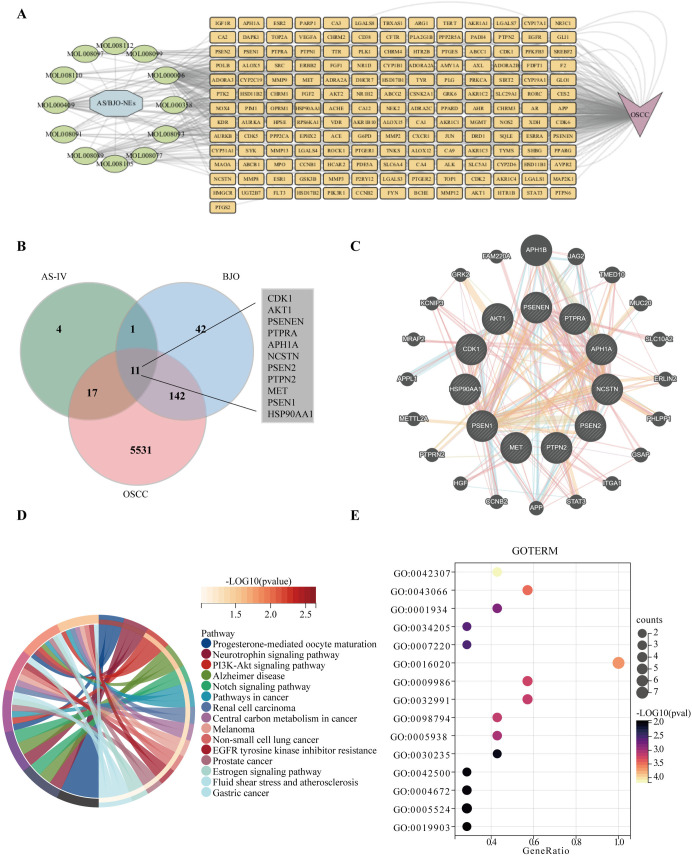
The potential targets and mechanism analysis of AS/BJO-NEs against oral squamous cell carcinoma (OSCC). **(A)** Drug-target-disease network diagram of AS/BJO-NEs and OSCC. **(B)** Venn diagram showing the triple intersection of the targets of the two components of AS/BJO-NEs and OSCC targets. **(C)** Visualization of PPI network diagram of intersection targets. **(D)** Visualization of the KEGG enrichment results(15 results are selected for display). **(E)** Visualization of the GO enrichment results(15 results are selected for display).

We identified a total of 12 effective components from TCMSP, including 11 from Brucea javanica and AS-IV (the active component of Astragalus membranaceus). After obtaining their SMILES identifiers from PubChem, we predicted potential targets using the SwissTargetPrediction platform. The targets related to OSCC were obtained from two disease databases, GeneCards and DisGeNET. We then performed ID mapping using UniProt’s module by inputting the target gene names (in gene symbol format), selecting Homo sapiens as the species, and choosing UniProtKB as the target database. After removing unmappable targets and standardizing the data, we finally obtained 230 targets for AS/BJO-NEs (34 for AS-IV and 196 for Brucea javanica) and 5622 OSCC-related targets. The active ingredients of the drugs, their corresponding targets, and the disease-related targets were visualized as a drug-target-disease network diagram (Fig 1A) using Cytoscape 3.10.1 software. The targets of the two components of AS/BJO-NEs and those of OSCC were intersected through R language to obtain a total of 11 common targets among the three parties,and a Venn diagram was made (Fig 1B). PPI network of intersecting targets between OSCC and AS/BJO-NEs was constructed using GeneMANIA (Fig 1C), presenting some of the more strongly correlated associated proteins. KEGG and GO enrichment analyses were performed on the 11 common targets by employing the DAVID analysis platform. The results indicated that in KEGG, the major enrichments were observed in pathways like the PI3K-Akt pathway, Notch pathway, pathways in cancer. In GO, the principal enrichments occurred in biological processes such as glucose homeostasis, mitochondrial transport, phosphorylation, and positive regulation of protein (Fig 1D–E).

### 3.2. Cluster analysis of EMT prognosis-related genes based on TCGA-OSCC samples

Through the GSEA database, we obtained 27 EMT-related gene sets containing a total of 1,463 EMT-associated genes. Subsequently, we performed a univariate Coxsurvival analysis of these genes using the TCGA-OSCC transcriptome dataset and corresponding survival data, retaining only those genes that significantly affected survival (HR > 1, P < 0.05) and exhibited significant differential expression between OSCC and non-OSCC tissues in TCGA-OSCC (|log2FC| > 1, P < 0.05). Ultimately, we identified 49 EMT-related genes with a significant impact on OSCC patient survival prognosis for inclusion in subsequent clustering analysis. Subsequently, the obtained genes were subjected to P-value adjustment using the Benjamini-Hochberg procedure, revealing that all 49 genes maintained statistical significance (S2 Table). Furthermore, after controlling for potential confounding factors including patient sex, age, and tumor clinical grade, multivariate Cox proportional hazards analysis was performed to evaluate whether each of these genes served as an independent prognostic factor for survival outcomes (S2 File). This analysis ultimately identified 46 epithelial-mesenchymal transition (EMT)-related genes with significant prognostic value (S3 Table). We utilized the “ConsensusClusterPlus” and “limma” packages, inputting 357 TCGA-OSCC samples containing expression data for 46 EMT genes. Based on the functions available, we selected the “km” clustering method, used the “euclidean” method to calculate distances, and set parameters such as 1,000 sampling times for unsupervised clustering analysis. Through this process, we obtained EMT-related subclasses and ultimately chose a two-class classification. Based on unsupervised clustering analysis, TCGA-OSCC samples were divided into different subcategories, and the consensus score of the CDF curve indicated that K = 2 was the optimal. KM survival analysis revealed significant differences between the two subgroups (EMT-cluster1 and EMT-cluster2) (S1 FIG). The results demonstrated that the EMT-cluster1 exhibited a poorer survival prognosis (S1 FIG). To delve deeper into the biological functions and regulatory mechanisms underlying the two EMT subgroups, we performed GSEA enrichment analysis using the predefined gene sets h.all.v2024.1.Hs.symbols.gmt. The results of the difference analysis during the process are presented in S4 Table. The enrichment analysis revealed that EMT-cluster1 exhibited higher activation levels in Epithelial-Mesenchymal Transition, E2F Targets, and Angiogenesis,etc., compared to EMT-cluster2. Conversely, the activation levels of Myogenesis and Bile Acid Metabolism were lower (S1 FIG).

### 3.3. WGCNA identifies targets associated with OSCC clinical phenotypes and the poor-prognosis EMT subtype

The TCGA-OSCC transcriptome data were selected, with the criteria of |LOG2FC>1| and P < 0.05. Through differential analysis by the limma package, a cumulative count of 8,877 differentially expressed genes (DEGs) were obtained (S5 Table), among which 4,818 were up-regulated genes and 4,059 were down-Regulated genes. To conduct the WGCNA analysis, we incorporated the tumor, non-tumor, and EMT subgroups from the TCGA-OSCC dataset as distinct phenotypes. We selected the previously identified DEGs and set a soft threshold of β = 9, with a minimum module size of 50. Clustering was conducted based on topological overlap and module color, and the genes were clustered into 12 different consensus gene modules according to their expression patterns. An association matrix between the consensus gene modules and clinical information was established. Correlation analysis reveals that genes in the yellow and brown modules exhibit the strongest positive correlation with both the tumor phenotype and the poor-prognosis EMT-cluster1. The yellow module exhibits a correlation coefficient r of 0.58 with the tumor phenotype (P < 0.001) and an r value of 0.70 with the EMT-cluster1 subgroup (P < 0.001). The brown module shows an r value of 0.51 with the tumor phenotype (P < 0.001) and an r value of 0.44 with the EMT-cluster1 subgroup (P < 0.001). Based on these findings, the yellow and brown modules were identified as key modules for subsequent analysis. To further elucidate the biological functions and regulatory mechanisms of these two gene modules, we performed a functional enrichment analysis. The results revealed that genes in the yellow module were significantly associated with pathways like cell cycle, DNA replication, p53 pathway, and FoxO pathway. The genes in the brown module were enriched in ECM-receptor interaction, PI3K-Akt pathway, and Wnt pathway,etc. (S2 FIG).

### 3.4. Analysis of the potential mechanism of AS/BJO-NEs in inhibiting the EMT process of OSCC via suppressing MTFR2

Using the TCGA – OSCC transcriptome data as a basis, we explored the differential expression of MTFR2 between OSCC samples and control samples. ROC analysis demonstrates that MTFR2 exhibits strong discriminatory power between tumor and non-tumor tissues in the TCGA-OSCC samples, with good predictive accuracy (AUC: 0.957). Meanwhile, we integrated survival data of the samples to assess the impact of MTFR2 expression on OS, disease-specific survival (DSS), progression free interval (PFI), and disease-free interval survival (DFI). Additionally, using the TCGA-PanCancer pan-cancer transcriptome data, we contrasted the differential MTFR2 expression between various tumor types and their corresponding normal control groups. The above results demonstrate that MTFR2 is significantly overexpressed in tumor samples and exhibits a strong negative correlation with OS, DSS, and DFIS of patients. These findings further support our previous study, which demonstrated that MTFR2 is related to adverse phenotypes in OSCC. Elevated expression of MTFR2 promotes the progression of OSCC and may contribute to its poor prognosis.

Next, based on the GSE37991 sample dataset, we performed differential expression analysis using the limma package. The screening criterion was set as |log2FC| > 0.5, resulting in the identification of 4,607 DEGs for further analysis (S6 Table). We stratified the GSE37991 samples into MTFR2 high-expression and low-expression groups according to the median MTFR2 expression level. Then, WGCNA was performed on the above DEGs, with a soft threshold of β = 22 selected and the minimum module size set at 50. Ultimately, five modules were generated. Among them, the genes within the yellow module showed a strong positive association with both clinical phenotypes of the GSE37991 dataset and the high-expression group of MTFR2 (r = 0.63, P < 0.001 for OSCC phenotype; r = 0.67, P < 0.001 for the high-expression MTFR2 group). This suggested that the yellow module might contain genes capable of up-regulating MTFR2, thereby promoting EMT-related processes in OSCC. Next, we conducted a functional enrichment analysis on the genes within yellow module. The results indicated that KEGG enrichment was primarily associated with pathways including cell cycle, Oocyte meiosis, DNA replication, and p53 pathway. GO enrichment analysis revealed notable associations with biological processes including cell division, ATP binding, ATP hydrolysis activity, and protein binding.

Finally, we overlapped the genes selected from the two WGCNA analyses with the drug-disease target genes obtained through network pharmacology, ultimately identifying one core target gene, Cyclin-dependent kinase 1 (CDK1) (S3 FIG). Drawing on the aforementioned results, we hypothesize that CDK1 may promote the EMT-related malignant progression of OSCC by up-regulating MTFR2 expression. In contrast, the AS/BJO-NEs may exert its anti-tumor effects by regulating CDK1 and down-regulating MTFR2, thereby inhibiting OSCC EMT and associated processes.

### 3.5. Analysis related to the single gene CDK1

**Fig 2:**
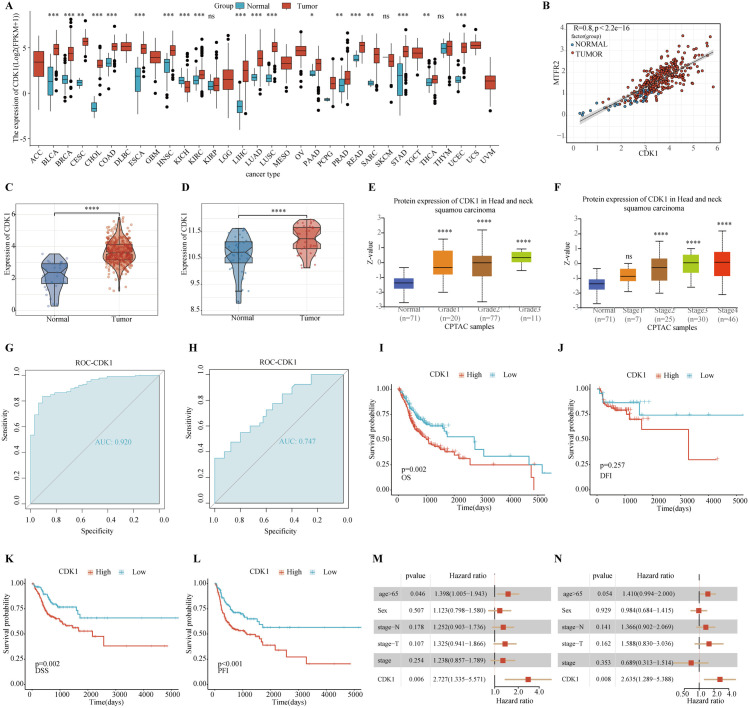
Single-gene bioinformatics analysis of CDK1. **(A)** Box plot of the differential expression of CDK1 between tumors and non-tumors in pan-cancer analysis. **(B)** CDK1 and MTFR2 exhibit a strong positive correlation (R = 0.8, P < 0.001). **(C-D)** Visualization of CDK1 protein expression across different tumor stages in HNSCC. **(E)** Differential expression of CDK1 between OSCC samples and normal controls in TCGA-OSCC transcriptome data. **(F)** Differential expression of CDK1 between OSCC samples and normal controls in GSE37991 transcriptome data. **(G)** ROC curve analysis of CDK1 based on TCGA-OSCC transcriptome data. **(H)** ROC curve analysis of CDK1 based on GSE37991 transcriptome data. **(I)** Overall survival prognosis analysis of CDK1, P < 0.05. **(J)** Disease-free interval survival analysis of CDK1, P < 0.05. **(K)** Disease-Specific Survival analysis of CDK1, P < 0.05. **(L)** Progression-Free Interval analysis of CDK1, P < 0.05. **(M-N)** Based on the TCGA-OSCC dataset and its clinical data (survival data, age, sex, etc.), univariate (M) and multivariate Coxanalyses (N) were performed on CDK1, and the expression of CDK1 was still significantly negatively correlated with the poor prognosis of the patients.

Through TCGA-PanCancer analysis, we indicated that CDK1 exhibited a high-level of expression in tumors such as HNSCC compared with normal tissues (Fig 2A). Correlation analysis revealed a strong positive correlation between CDK1 and MTFR2 (Fig 2B). Further, UALCAN protein expression analysis demonstrated that CDK1 protein levels were elevated across all tumor stages relative to normal samples (Fig 2C–D). Additionally, we analyzed the differences in CDK1 expression between OSCC samples and normal controls using data from the TCGA-OSCC database and GSE37991 transcriptome dataset, results indicated statistical significance (Fig 2E–F). Fig 2G–H illustrated the predictive precision of CDK1 in distinguishing the cancer status of samples, The area under the curve (AUC) was found to surpass 0.7, which is indicative of its robust discriminatory power and favorable sensitivity. In addition, we performed a Kaplan-Meier analysis using survival data from the TCGA-OSCC database(Fig 2I–L). Furthermore, to more accurately illustrate the impact of CDK1 on OSCC survival prognosis, we added univariate/multivariate Coxanalysis, which incorporated age, tumor grade, and stage to correct for the effects of potentially confounding factors such as age, tumor grade, and stage. The results showed that high CDK1 expression was significantly associated with shorter OS, DSS, and PFI in OSCC patients, a result supported by the unifactorial/multifactorial Cox (Fig 2M, N).

### 3.6. Molecular docking and MDS

We performed molecular docking of luteolin (an effective component of Brucea javanica and involving CDK1 in the target gene prediction) and AS-IV with CDK1 respectively. The results revealed that the binding energy of the AS-IV-CDK1 complex was −5.15kJ/mol, while that of the luteolin-CDK1 complex was −6.3 kJ/mol. Both small molecule ligands exhibited favorable binding stability with CDK1(<−5KJ/mol). These results suggested that the active components of AS/BJO-NEs may exert their anti-OSCC effects through tight binding to CDK1.

**Fig 3:**
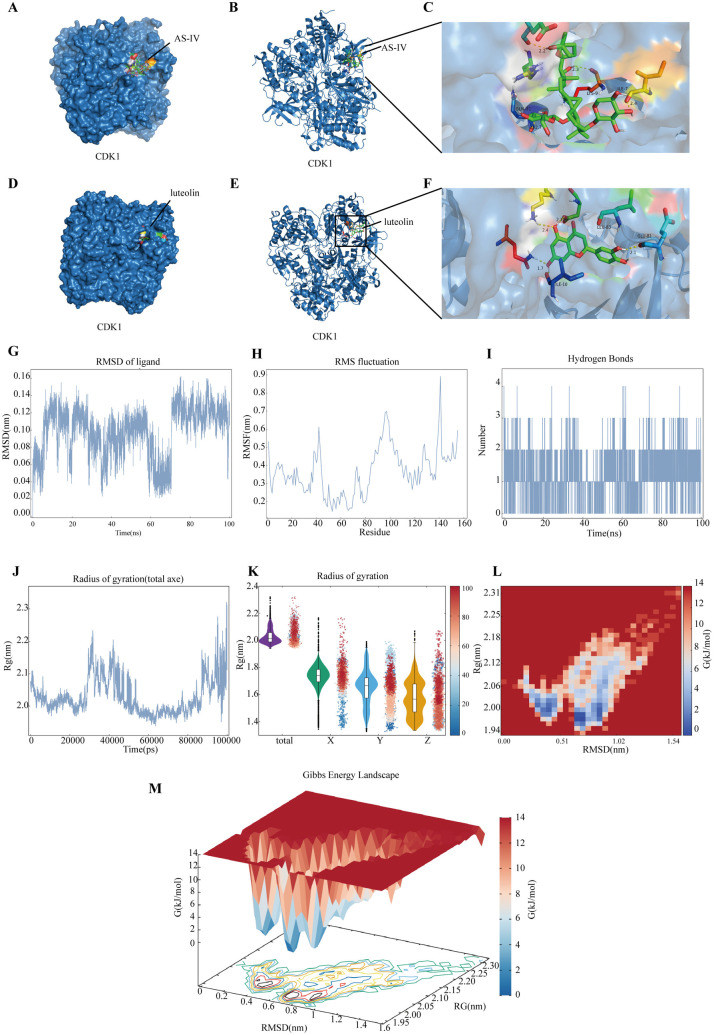
Molecular Docking and MDS. **(A-C)** Molecular docking complex of AS-IV and CDK1. **(D-F)** Molecular docking complex of luteolin and CDK1.**(G)** RMSD of ligand. **(H)** RMSF of chain A that binding with ligand. **(I)** Hydrogen bonds generated between ligand and protein. **(J)** RG of total axe of protein. **(K)** RG of total and around axes of protein. **(L)** 2D Gibbs free energy landscape of chain A in complex. **(M)** 3D Gibbs free energy landscape of chain A in complex.

Subsequently, we selected the luteolin-CDK1 complex with the lowest binding energy for MDS. In the MDS, we evaluated the binding stability of luteolin and CDK1 using Root Mean Square Deviation (RMSD), Root Mean Square Fluctuation (RMSF), and Radius of Gyration (RG).It can be observed that the atomic coordinates of the ligand rapidly stabilized at the very beginning of the simulation (Fig 3G), and almost no significant fluctuations occurred throughout the simulation period, suggesting that the docking position where the ligand is located can form a tight connection with the protein. Through RMSF analysis of the amino acid displacement of the chain (viz. chain A) that forms the greatest number of non-covalent interactions between proteins and small molecules (Fig 3H), it was discovered that the atomic displacement fluctuations near amino acids 35–45, 80–117, 128, and 141 of chain A were relatively obvious, suggesting that this region may potentially be conformationally unstable. This is ascribed to the frequent formation of non-covalent interactions between small molecules and residues, which results in flexible conformational alterations of these residues. Hence, we conjecture that this region is either a flexible region or a remote allosteric region. During the simulation, it was observed that 2–3 hydrogen bonds typically formed between the small molecule and the protein (Fig 3I). This bond formation facilitated the binding of the ligand to the receptor and helps maintain the stability of the system’s structure. Additionally, the protein’s radius of gyration was monitored throughout the simulation and displayed two noticeable fluctuations (Fig 3J), with increases noted between 30–53 ns and 90–100 ns. Analysis of three-dimensional metrics using violin plots and scatter plots (Fig 3K) revealed that the protein expanded in the Y and Z directions. The likely causes of these fluctuations was the discontinuity of chain A and the presence of a homologous H chain. Chain A had a deletion of 10 amino acid residues from positions 155–165, which resulted in less stable interactions between the two incomplete chains compared to covalent bonds. Consequently, significant positional fluctuations were more prone to occurring at the C-terminus of the upper chain and the N-terminus of the lower chain. Examination of the overall coordinates showed that the protein’s spatial variation was minimal (less than 0.2nm), indicating that the fluctuations in the Y and Z directions were sporadic and short-lived, and did not impact the protein’s overall shape. In general, the protein-ligand complex remained fairly stable. We then chose the 80–100 ns interval for calculating the Molecular Mechanics/Poisson-Boltzmann Surface Area (MMPBSA), representing a stable binding state between the small molecule and the protein (S7 Table). A 3D Gibbs free energy landscape diagram revealed two distinct potential wells during the simulation (Fig 3L–M), indicating that the MD simulation had successfully predicted the protein’s structure at its lowest energy state. The broad RMSD and RG intervals covered by these potential wells suggested that chain A of the protein remained in a low-energy state for a significant portion of the simulation.

### 3.7. AS/BJO-NEs inhibits the proliferative capacity of OSCC cells

**Fig 4:**
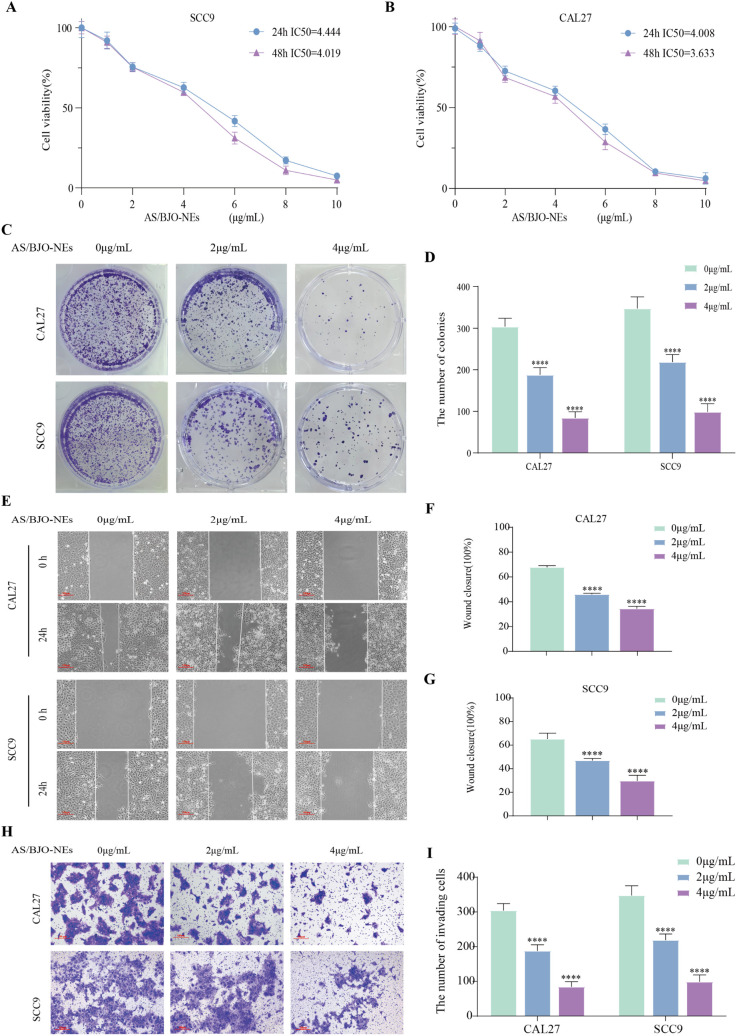
AS/BJO-NEs inhibited the proliferation, migration and invasion of OSCC cells. **(A-B)** CCK −8 assay was used to detect the effects of AS/BJO-NEs at different concentrations on the viability of SCC9 and CAL27 cells. **(C-D)** Clone formation assay was employed to examine the influence of AS/BJO-NEs on the proliferation ability of SCC9 and CAL27 cells. **(E-G)** Wound healing assay was conducted to investigate the impact of AS/BJO-NEs on the migration ability of SCC9 and CAL27 cells. **(H-I)** Transwell assay was utilized to evaluate the influence of AS/BJO-NEs on the invasion ability of SCC9 and CAL27 cells. n = 3, Scale bar: 100 μm, *p < 0.05, **p < 0.01, ***p < 0.001,and ****p < 0.0001.

Initially, we utilized the CCK-8 assay to assess the influence of AS/BJO-NEs at varying concentrations on the viability of OSCC cell lines(SCC9 and CAL27 cells). As depicted in Fig 4A–B, AS/BJO-NEs markedly inhibited the viability of OSCC cells, and the antiproliferative effect of AS/BJO-NEs on OSCC cells was dose-dependent. Furthermore, for CAL27 cells, the 24-hour half – maximal inhibitory concentration (IC50) was determined to be 4.008 μg/mL, while the 48-hour IC50 value was 3.633 μg/mL. Regarding SCC9 cells, the 24-hour IC50 was 4.444 μg/mL, and the 48-hour IC50 was 4.019 μg/mL.Consequently, based on the above results, we selected AS/BJO-NEs at a concentration of 2 μg/mL as the low-concentration group and 4 μg/mL as the high-concentration group for subsequent experiments. Meanwhile, the colony formation assay was utilized once again to ascertain the cell proliferation. After the cells were treated with AS/BJO-NEs at low and high concentrations respectively, the number of colonies formed was calculated. In comparison to the control group,the number of colonies formed by cells in the experimental group was significantly decreased, with a statistically significant difference (Fig 4C–D). The results corresponded to CCK-8 assay, indicating that AS/BJO-NEs inhibited the proliferation ability of OSCC cells.

### 3.8. AS/BJO-NEs inhibits the migratory and invasive capabilities of OSCC cells

To elucidate the influence of AS/BJO-NEs on migration and invasion of OSCC cells, SCC9 and CAL27 cells were subjected to low and high concentrations of AS/BJO-NEs for 24 hours prior to conducting wound healing assay and Transwell assay. The outcomes obtained from the wound healing assay indicated that the cell migration rate was significantly suppressed following treatment with AS/BJO-NEs (Fig 4E–G). Meanwhile, the Transwell assay results manifested that the number of OSCC cells traversing the membrane of the upper chamber was reduced upon treatment with AS/BJO-NEs (Fig 4H–I). These results indicated that AS/BJO-NEs could inhibit the migration and invasion abilities of OSCC cells, and inhibitory effect on the migration and invasion of OSCC cells is more pronounced in the high-concentration group compared to the low-concentration group.

### 3.9. AS/BJO-NE downregulates the expression of CDK1 and MTFR2 and inhibits the progression of EMT

**Fig 5:**
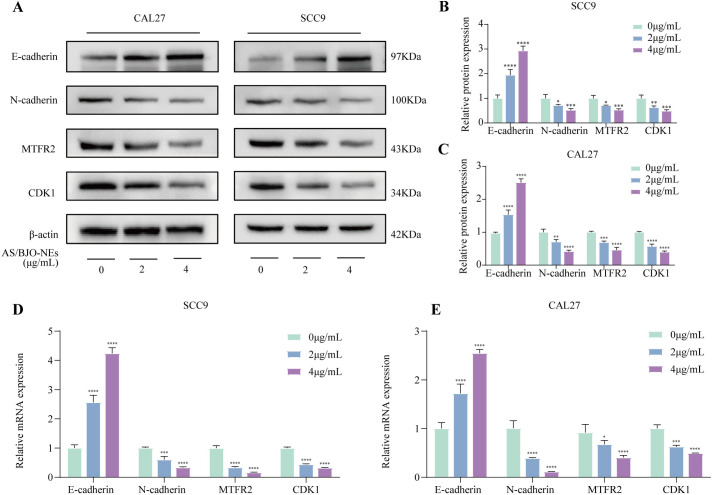
Western blot and RT-qPCR experiments were conducted to detect the expression levels of related molecules. **(A-C)** Western blot assessment of the CDK1,MTFR2 and EMT markers (E-cadherin,N-cadherin)protein levels in SCC9 and CAL27 cells intervened with AS/BJO-NEs(0,2,4 μg/ml) for 24h. **(D-E)** RT-qPCR assessment of the mRNA level of the CDK1,MTFR2 and EMT markers(E-cadherin,N-cadherin) in SCC9 and CAL27 cells intervened with AS/BJO-NEs (0,2,4 μg/ml) for 24h. n = 3,*P < 0.05, **P < 0.01, ***P < 0.001, and ****P < 0.0001.

The expression levels of CDK1, MTFR2 and EMT-related molecules after treatment with AS/BJO-NEs were further verified through Western Blot and RT-PCR experiments.As illustrated in Fig 5A–C, treatment with AS/BJO-NEs in SCC9 and CAL27 cells resulted in a notabe upregulation of E-cadherin protein expression and a concurrent inhibition of CDK1, MTFR2, and N-cadherin protein levels in comparison with the control group. RT-qPCR experiments (Fig 5D–E) demonstrated that the mRNA expression trends were consistent with the protein expression: E-cadherin mRNA expression increased, while CDK1, MTFR2, and N-cadherin mRNA expression decreased. The high-concentration group showed more significant effects compared to the low-concentration group.These results indicated that AS/BJO-NEs could significantly inhibit the proliferation, migration and invasion of OSCC cells, down-regulated the expression of CDK1 and MTFR2, and suppressed the EMT process.

### 3.10. Successful construction of stable CAL27 cell lines with knockdown and overexpression of CDK1

**Fig 6:**
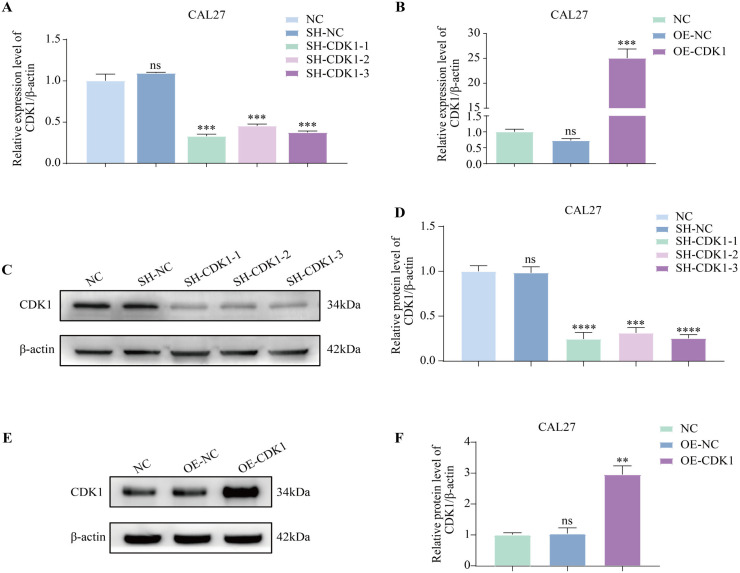
RT-qPCR and Western blot assays validate the knockdown and overexpression efficiency of CDK1. **(A)** RT-qPCR assays were utilized to examine the CDK1 mRNA expression after knockdown. **(B)** RT-qPCR assays were utilized to examine the CDK1 mRNA expression subsequent to overexpression. **(C-D)** Western blotting experiments were carried out to evaluate the CDK1 protein expression after knockdown. **(E-F)** Western blotting experiments were carried out to evaluate the CDK1 protein expression following overexpression. n = 3, *p < 0.05, **p < 0.01, ***p < 0.001, and ****p < 0.0001.

Next, after establishing the stable CAL27 cell lines with CDK1 knockdown and CDK1 overexpression, we classified them into the SH-CDK1–1 group, SH-CDK1–2 group, SH-CDK1–3 group and OE-CDK1 group. Moreover, the corresponding empty vectors were constructed as controls (the SH-NC group and the OE-NC group). RT-qPCR and Western blot assays were applied to validate the transfection efficiency. The outcomes of RT-qPCR (Fig 6A–B) and Western blot (Fig 6C–F) experiments demonstrated that there was no notable discrepancy in the CDK1 mRNA expression and protein between the SH-NC group, the OE-NC group and negative control group (NC group) (P > 0.05). The expressions of CDK1 mRNA and protein in the SH-CDK1–1 group, the SH-CDK1–2 group and the SH-CDK1–3 group were notably decreased compared with the NC group, while the expression of CDK1 in the OE-CDK1 group was remarkably elevated, confirming the successful transfection. Among them, the SH-CDK1–1 group exhibited the most optimal knockdown effect. Therefore, we selected the SH-CDK1–1 group for the ensuing experiments.

### 3.11. The effects of knockdown/overexpression of CDK1 on the proliferation, migration and invasion of OSCC cells

**Fig 7:**
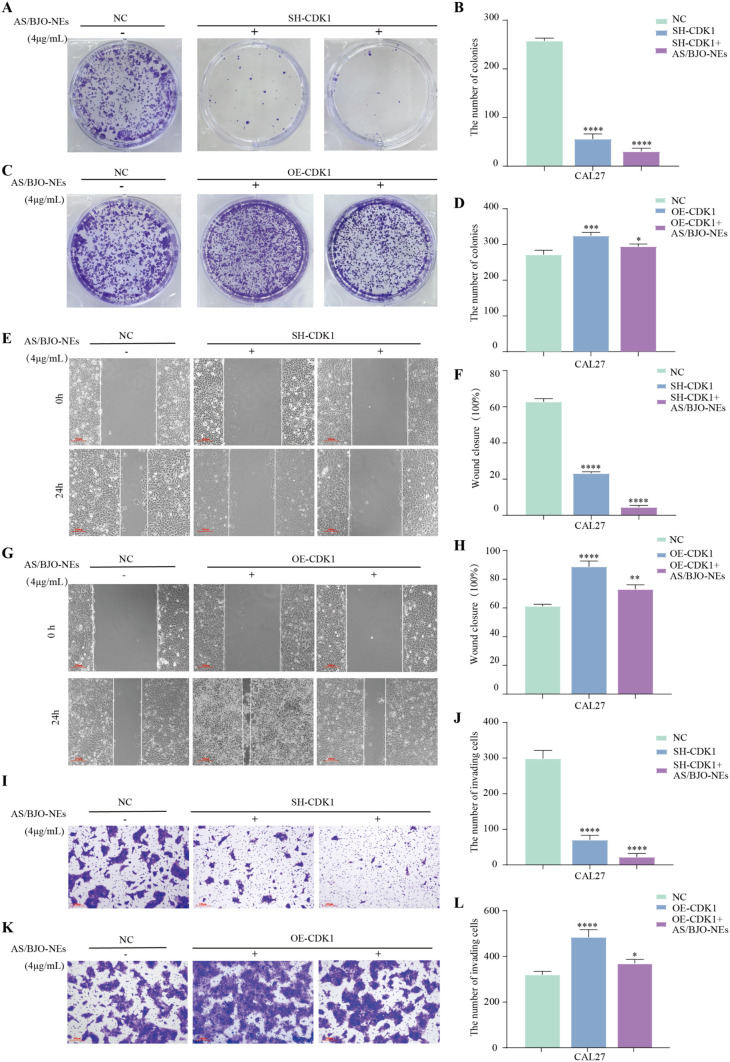
The Effects of CDK1 Knockdown and Overexpression on the Proliferation, Migration and Invasion of CAL27 Cells. **(A-B)** The clone formation assay was utilized to investigate the impact of CDK1 knockdown on the proliferation of CAL27 cells. **(C-D)** The clone formation assay was utilized to investigate the impact of CDK1 overexpression on the proliferation of CAL27 cells. **(E-F)** Wound healing assay was applied to assess the influence of CDK1 Knockdown on the migration of CAL27 cells. **(G-H)** Wound healing assay was applied to assess the influence of CDK1 overexpression on the migration of CAL27 cells. **(I-J)** Transwell assay was utilized to explore the influence of CDK1 Knockdown on the invasion of CAL27 cells. **(K-L)** Transwell assay was carried out to explore the impact of CDK1 overexpression on the invasion of CAL27 cells. n = 3,Scale bar: 100μm, *p < 0.05, **p < 0.01, ***p < 0.001, and ****p < 0.0001.

The CAL27 cells were categorized into the control group, the SH-CDK1 group, the SH-CDK1+AS/BJO-NEs group, the OE-CDK1 group and the OE-CDK1+AS/BJO-NEs group. The findings from the colony formation assay indicated that the quantity of cell colonies in the SH-CDK1 group was markedly reduced compared to the control group, indicating that knockdown of CDK1 could suppress the proliferation ability of OSCC cells. Moreover, the suppressive effect was more obvious when combined with AS/BJO-NEs (Fig 7A–B).Conversely, overexpression of CDK1 promoted cell proliferation, and this proliferative effect was attenuated by treatment with AS/BJO-NEs (Fig 7C–D).The results of wound healing assay and Transwell experiment indicated that, when contrasted the control group, the migration rate and the quantity of invasive cells in the SH-CDK1 group declined conspicuously, indicating that knockdown of CDK1 could restrain the migration and invasion capabilities of OSCC cells. The inhibitory effect was more remarkable when combined with AS/BJO-NEs (Fig 7E–F, I–J).It has been demonstrated that the overexpression of CDK1 can potentiate the cellular capabilities of migration and invasion. Treatment with AS/BJO-NEs, however, can effectively inhibit this promoting effect (Fig 7G–H, K–L).

### 3.12. AS/BJO-NEs inhibit the proliferation, migration and invasion of OSCC cells and the progression of EMT by specifically down-regulating CDK1 and subsequently reducing MTFR2 expression

**Fig 8:**
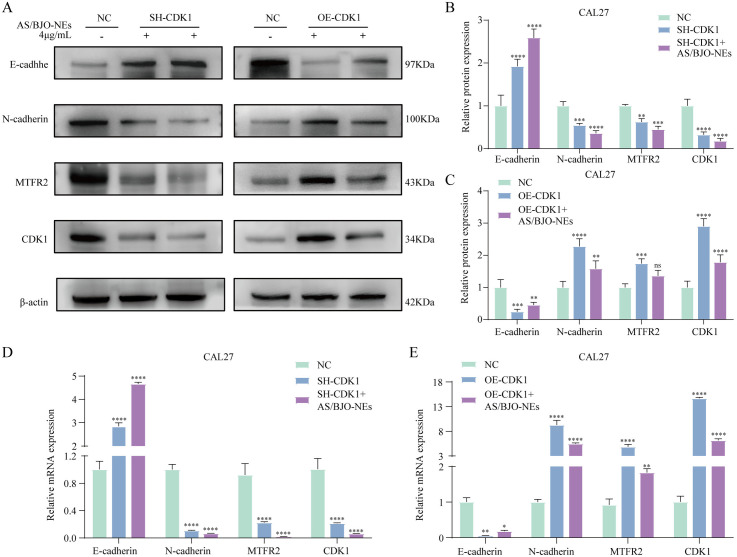
Western blot and RT-qPCR experiments were conducted to detect the expression levels of related molecules. **(A-C)** Western blot assessment of the CDK1,MTFR2,E-cadherin,and N-cadherin protein levels. **(D-E)** RT-qPCR assessment of the CDK1,MTFR2,E-cadherin,and N-cadherin mRNA levels. n = 3,*P < 0.05, **P < 0.01, ***P < 0.001, and ****P < 0.0001.

The Western Blot results (Fig 8A–C) demonstrated that the expression levels of MTFR2 and N-cadherin proteins in the SH-CDK1 group were significantly lower compared to the control group, while E-cadherin expression was notably higher. In addition, the protein expression levels in the SH-CDK1+AS/BJO-NEs group exhibited more pronounced changes, with certain proteins showing either a significant decrease or increase.In comparison with the control group, in the OE - CDK1 group, the expression levels of MTFR2 and N-cadherin proteins were upregulated, while the expression level of E-cadherin was downregulated, with significant differences. Furthermore, in the OE-CDK1 + AS/BJO-NEs group compared to the control group, the expression of MTFR2 and N-cadherin proteins were increased, while the expression of E-cadherin protein was decreased. However, compared to the OE-CDK1 group, the OE-CDK1+AS/BJO-NEs group showed decreased expression of MTFR2 and N-cadherin proteins, while E-cadherin protein expression was increased. Likewise, RT-qPCR assays proved that the changing trend of mRNA expression was in accordance with that of protein expression (Fig 8D–E).

The aforementioned outcomes indicated that knockdown of CDK1 could downregulate the expression of MTFR2 and suppress the EMT process of OSCC cells; conversely, overexpression of CDK1 could upregulate the expression of MTFR2 and facilitate the EMT process of OSCC cells. Evidently, the expression of MTFR2 varies with the changes of CDK1. Following the administration of AS/BJO-NEs treatment, the inhibitory effect was more pronounced in the CDK1 knockdown group, whereas it attenuated the promoting effect in the overexpression group.

### 3.13. AS/BJO-NEs inhibits the growth of OSCC xenograft tumors in vivo

**Fig 9:**
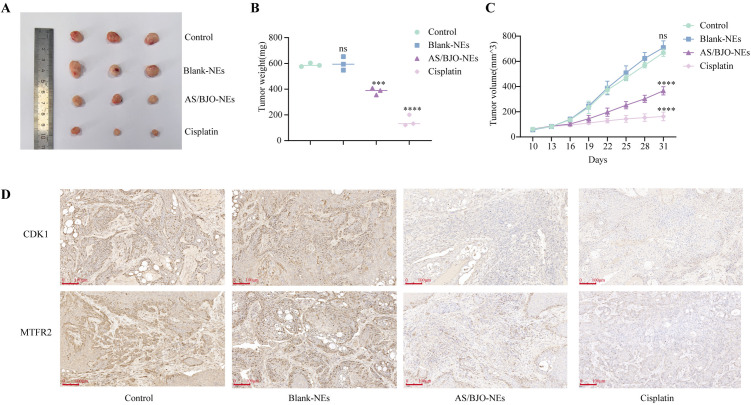
AS/BJO-NEs inhibited the growth of CAL27 xenograft tumors in nude mice. **(A)** Representative photographs of the tumors from nude mice. **(B)** The weights of tumors from the control groups, Blank-NEs treated groups, AS/BJO-NEs treated groups, and Cisplatin treated groups. **(C)** The growth curves of tumors derived from CAL27 xenografts in mice after normal saline, Blank-NEs, AS/BJO-NEs, and Cisplatin treatment. **(D)** The expression of CDK1 and MTFR2 were detected by immunohistochemical staining and the expression of CDK1 and MTFR2 were decreased after AS/BJO-NEs and Cisplatin treatment. Scale bar: 100μm, n = 3, *p < 0.05, **p < 0.01, ***p < 0.001, and ****p < 0.0001.

To investigate whether AS/BJO-NEs can inhibit tumor growth in vivo, we selected the CAL 27 cell line to establish xenograft tumors in nude mice. When the tumor volume reached above 50 mm^3^, the mice were randomly divided into four groups. As shown in Fig 9A–C, compared to the control group, both the AS/BJO-NEs treatment group and the cisplatin treatment group demonstrated significant inhibitory effects on tumor growth. Based on tumor volume, the tumor inhibition rate in the AS/BJO-NEs treatment group was 45.18% on day 31, while it was 75.67% in the cisplatin treatment group. The average weight of tumors resected from the AS/BJO-NEs treatment group was reduced by approximately 35% compared to the control group, and by approximately 71% in the cisplatin treatment group. Immunohistochemical analysis revealed that both AS/BJO-NEs and cisplatin could suppress the expression of CDK1 and MTFR2 in the xenograft tumors (Fig 9D).

**Fig 10:**
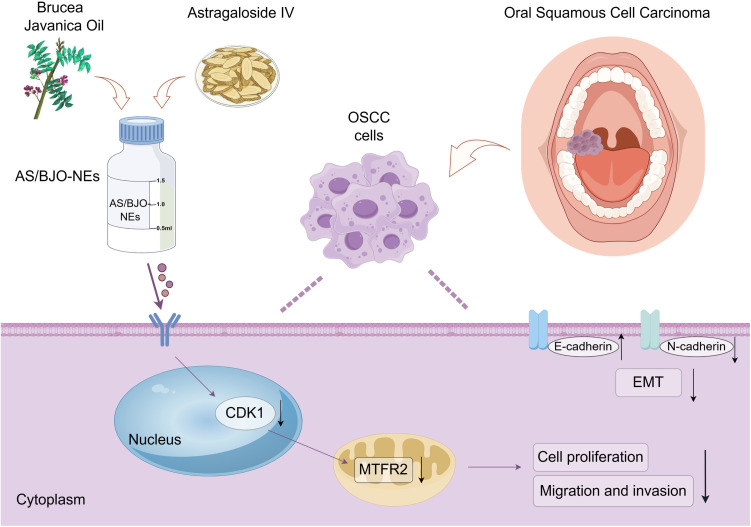
Mechanism of AS/BJO-NEs on OSCC. AS/BJO-NEs downregulate CDK1, and then reduce the expression of MTFR2, thereby inhibiting the proliferation, migration, invasion as well as the EMT process of OSCC cells.

Incorporating the aforementioned outcomes, it is substantiated that AS/BJO-NEs suppress the proliferation, migration, invasion and EMT progression of OSCC cells by targeting and down-regulating CDK1 and reducing the expression of MTFR2(Fig 10).

## 4. Discussion

OSCC can be induced by various factors, including prolonged exposure to numerous potential carcinogens or an unwholesome lifestyle, and its incidence has been on the rise year by year [36]. Although there are diverse treatment approaches available for oral cancer, encompassing surgery, radiotherapy, chemotherapy, and targeted therapy, issues such as a high recurrence rate and unfavorable prognosis still exist [37]. In recent years, TCM has demonstrated favorable anti-tumor effects owing to its characteristics of multi-target action and minor adverse reactions, which is capable of extending the survival duration and enhancing the life quality of cancer patients [38]. Particularly in the case of OSCC, TCM has shown broad application prospects in both treatment and prognosis. Li Xie et al.[39]discovered that gastrodin, an active component of Gastrodia elata, can inhibit glycolysis in OSCC cells and suppress the progression of chemotherapy resistance of OSCC cells in vivo, especially when combined with Skp2 inhibitors. It can be seen that in-depth exploration of the application of TCM in OSCC helps to uncover more therapeutic potential and provide new drug options for clinical practice.

To explore more effective TCM treatments for OSCC, we successfully prepared AS/BJO-NEs by high-pressure homogenization of AS-IV and BJO, aiming to provide new therapeutic strategies for OSCC. Currently, BJO, as a widely used anti-tumor TCM preparation in clinical practice, demonstrates certain advantages over conventional chemotherapy drugs in improving life – quality and extending survival period of patients with advanced cancer [40]. AS-IV, owing to its antioxidant, anti-inflammatory, and anti-apoptotic properties, has shown significant effects in enhancing immune function, inhibiting cancer cell migration and invasion, and improving chemosensitivity [41].Additionally, recent studies have made notable progress in combining AS-IV with other TCM or chemical drug active ingredients [42]. TCM nano-preparations can simultaneously deliver multiple drugs to specific cells, tissues, or organs, thereby effectively enhancing the permeability and retention of drugs in solid tumor tissues [43,44]. In the current investigation, through in vitro experiments, we discovered that as the dosage of AS/BJO-NEs increased, the growth of OSCC cells was notably inhibited, and cell migration and invasion capabilities gradually weakened in a dose-dependent manner. Further RT-qPCR and Western Blot experiments revealed that molecules closely associated with the EMT process were modulated after treatment with AS/BJO-NEs, indicating that AS/BJO-NEs might exert their anti-tumor effects by inhibiting the EMT process. Nevertheless, the specific action pathways of AS/BJO-NEs in OSCC remain unclear. Hence, we employed a combined approach of network pharmacology and bioinformatics to explore the targeted regulatory mechanism of AS/BJO-NEs on OSCC.

Firstly, we used network pharmacology methods to screen a total of 11 core target genes of AS/BJO-NEs acting on OSCC. Subsequently, through pan-cancer transcriptome data, TCGA-OSCC transcriptome data, and clinical survival data, we conducted an analysis of the differential expression of MTFR2 across various tumors and the influence of high MTFR2 expression on the survival prognosis of OSCC patients. The results showed that MTFR2 exhibited high-level expression in tumor samples and displayed a significant negative correlation with the survival prediction of patients. This further confirmed our previous research: MTFR2 is associated with the adverse phenotypes of OSCC. Its elevated expression facilitates the onset and progression of OSCC and may be linked to the dismal prognosis of OSCC. Furthermore, through WGCNA, we identified gene modules associated with poor-prognosis subtypes of EMT and those linked to high expression of MTFR2. We then performed a correlation analysis between the genes selected from these WGCNA modules and the drug-disease intersection gene targets, identifying CDK1 as a core target gene. Bioinformatics analysis disclosed that CDK1 is remarkably expressed in tumors such as OSCC and exhibits a strong positive correlation with MTFR2. Additionally, elevated CDK1 expression significantly impacts the survival prognosis of OSCC patients. Molecular docking studies indicated that CDK1 can stably bind to AS-IV in AS/BJO-NEs and luteolin, an active ingredient of Brucea javanica, supporting our selection of CDK1 as the target gene. Subsequently, we conducted MDS on the CDK1-luteolin complex with the lowest binding energy, demonstrating stable ligand-receptor binding conformation. These findings further confirmed that the interaction between CDK1 and AS/BJO-NEs was characterized by strong affinity and stability.

CDK1 belongs to the cyclin - dependent kinases (CDKs) superfamily. As a major regulatory protein involved in cell cycle control, it assumes a critical function in the progression of the cell cycle cascade, which is often severely disrupted in cancer cells [45]. Existing studies have demonstrated that elevated expression of CDK1 is strongly correlated with the development and prognosis of various malignancies [46]. For example, in lung cancer, overexpression of CDK1 is suggestive of a dismal prognosis [47]. CDK1 also shows high expression in gastrointestinal stromal tumor(GIST) [48]. Additionally, multiple studies [,^49^,^50^] have shown that downregulating CDK1 can arrest the tumor cell cycle and inhibit tumorigenesis, development, and the EMT process. EMT is a cellular program wherein cells transition from an epithelial to a mesenchymal state, assuming a crucial function in the physiological processes and tumorigenesis of diverse cancers. Studies have shown that among all cancer cell subtypes, EMT is considered a reliable marker reflecting the malignant state [51]. During the EMT process, as OSCC cells lose cell polarity and cell adhesion molecules (such as E-cadherin), they acquire mesenchymal characteristics, such as a motile phenotype and the expression of mesenchymal markers (N-cadherin).Recent research has indicated that aberrant mitochondrial fission contributed to tumor progression [52].MTFR2, which is primarily involved in mitochondrial dynamics, particularly mitochondrial fission, can lead to imbalances in mitochondrial dynamics when abnormally expressed, thereby promoting cancer cell growth and proliferation. Cell migration and invasion during the EMT process necessitate substantial energy support. Functioning as the “energy-generating center” of the cell, mitochondria might supply energy to cells via the protein synthesis function ensured by MTFR2 and others [53].Additionally, in our previous research, it was discovered that BJO could suppress the proliferation, migration and invasion of OSCC cells by down-regulating MTFR2 and influencing the SOD2/H2O2 signaling pathway [14].

Based on the aforementioned bioinformatics analysis outcomes, we put forward the following hypothesis: AS/BJO-NEs can suppress the proliferation, migration, and invasion of OSCC cells as well as the EMT process by targeting and down-regulating CDK1 to reduce the expression level of MTFR2.We verified this supposition through in vitro experiments. RT-qPCR and WB experiments indicated that AS/BJO-NEs could decrease the expression levels of CDK1, MTFR2, and N-cadherin in OSCC cells and increase the expression level of E-cadherin. When CDK1 was knocked down, the MTFR2 and N-cadherin expression levels decreased, whereas the E-cadherin expression level rose; conversely, when CDK1 was overexpressed, the expression levels of MTFR2 and N-cadherin increased, and the expression level of E-cadherin declined. Evidently, the expression of MTFR2 varies with the alteration of CDK1. Secondly, after treatment with AS/BJO-NEs, the inhibitory effect was more significant in the CDK1 knockdown group, while in the overexpression group, its promoting effect could be inhibited. In vivo experiments, we established a xenograft tumor model of oral squamous cell carcinoma in nude mice, and the results indicated that AS/BJO-NEs significantly inhibited tumor growth in the nude mice. Immunohistochemical analysis further revealed that AS/BJO-NEs could suppress the expression of CDK1 and MTFR2 in the xenograft tumors. Based on the above experimental results, we believe that AS/BJO-NEs exert their anti-OSCC biological effects by downregulating the CDK1 expression and then regulating a series of tumor-related processes mediated by MTFR2, especially the inhibitory effect on the EMT process.

It is worth noting that although this study has verified the correlation between the expressions of CDK1 and MTFR2 through in vitro experiments, the specific regulatory mechanism between the two still needs further clarification. Based on literature reports, CDK1 can phosphorylate the histone H3K27 methyltransferase EZH2, leading to the instability of the PRC2 complex and the inhibition of H3K27 trimethylation, thereby activating the expression of HOX family genes [54]. In oral cancer research, HOX genes such as HOXC10 are continuously upregulated from precancerous lesions to malignant stages, and the HOXC10-encoded protein can bind to the MTFR2 promoter region to promote transcription [55]. Based on this, it is speculated that CDK1 may mediate transcriptional regulation through the “CDK1-EZH2-HOXC10-MTFR2” pathway, which provides a potential mechanistic direction for explaining the correlation between the two. Future research will be conducted to further explore this.

In addition, during the functional enrichment analysis of GO, KEGG, and GSEA, we found that the target genes of AS/BJO-NEs for anti-OSCC were mainly enriched in pathways such as PI3K-Akt pathway and Notch pathway. The differentially expressed genes between the two subgroups after clustering of EMT-related genes were mainly enriched in terms such as Epithelial-Mesenchymal-Transition, Angiogenesis, and Oxidative-Phosphorylation. The gene modules associated with the poor-prognosis subtypes of EMT and high expression of MTFR2 were enriched in pathways such as the Cell cycle, the p53 pathway, and the FoxO pathway. This indicates that the mechanism underlying CDK1’s regulation of the downstream functions of MTFR2 is complex rather than a single regulatory mode. Under the influence of tumorigenesis, development, and inflammation, highly expressed CDK1 may, while exerting an impact on Cell cycle and ECM-receptor interaction, act directly or indirectly on pathways such as the p53 pathway and the FoxO pathway, regulating the transcription and translation of MTFR2, leading to its activation and high expression. This thereby creates conditions for the initiation and maintenance of EMT and Angiogenesis, facilitating tumor proliferation, invasion, and metastasis. Moreover, AS/BJO-NEs can inhibit this series of processes by suppressing the expression of CDK1 and regulating MTFR2, thereby inhibiting the onset and progression of neoplasms, and modulating the tumor microenvironment and inflammatory environment.

Existing research has demonstrated that during the cell cycle, CDK1 holds a crucial regulatory role in mitochondrial function and can influence the proliferation and apoptosis of tumor cells by modulating mitochondrial bioenergetics. For example, p53 within mitochondria can be phosphorylated by CDK1, thereby inhibiting mitochondrial apoptosis and forming a feedback signaling pathway [56]. In esophageal cancer, the activation of p53 pathway can decrease CDK1 expression, resulting in G2/M cycle arrest, while promoting the generation of reactive oxygen species (ROS) and apoptosis [57]. This is consistent with the findings of our aforementioned enrichment analysis — CDK1 is associated with pathways such as the cell cycle and p53 pathway. Secondly, MTFR2 has received significant attention due to its prominent role in regulating mitochondrial fission and is expressed at abnormal levels in multiple cancer tissues, facilitating tumor progression. Additionally, relevant studies have indicated that in lung adenocarcinoma, there exists a close interaction among the cell cycle, DNA replication, the p53 pathway and MTFR2 [58]. Furthermore, this study identified potential pathways involved, including PI3K-Akt, Notch, and p53, through bioinformatics analysis, but experimental validation has not been conducted. Therefore, our future research will focus on conducting subsequent experiments to further clarify the regulatory mechanism of the anti-OSCC target core gene CDK1 of AS/BJO-NEs on MTFR2, as well as the activity changes of downstream related pathways.

## 5. Conclusion

In summary, through the combination of network pharmacology, bioinformatics and in vivo/in vitro experiments, this study has disclosed the potential molecular mechanism of AS/BJO-NEs against oral squamous cell carcinoma. We have verified that AS/BJO-NEs can play an anti-tumor role by down-regulating CDK1 and subsequently reducing the expression of MTFR2, as well as inhibiting the proliferation, migration and invasion capabilities of OSCC cells and the EMT process. Consequently, AS/BJO-NEs are anticipated to provide novel choices and hope for the clinical therapy of OSCC.

## Supporting information

S1 File Raw-images. (PDF)

S2 File Multivariate Cox analysis of 46 genes. (ZIP)

S1 Table Information of Lentiviral Vectors. (PDF)

S2 Table Benjiammini-Hochberg correction of 49 genes. (XLSX)

S3 Table Significant genes after multivariate Cox analysis. (XLSX)

S4 Table GSEA enrichment analysis for EMT binary classification-related DEGs. (XLS)

S5 Table DEGs between the OSCC group and the normal group in TCGA-OSCC dataset. (XLS)

S6 Table DEGs between the OSCC group and the normal group in GSE37991 dataset. (XLS)

S7 Table MMPBSA binding energy results. (PDF)

S1 Fig Clustering analysis of TCGA-OSCC samples based on EMT prognosis-related genes. (PDF)

S2 Fig Construction and analysis of the TCGA-OSCC gene co-expression network to identify modules most relevant to selected subtypes. (TIF)

S3 Fig Single-gene analysis of MTFR2, screening the core target regulated by the AS/BJO-NEs on MTFR2. (TIF)

## Abbreviations

OSCC: Oral squamous cell carcinoma
EMT: Epithelial-mesenchymal transition
BJO: Brucea javanica oil
AS-IV: Astragaloside IV
AS/BJO-NEs: Astragaloside-Brucea Javanica Oil nanoemulsions
MTFR2: Mitochondrial Fission Regulator 2
CDK1: Cyclin-dependent kinase 1
WGCNA: weighted gene co-expression network analysis
GO: Gene Ontology
KEGG: Kyoto Encyclopedia of Genes and Genomes
GSEA: Gene Set Enrichment Analysis
MDS: molecular dynamics simulation
DSS: disease-specific survival
PFI: progression-free interval
DFI: disease-free interval survival
OS: overall survival
DEGs: differentially expressed genes
RMSD: Root Mean Square Deviation
RMSF: Root Mean Square Fluctuation
RG: Radius of Gyration
MMPBSA: Molecular Mechanics/Poisson-Boltzmann Surface Area
CCK-8: Cell counting kit-8
PBS: phosphate-buffer solution
AUC: area under the curve.
