# Supplementary material for: Mechanism of Astragaloside-Brucea javanica oil nanoemulsion against oral squamous cell carcinoma through CDK1/MTFR2: Network pharmacology, bioinformatics, and experimental studies
Source: PLoS One. 2025 Aug 1;20(8):e0329622. doi: 10.1371/journal.pone.0329622 (PMC12316279; doi:10.1371/journal.pone.0329622)
Supplement: S1 File — (PDF) [file pone.0329622.s001.pdf]

Representative image

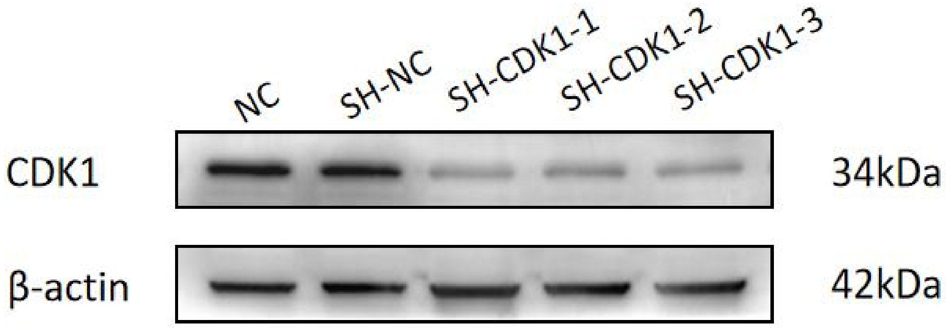

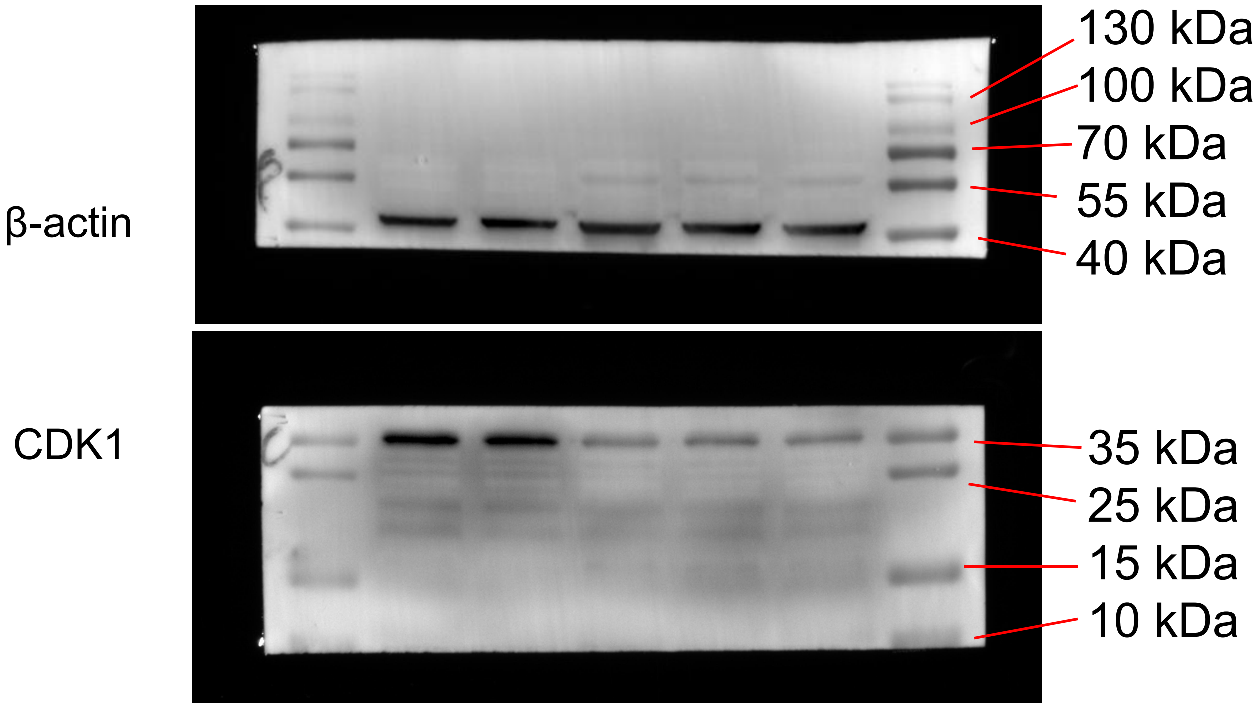

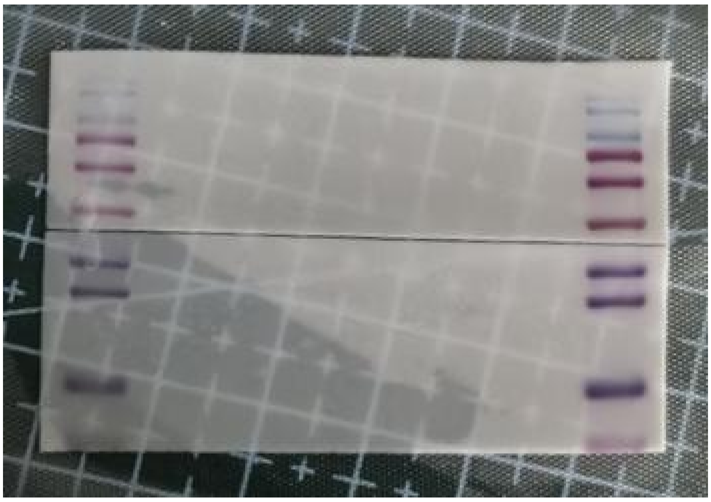

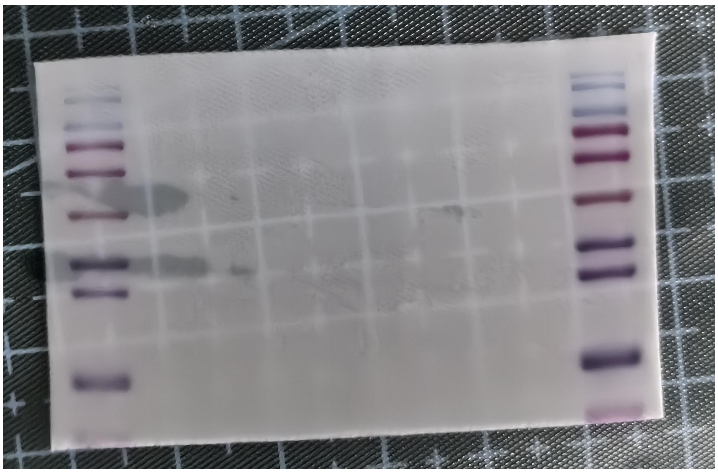

Repeat 2

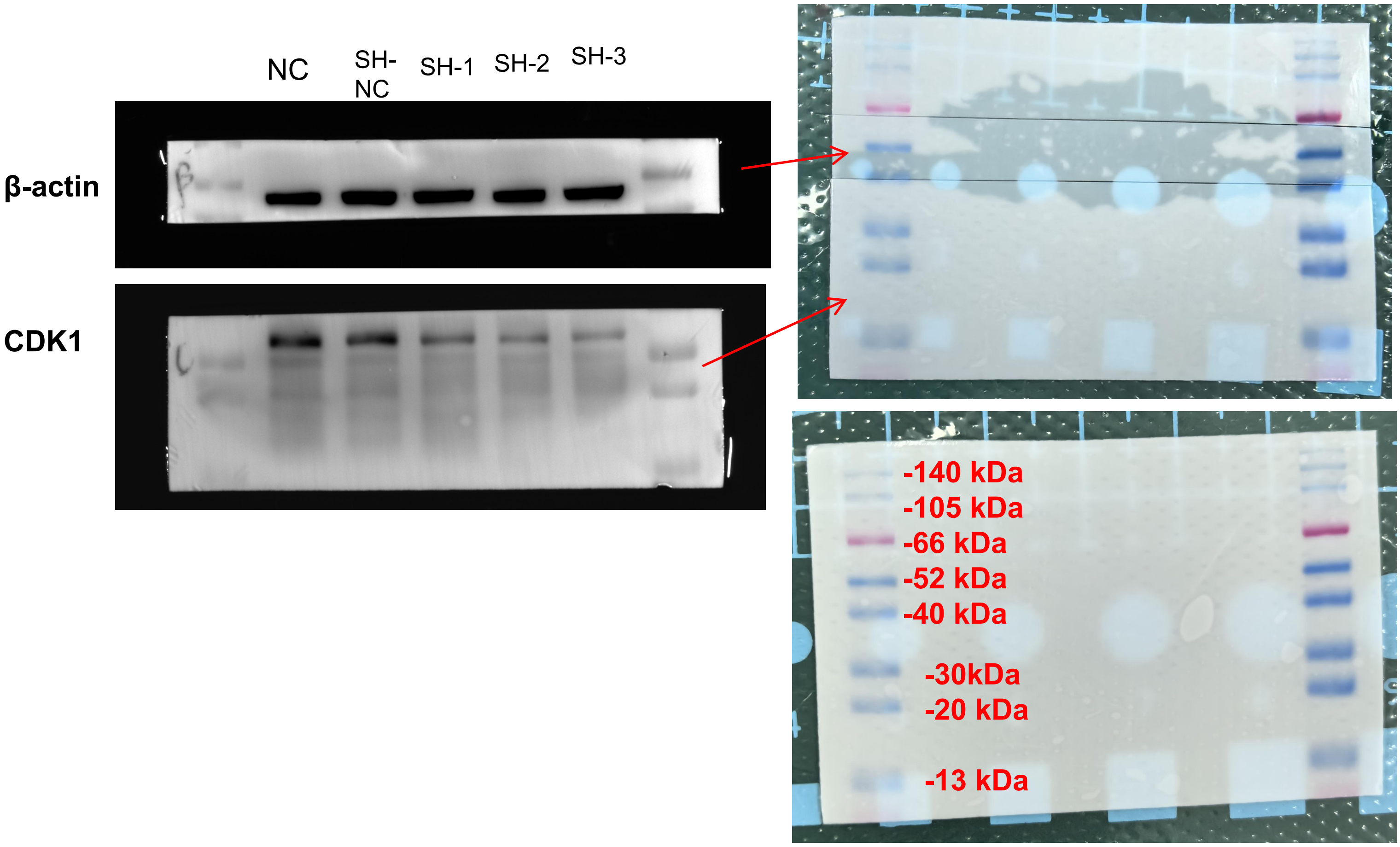

Repeat 3

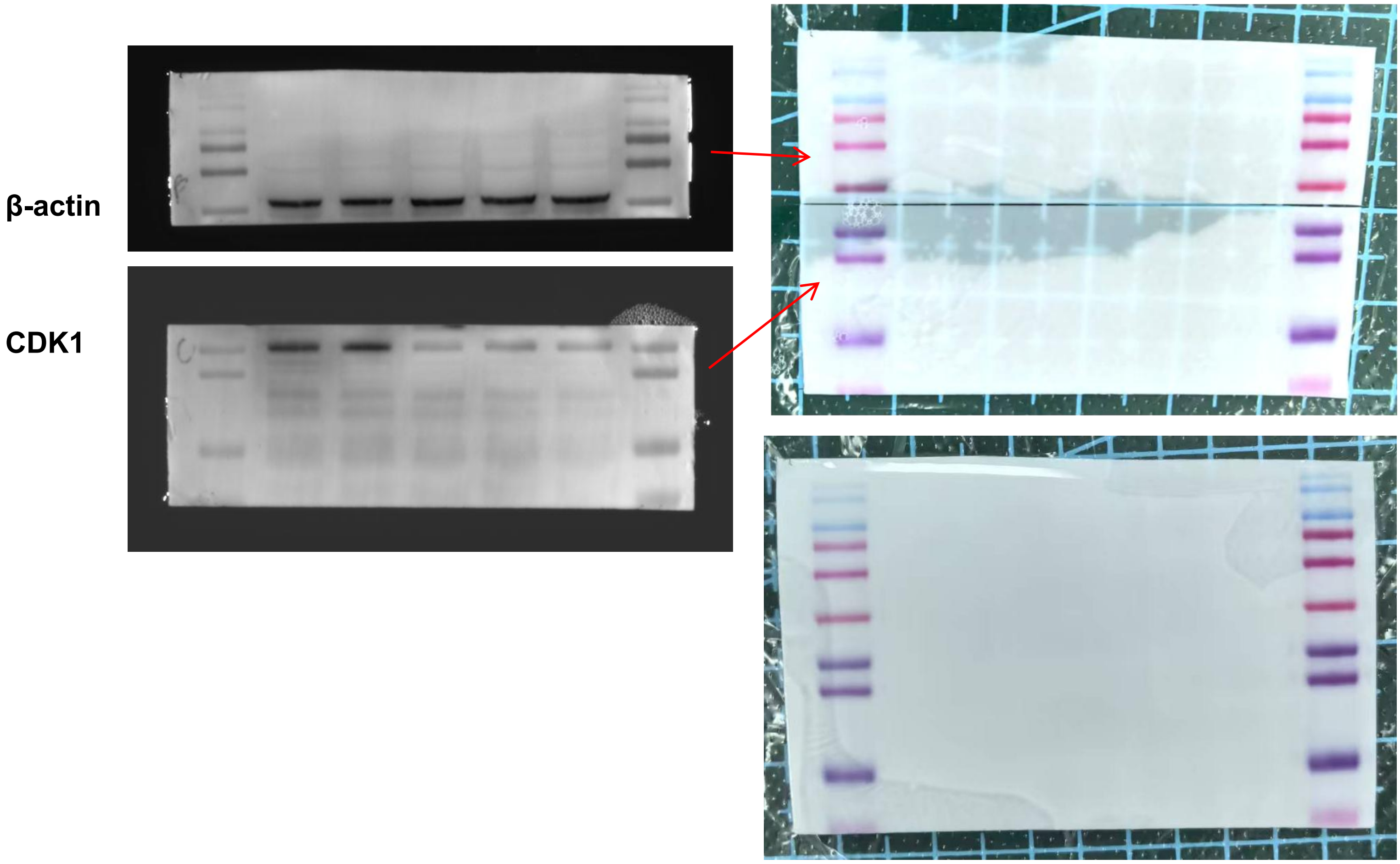

Representative image

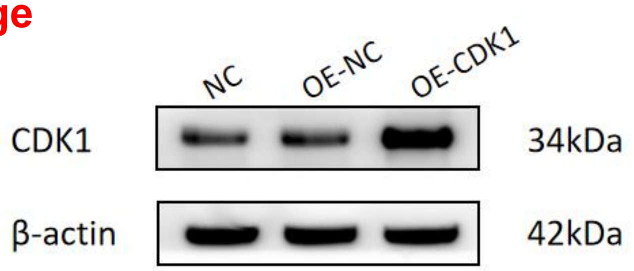

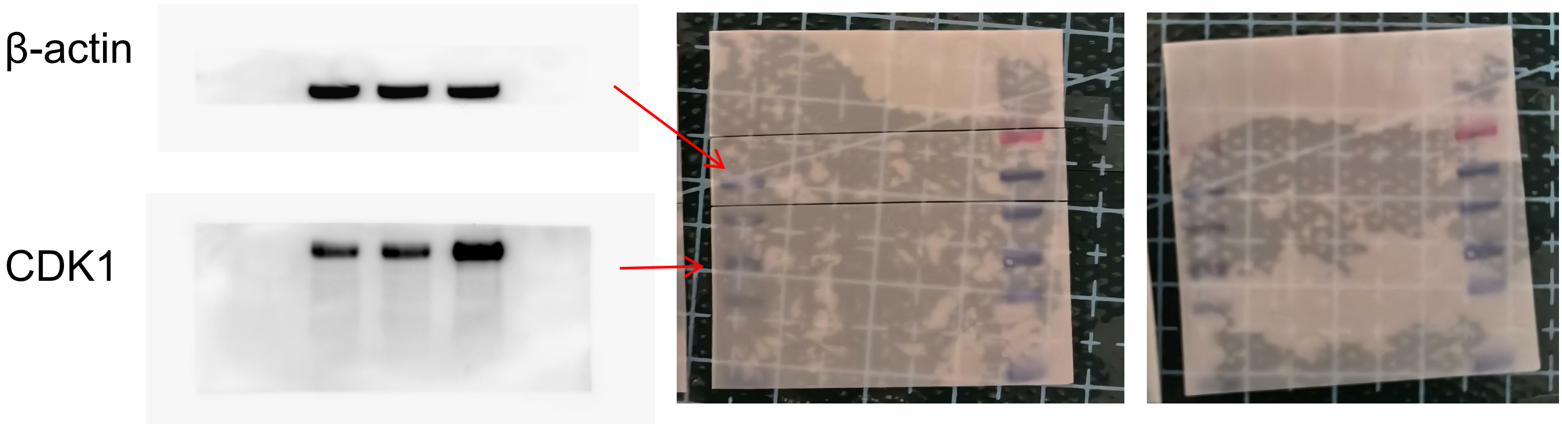

Repeat 2

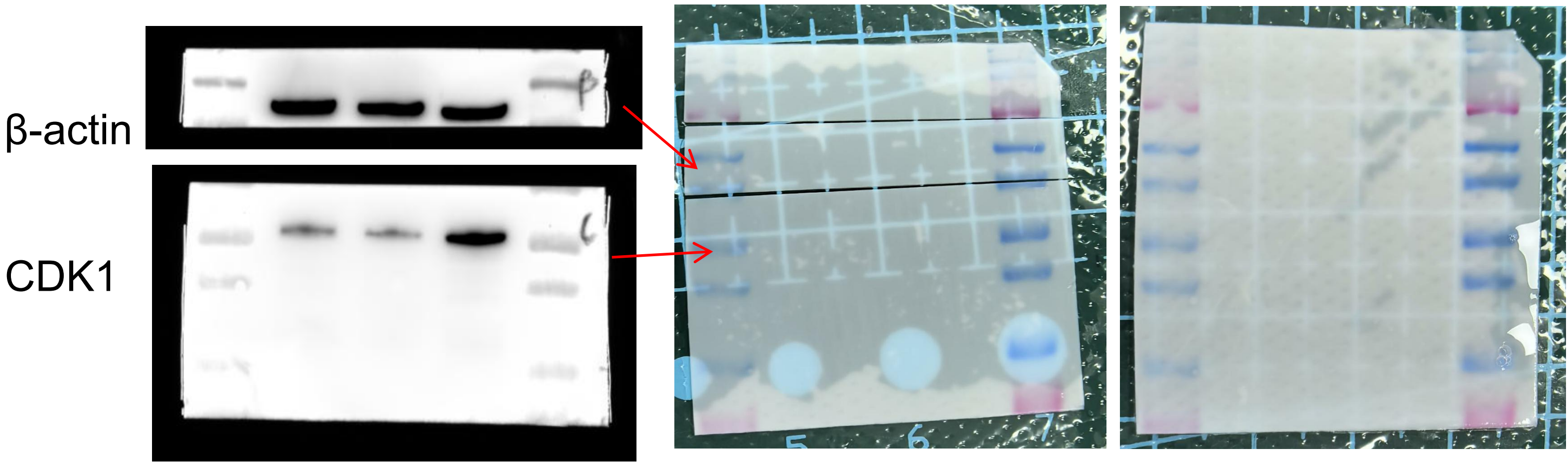

Repeat 3

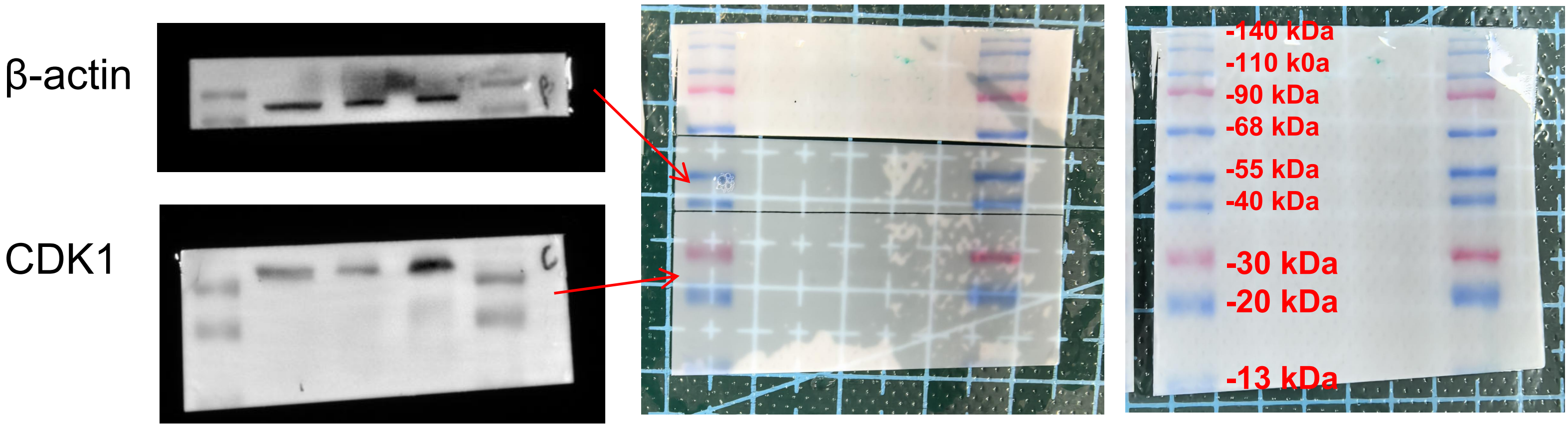

Representative image

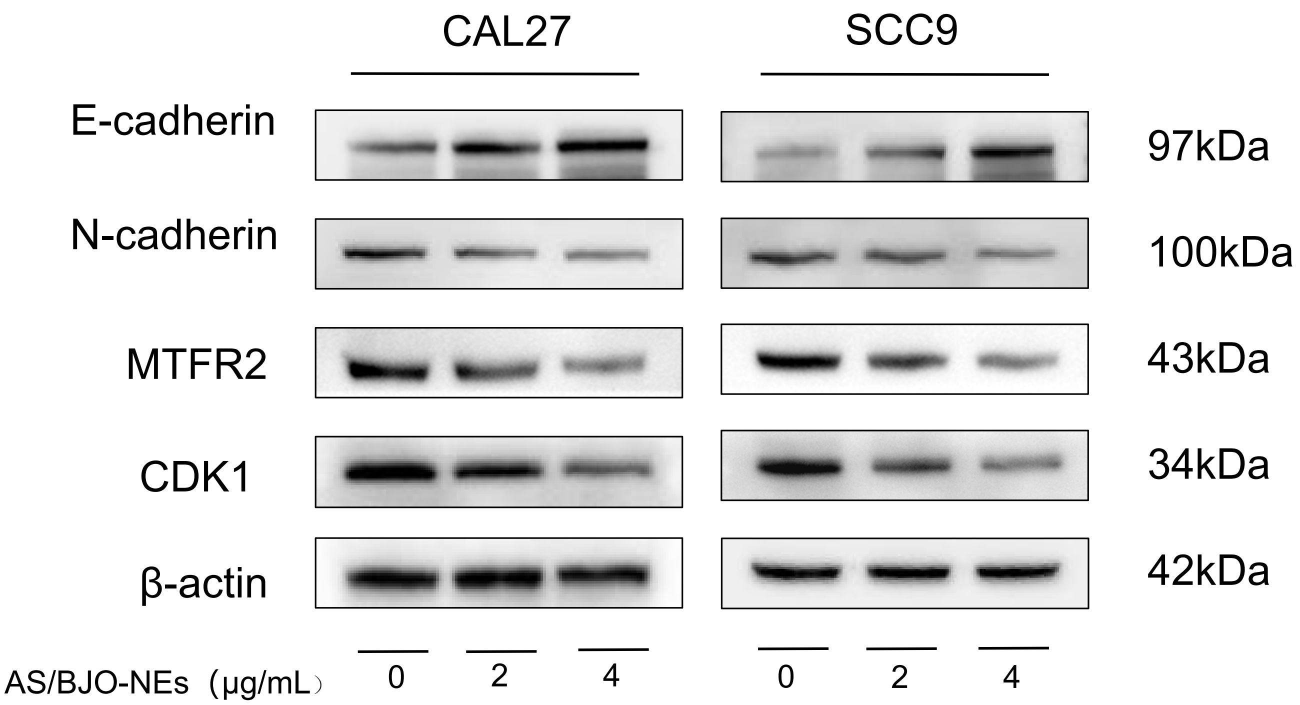

CAL27（Representative image）

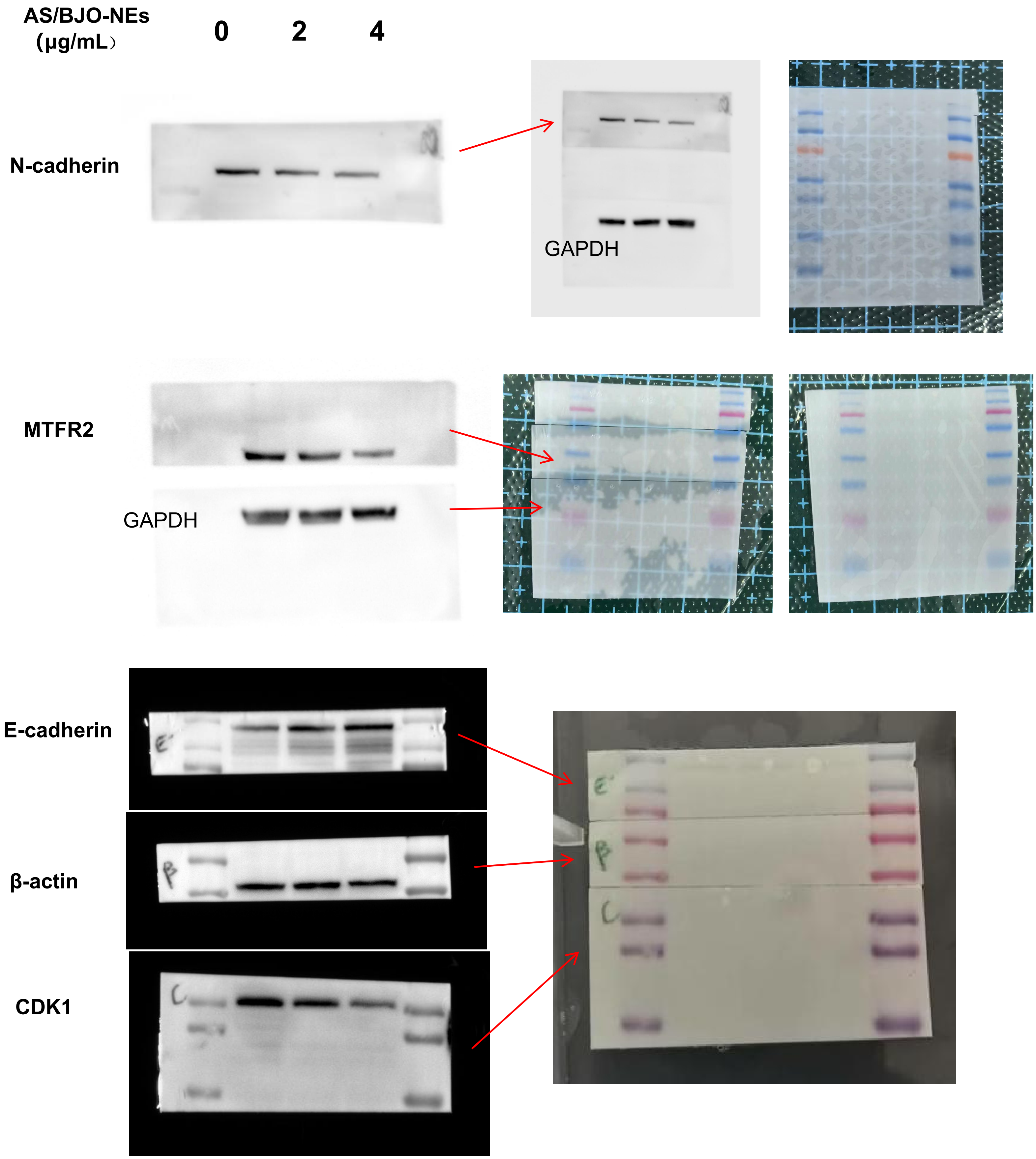

CAL27（Repeat 2）

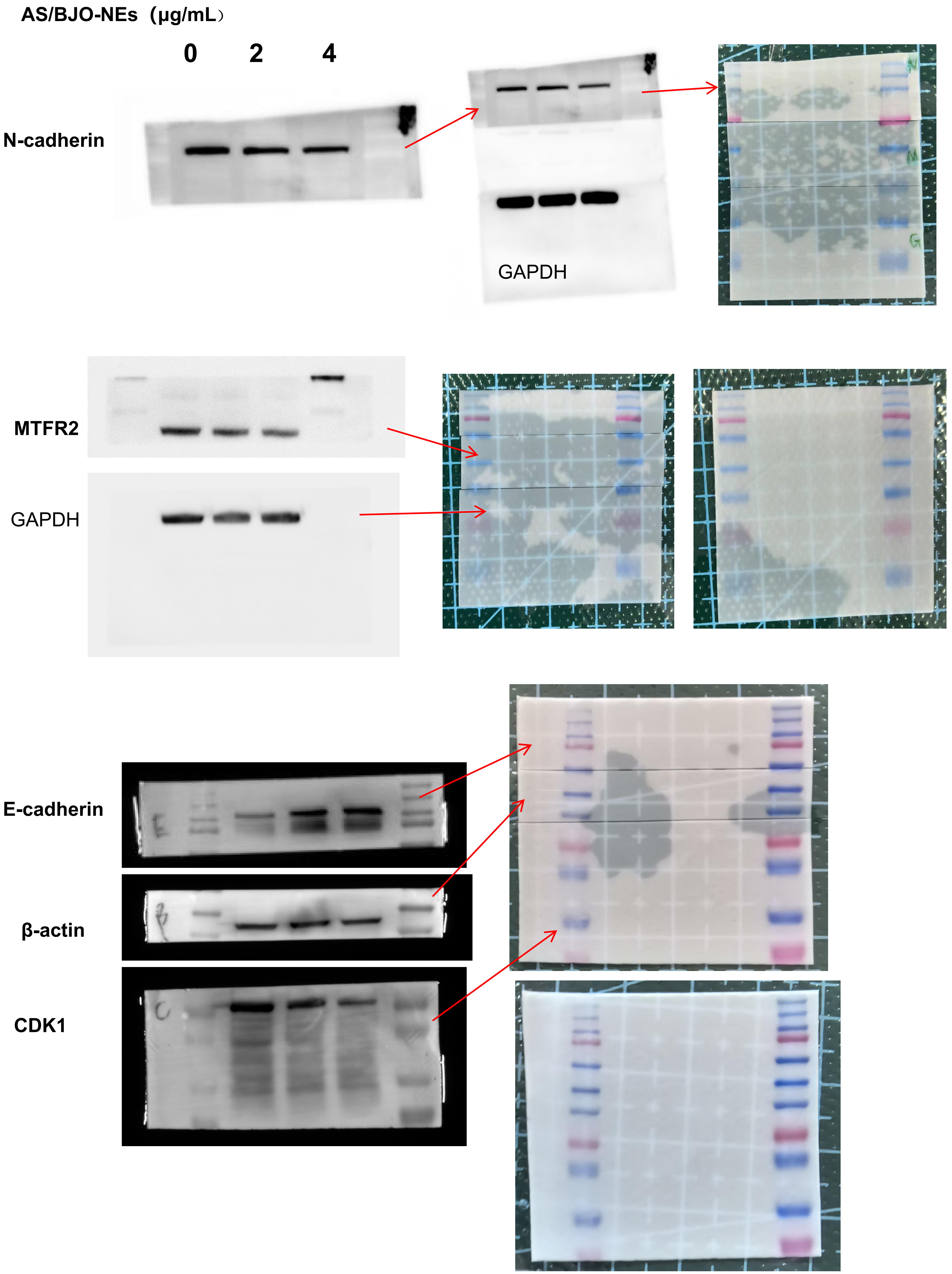

CAL27（Repeat 3）

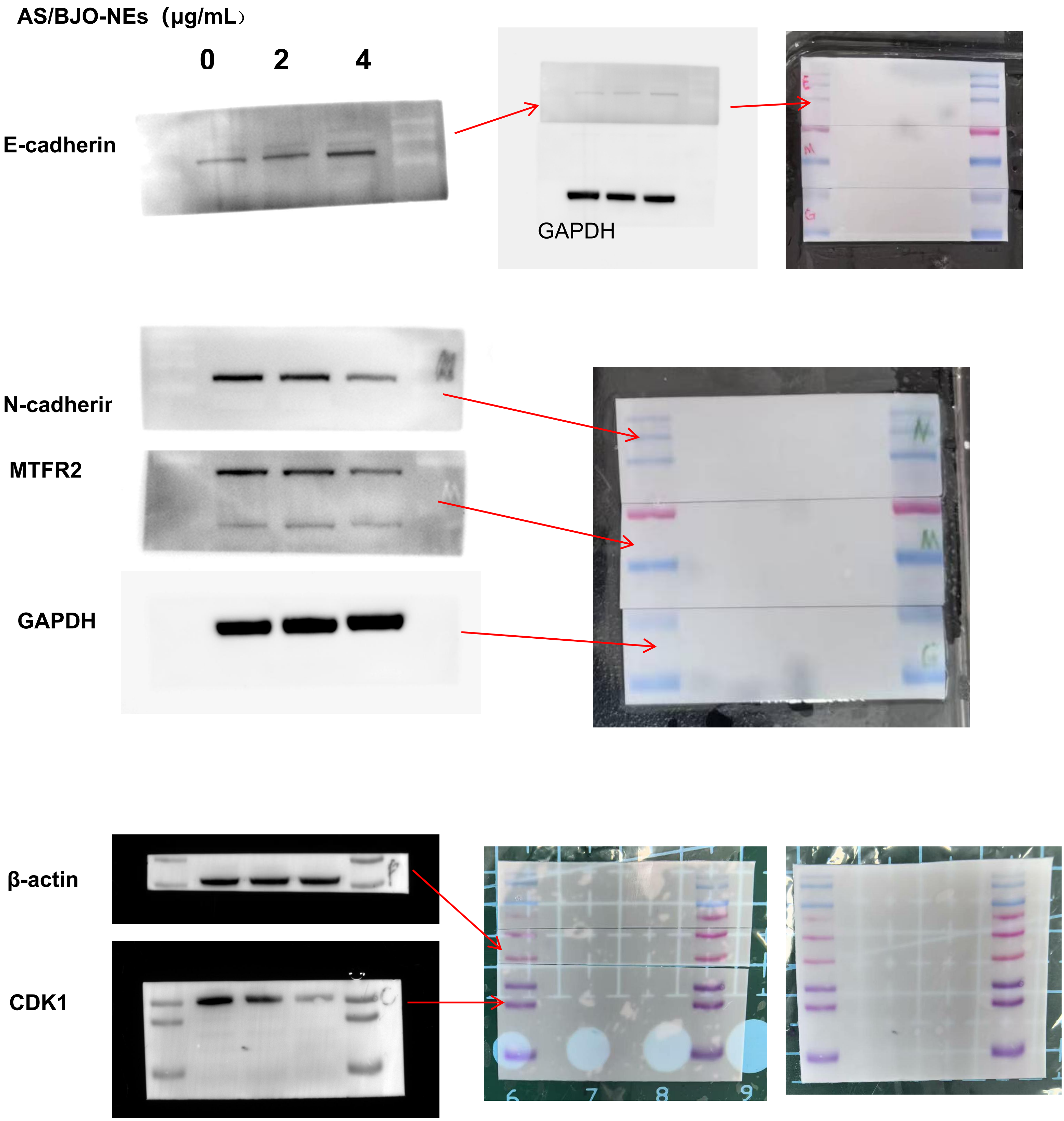

SCC9 (Representative image)

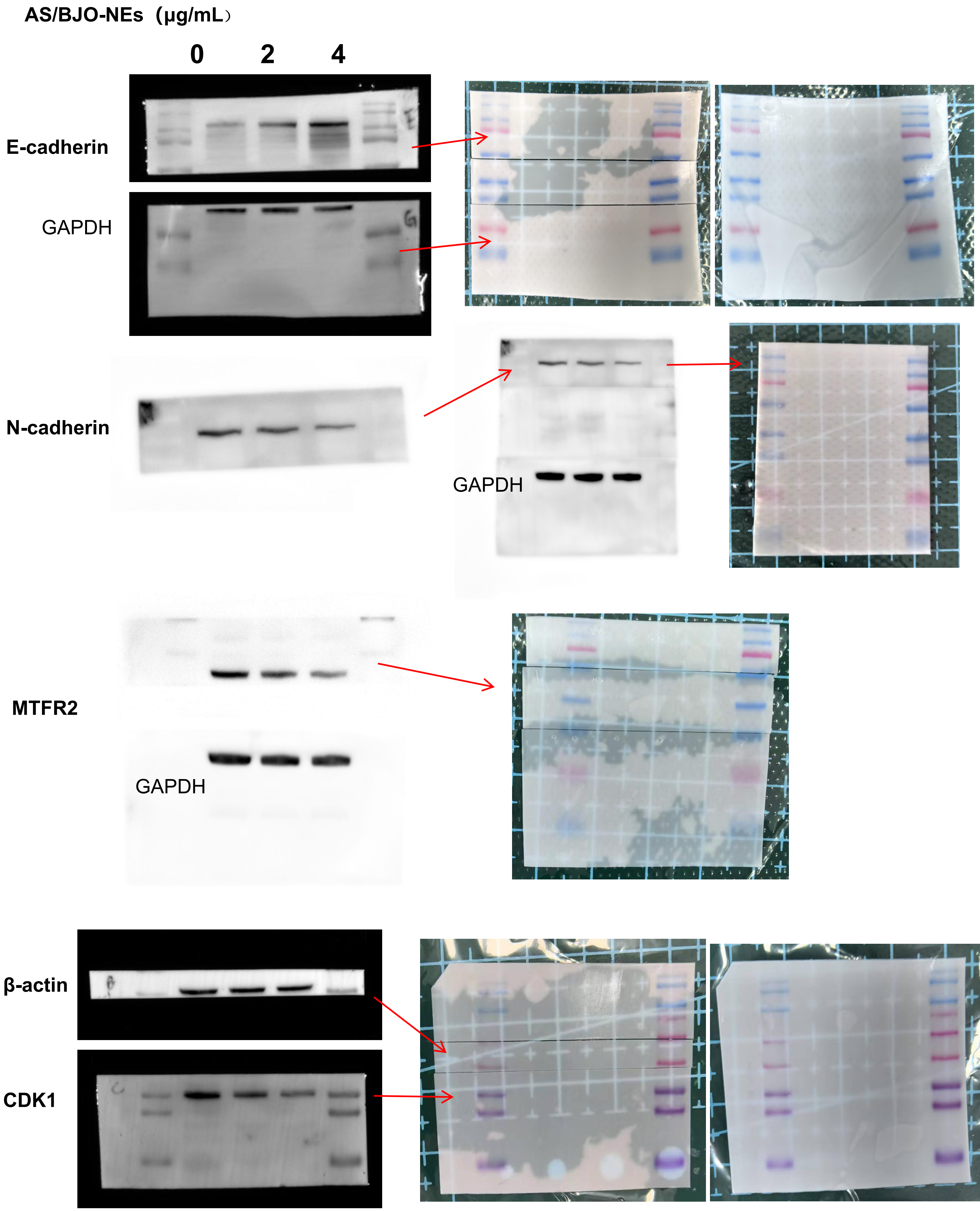

SCC9 Repeat 2

AS/BJO-NEs (μg/mL)

0 2 4

E-cadherin

MTFR2

GAPDH

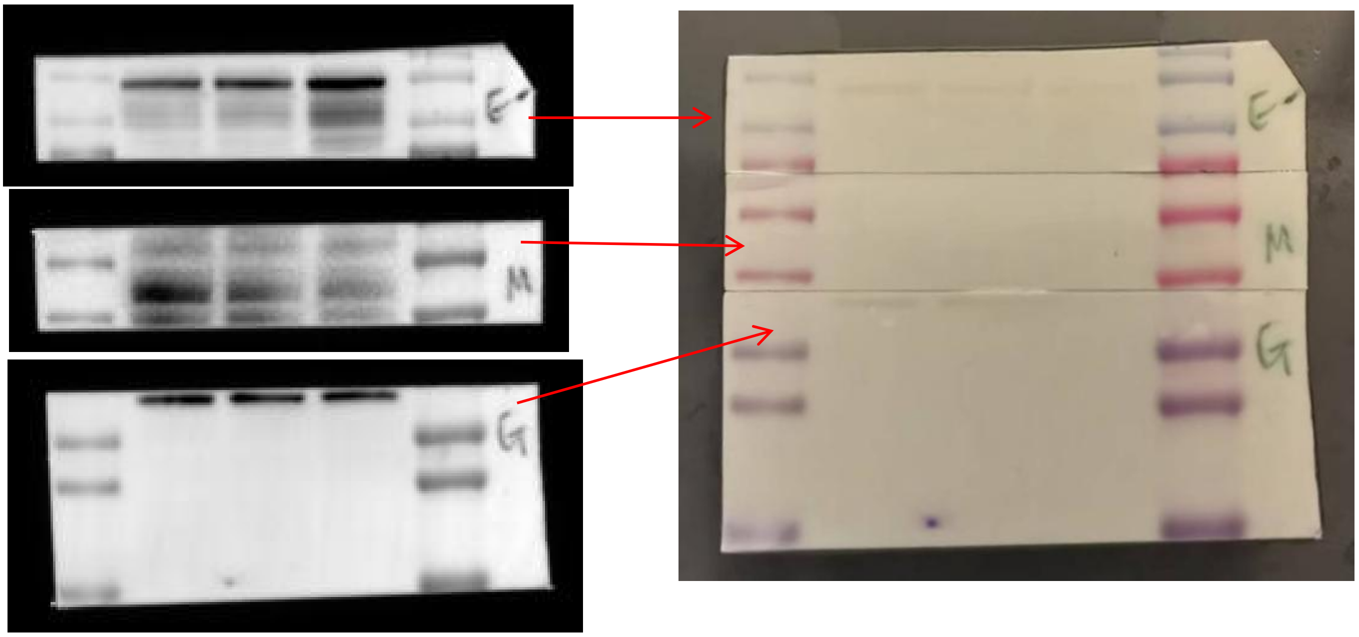

N-cadherin

β-actin

CDK1

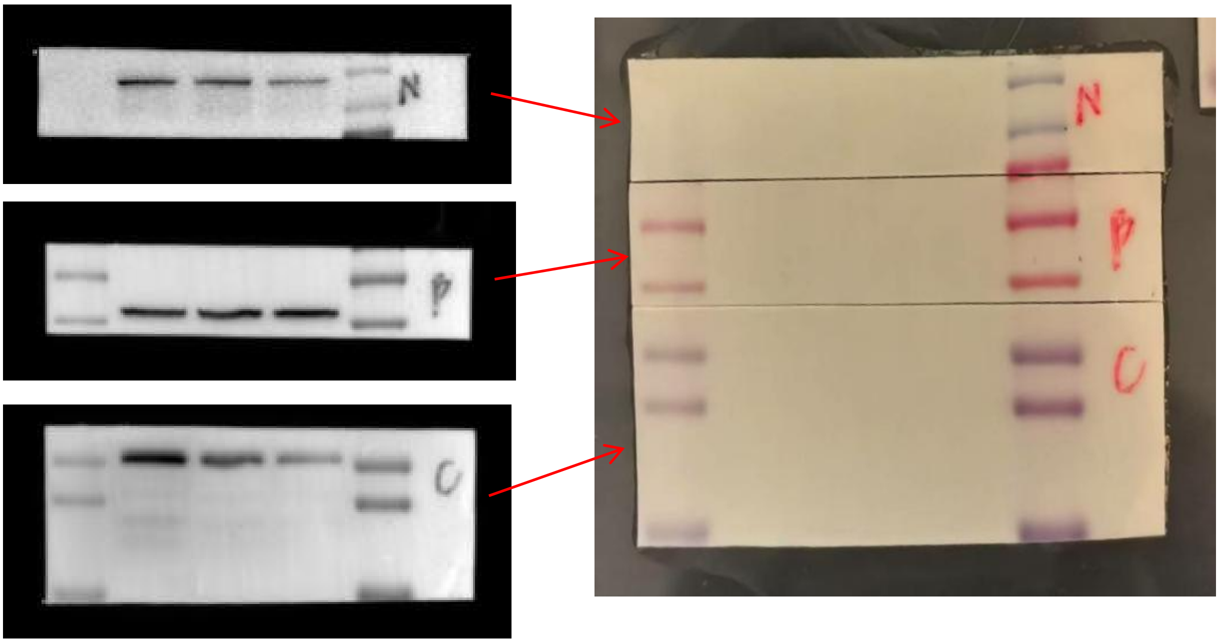

SCC9 Repeat 3

AS/BJO-NEs (μg/mL)

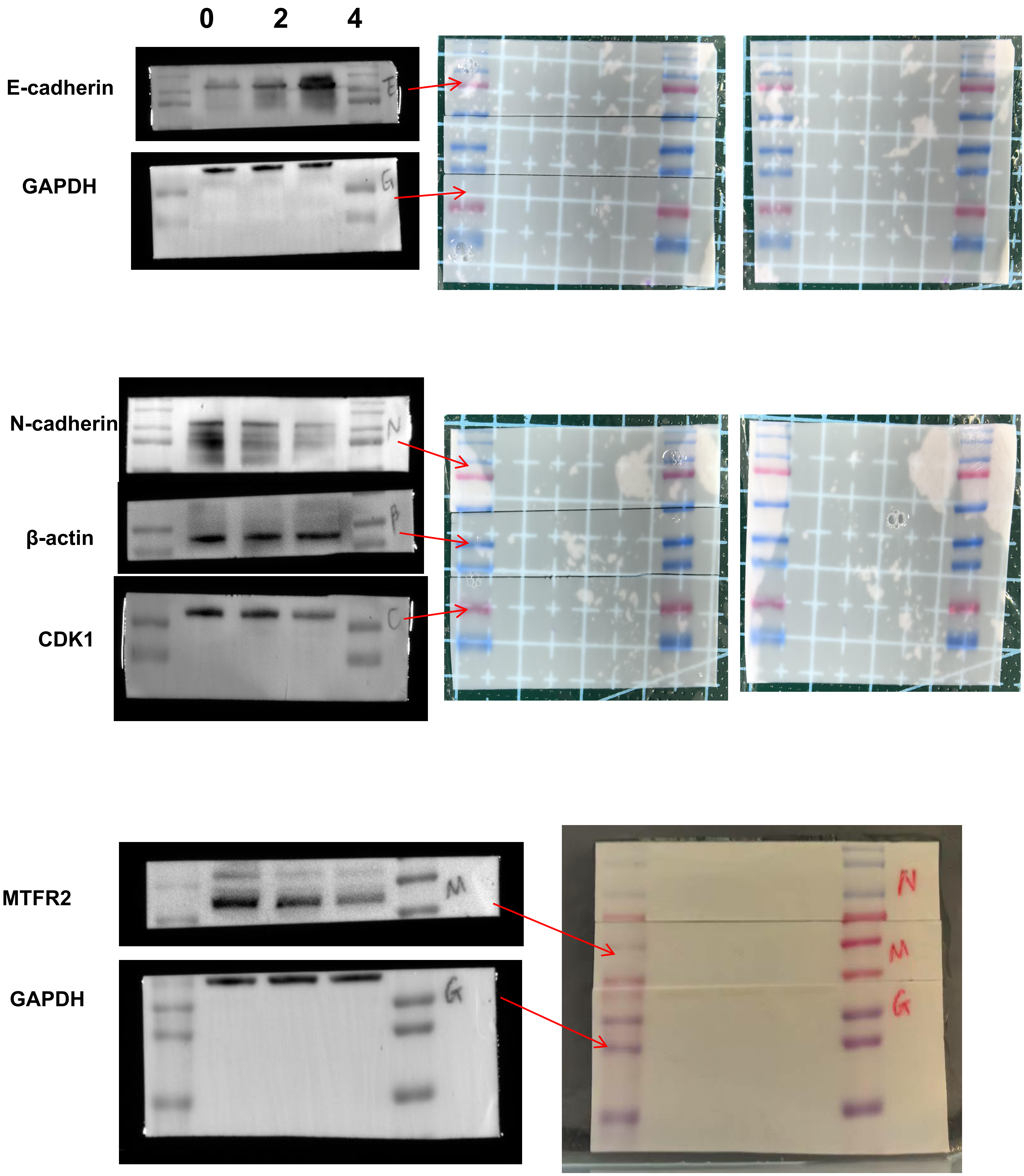

Representative image

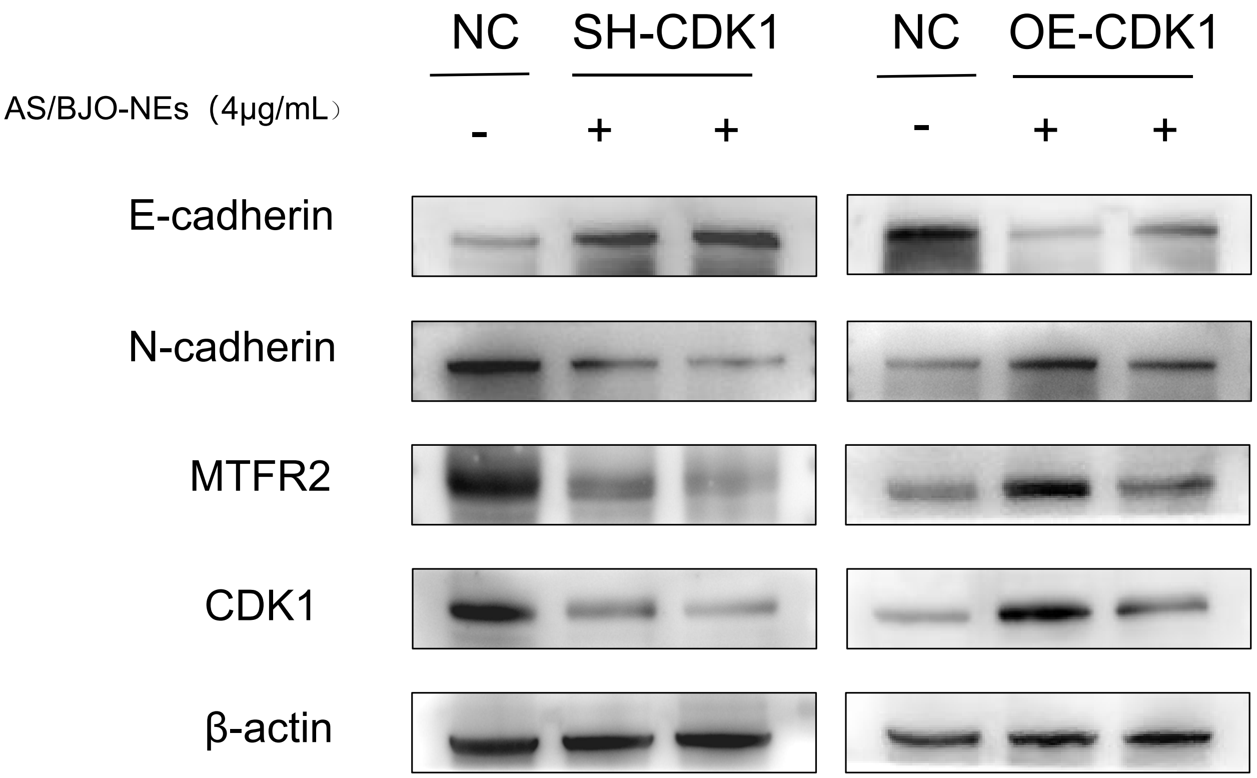

Representative image

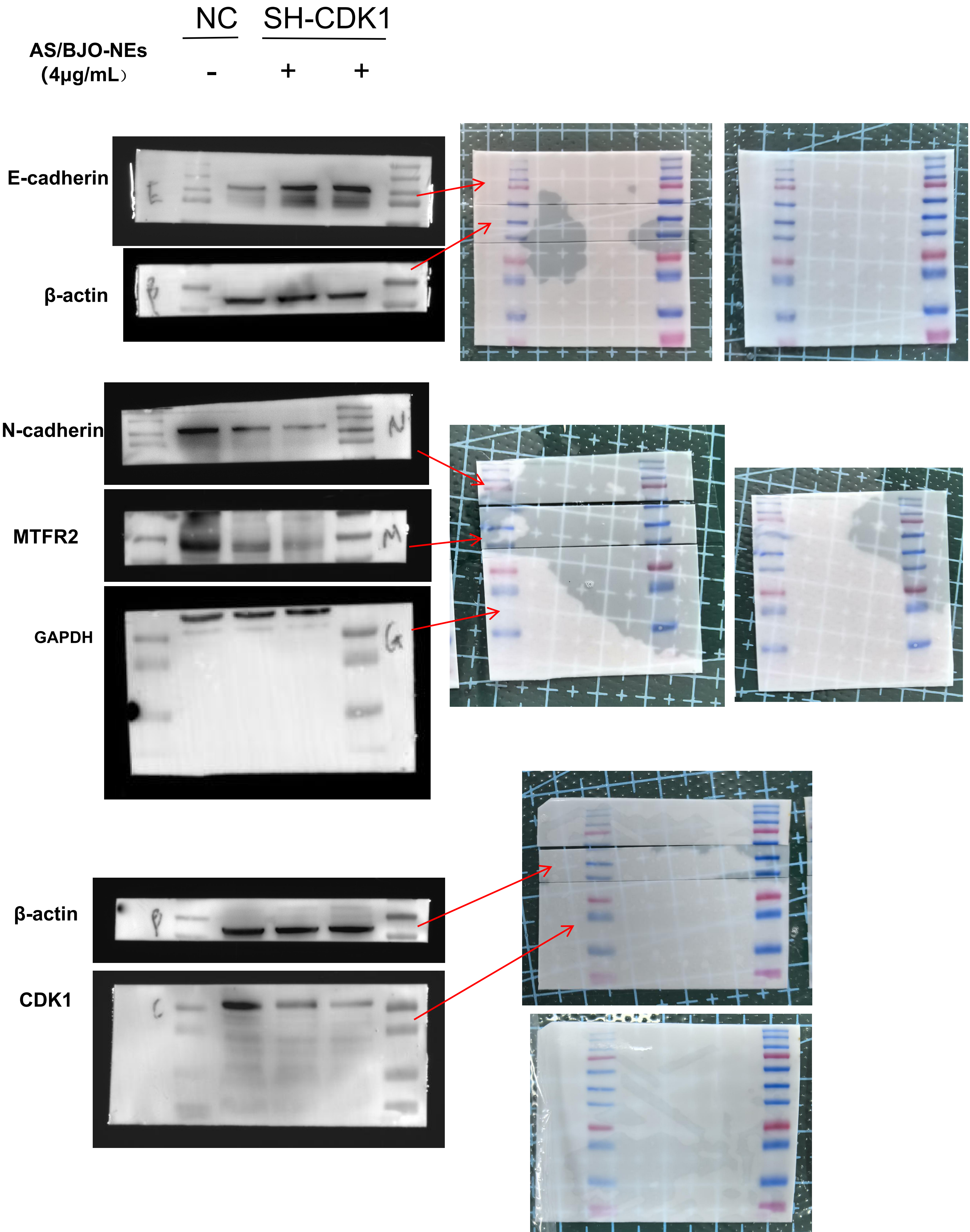

Repeat 2

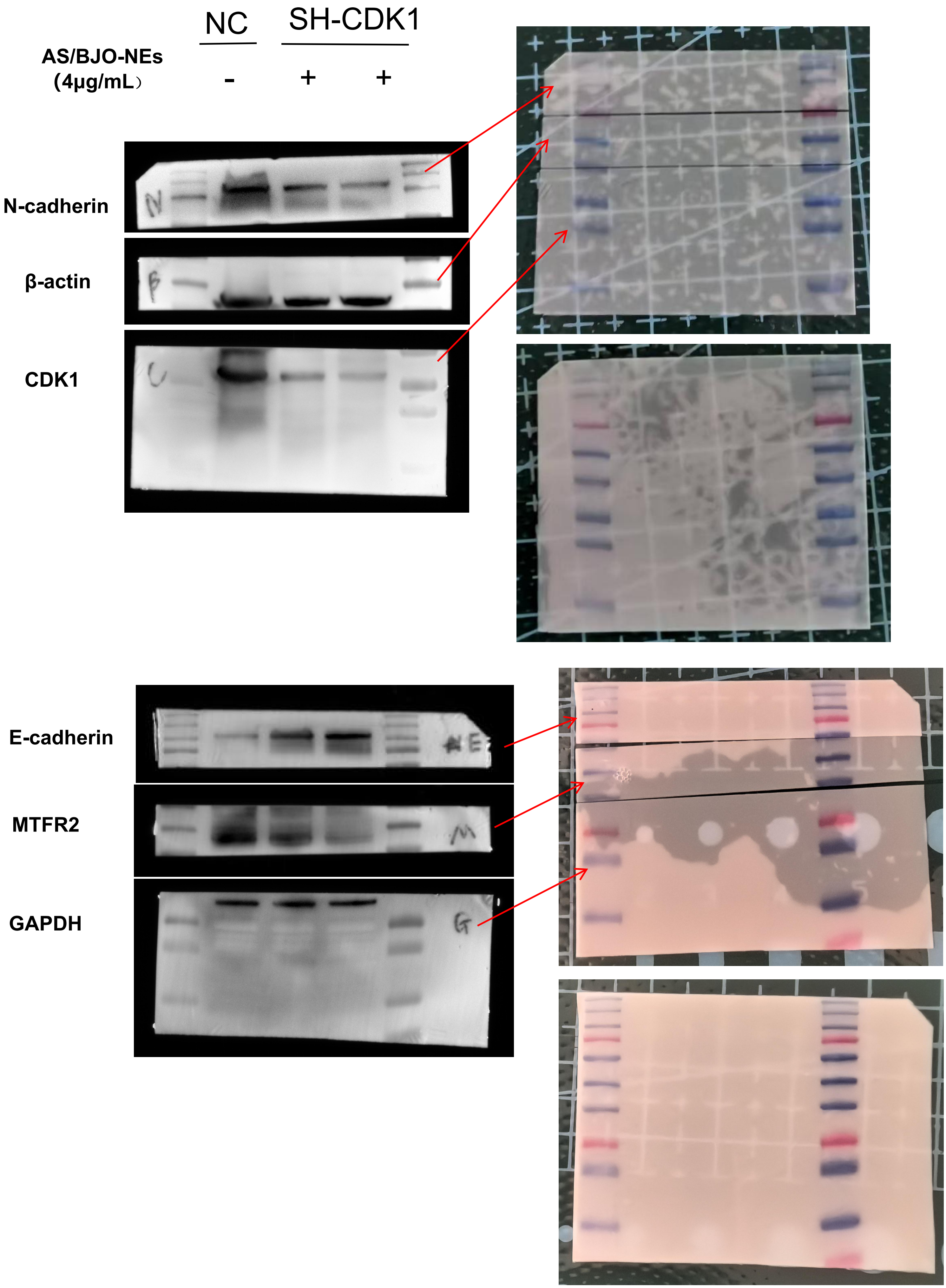
NC
SH-CDK1
AS/BJO-NEs
(4μg/mL)
-
+
+
N-cadherin
β-actin
CDK1
E-cadherin
MTFR2
GAPDH

Repeat 3

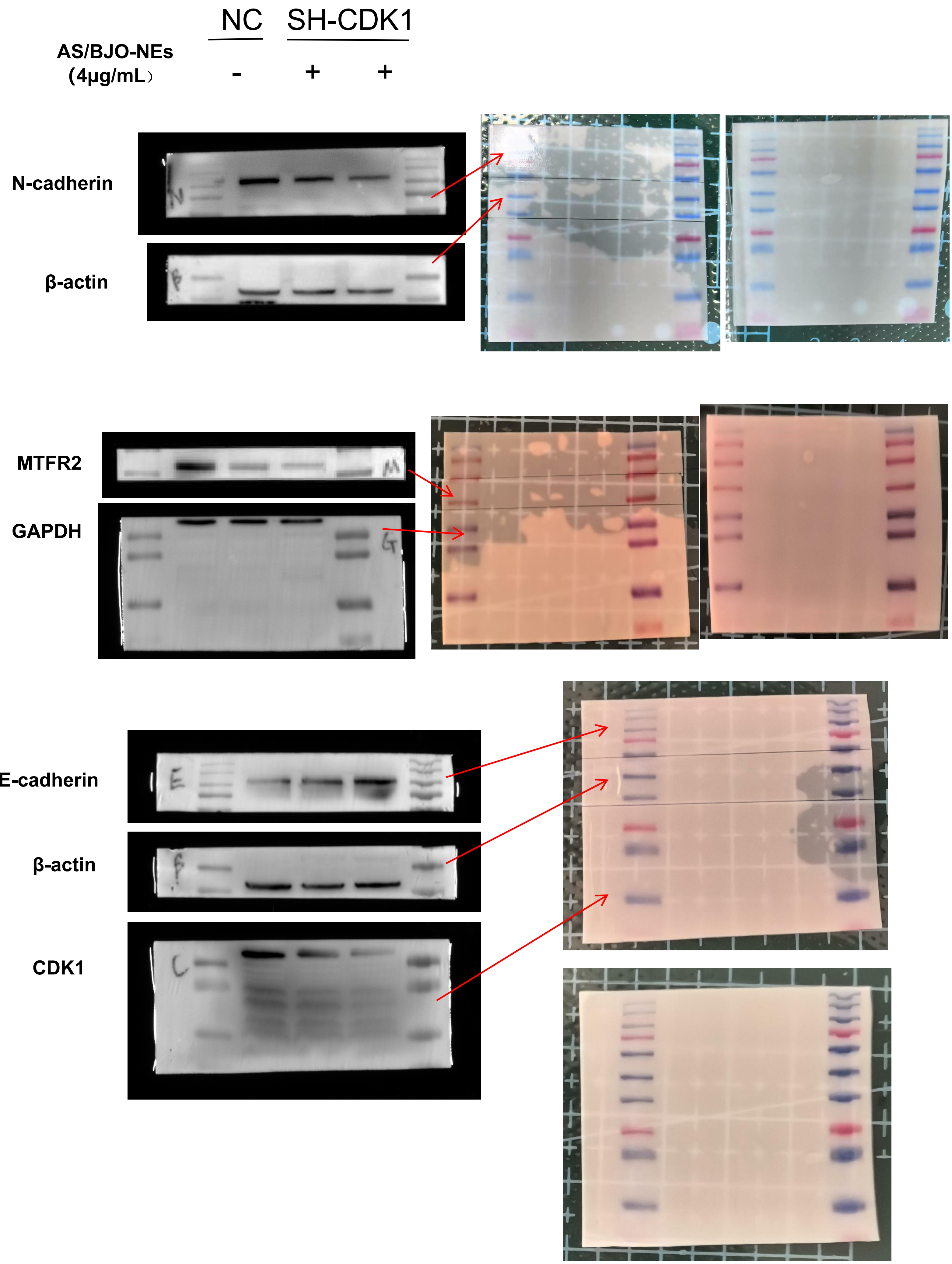
NC
SH-CDK1
AS/BJO-NEs
(4μg/mL)
-
+
+
N-cadherin
β-actin
MTFR2
GAPDH
E-cadherin
β-actin
CDK1

Representative image

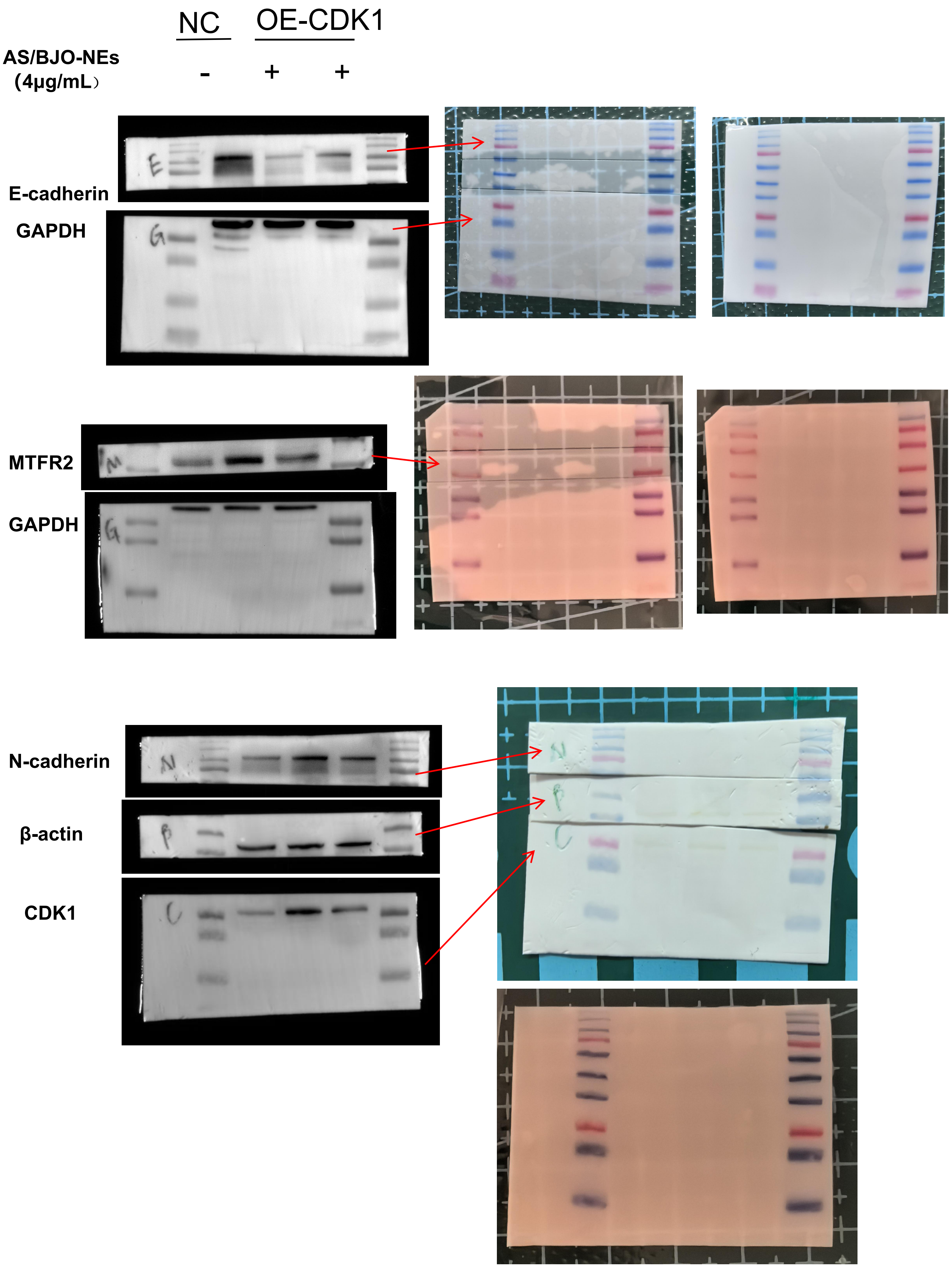

Repeat 2

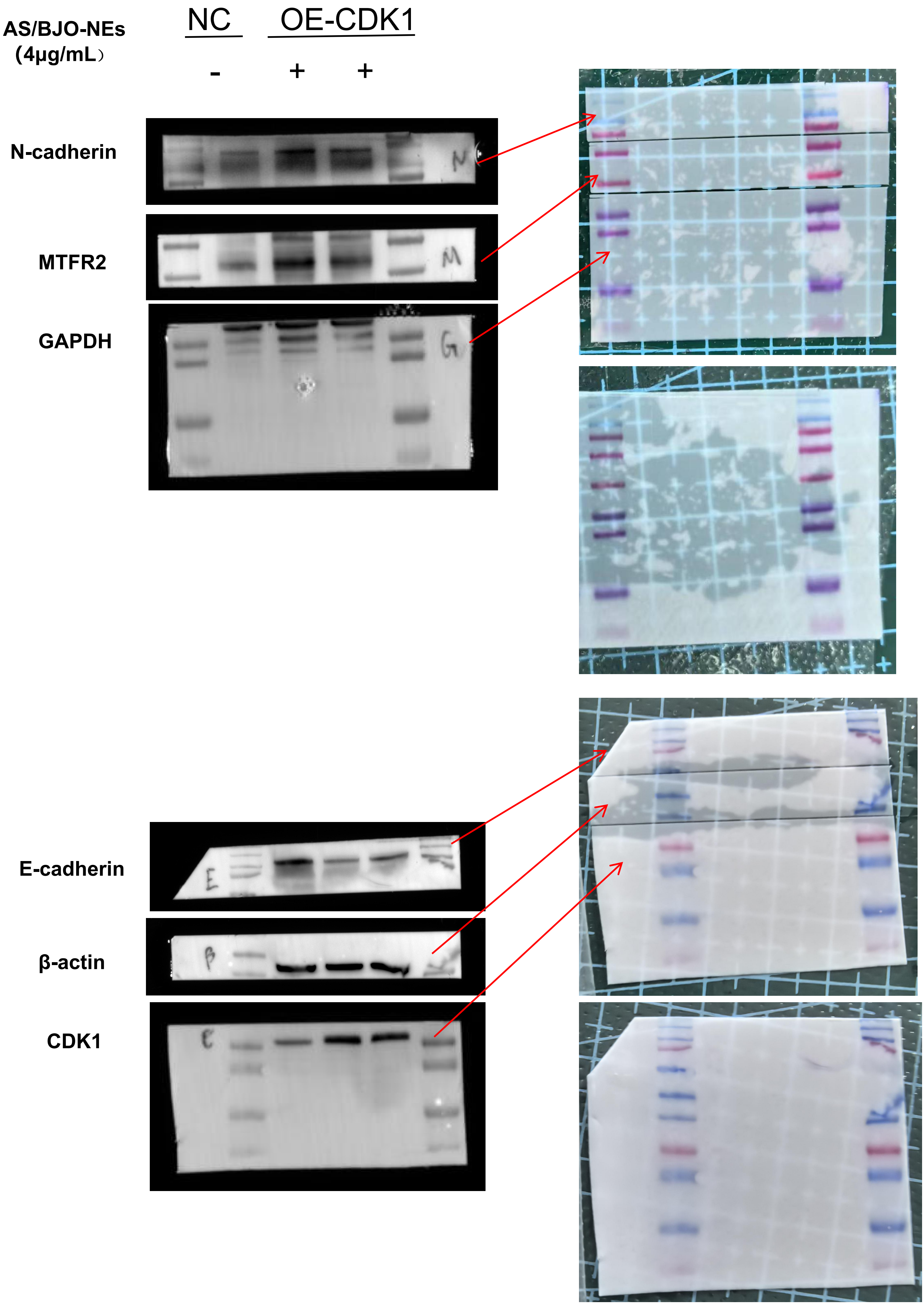
AS/BJO-NEs
(4μg/mL)
NC
OE-CDK1
-
+
+
N-cadherin
MTFR2
GAPDH
E-cadherin
β-actin
CDK1

Repeat 3

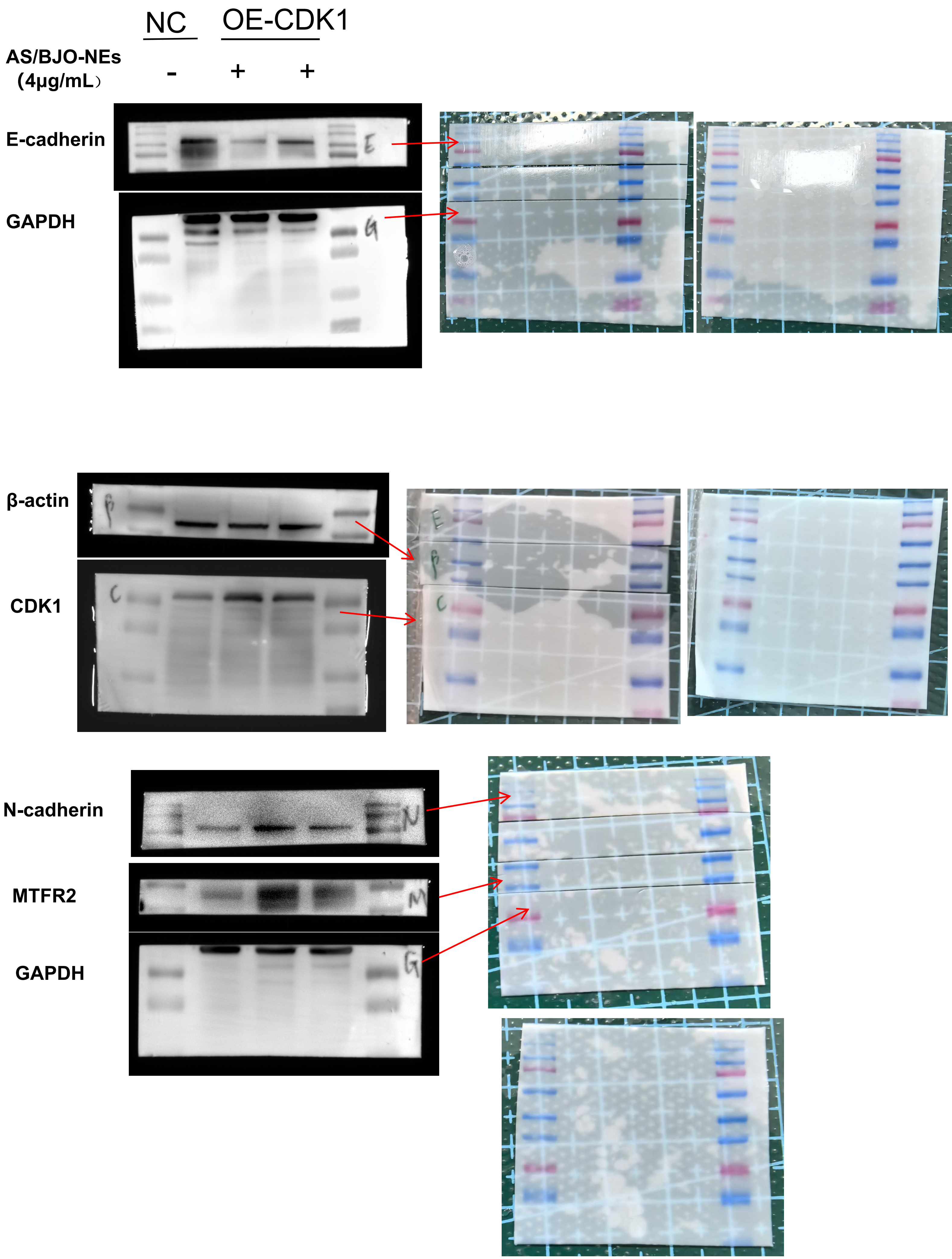

NC
OE-CDK1
AS/BJO-NEs
(4μg/mL)
-
+
+
E-cadherin
GAPDH
β-actin
CDK1
N-cadherin
MTFR2
GAPDH
