# Supplementary figures and images for: Mechanism of Astragaloside-Brucea javanica oil nanoemulsion against oral squamous cell carcinoma through CDK1/MTFR2: Network pharmacology, bioinformatics, and experimental studies
Source: PLoS One. 2025 Aug 1;20(8):e0329622. doi: 10.1371/journal.pone.0329622 (PMC12316279; doi:10.1371/journal.pone.0329622)

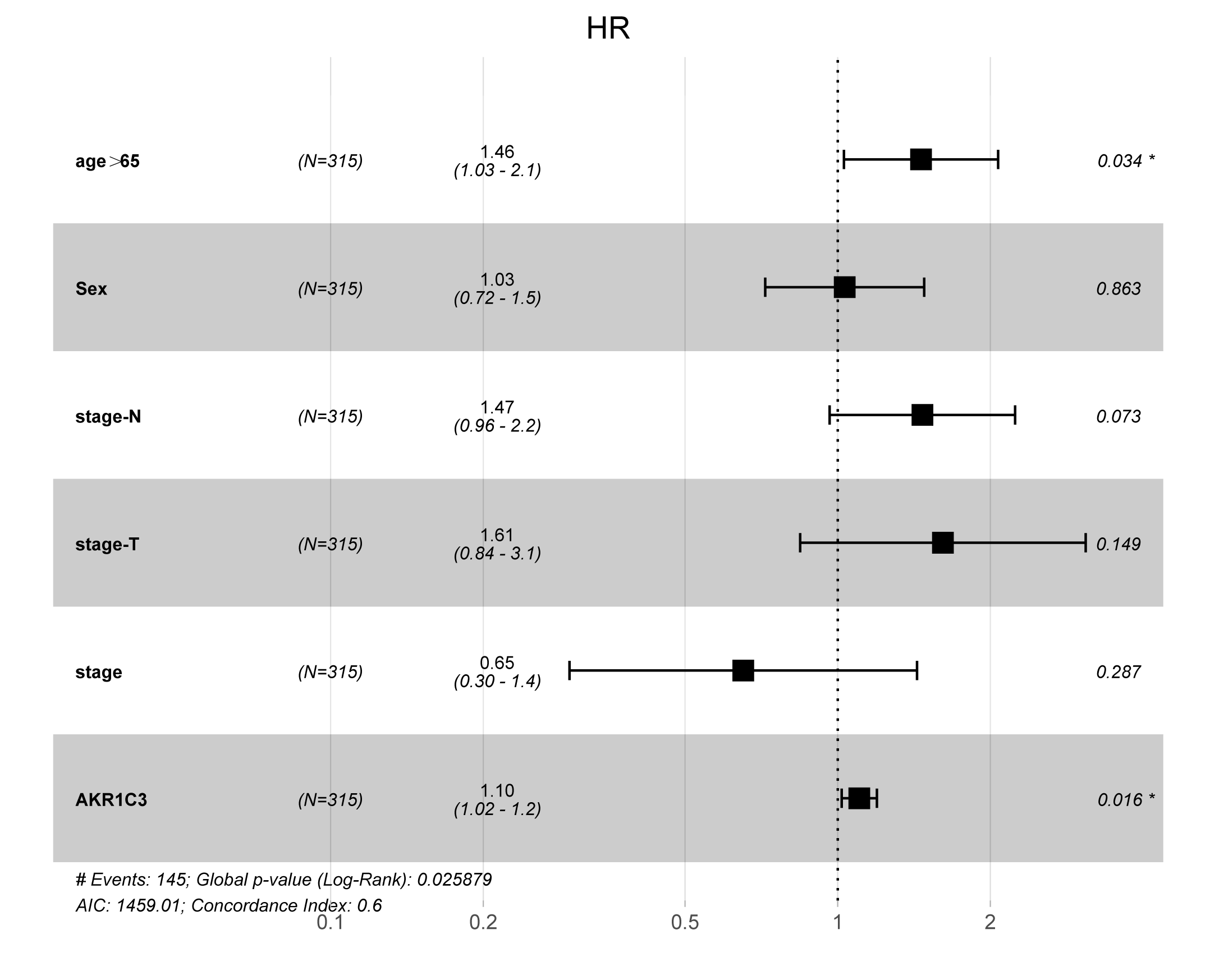

Supplement: S2 File — (ZIP) [file pone.0329622.s002.zip › 多因素Cox分析-46-tiff/AKR1C3.tif]

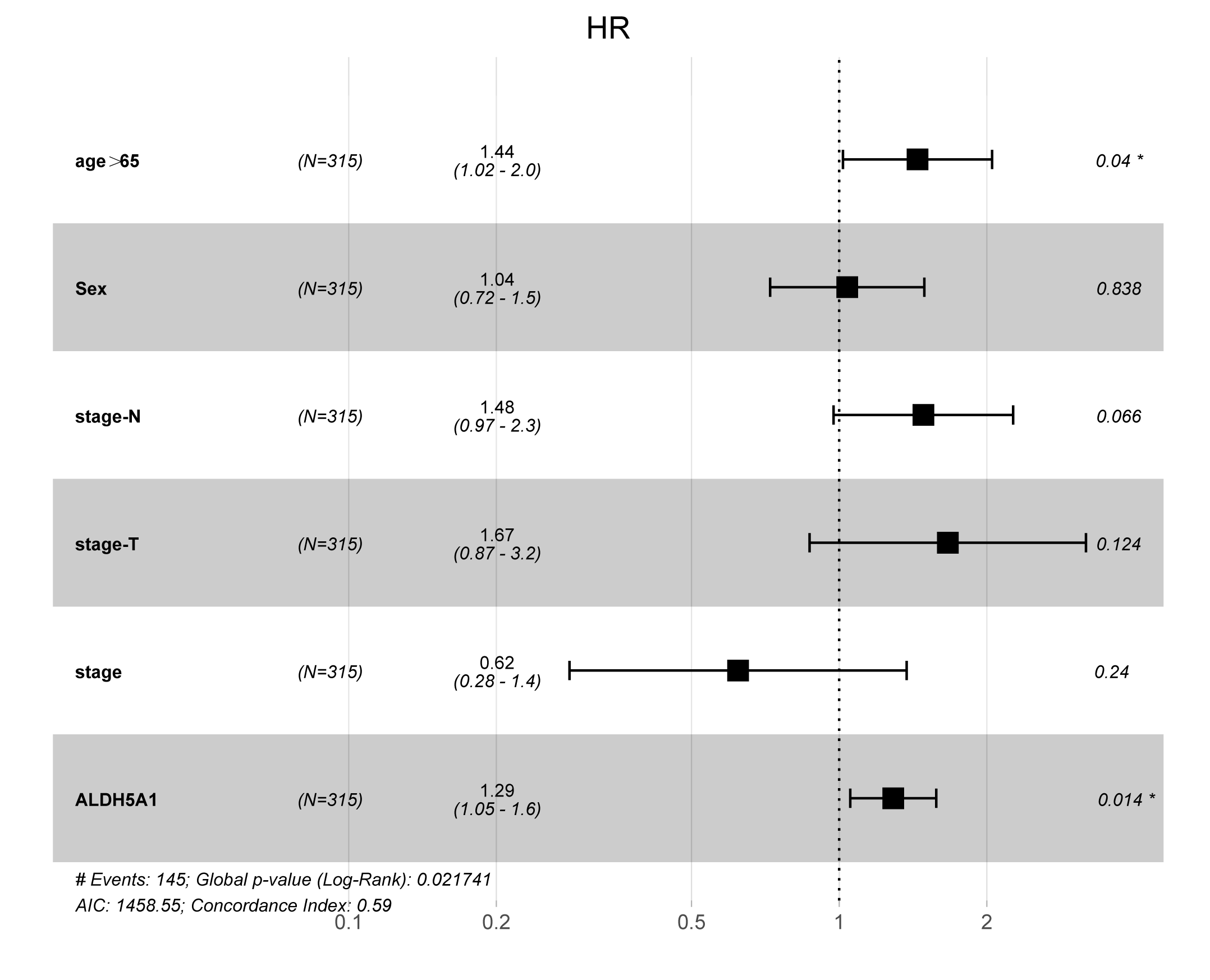

Supplement: S2 File — (ZIP) [file pone.0329622.s002.zip › 多因素Cox分析-46-tiff/ALDH5A1.tif]

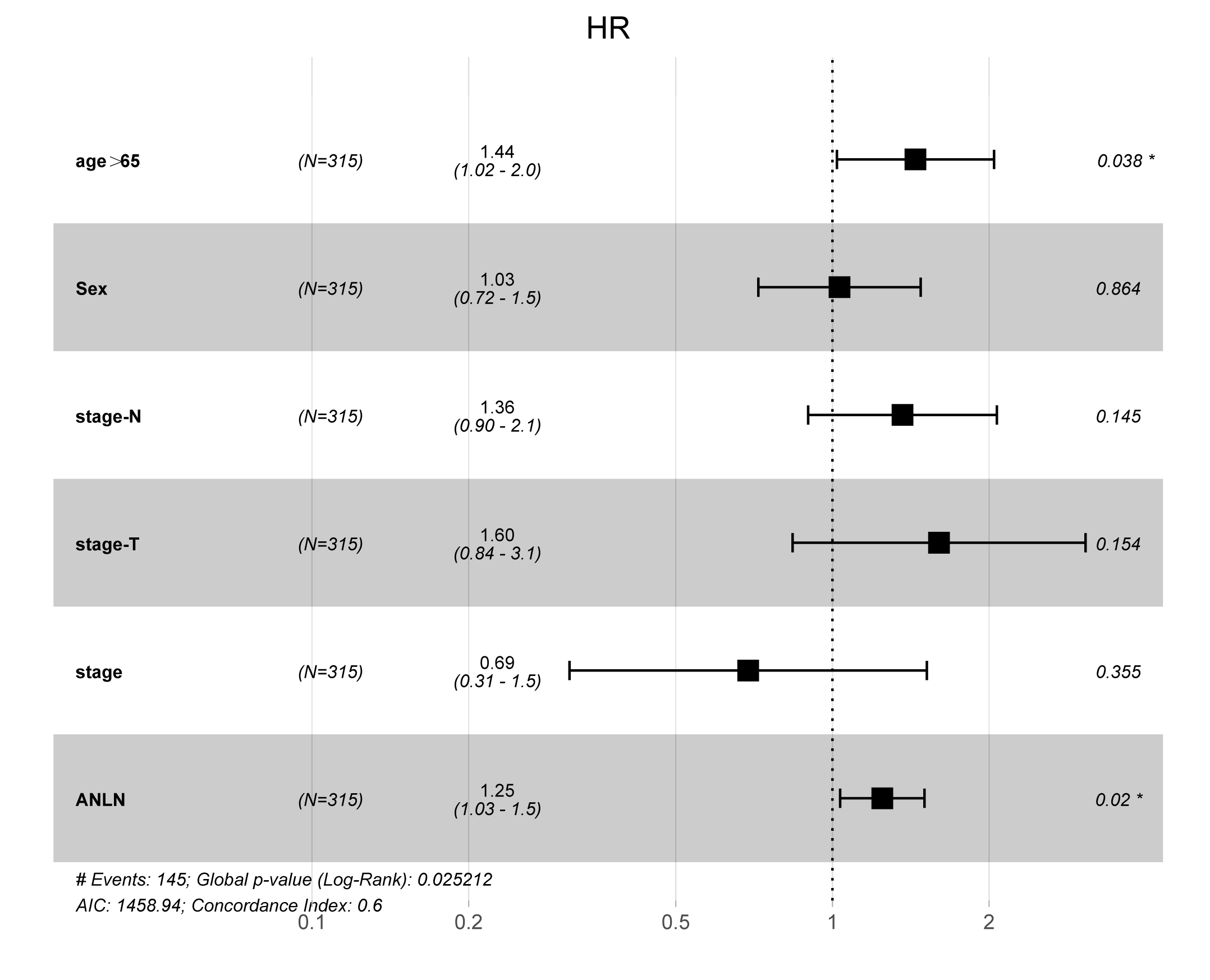

Supplement: S2 File — (ZIP) [file pone.0329622.s002.zip › 多因素Cox分析-46-tiff/ANLN.tif]

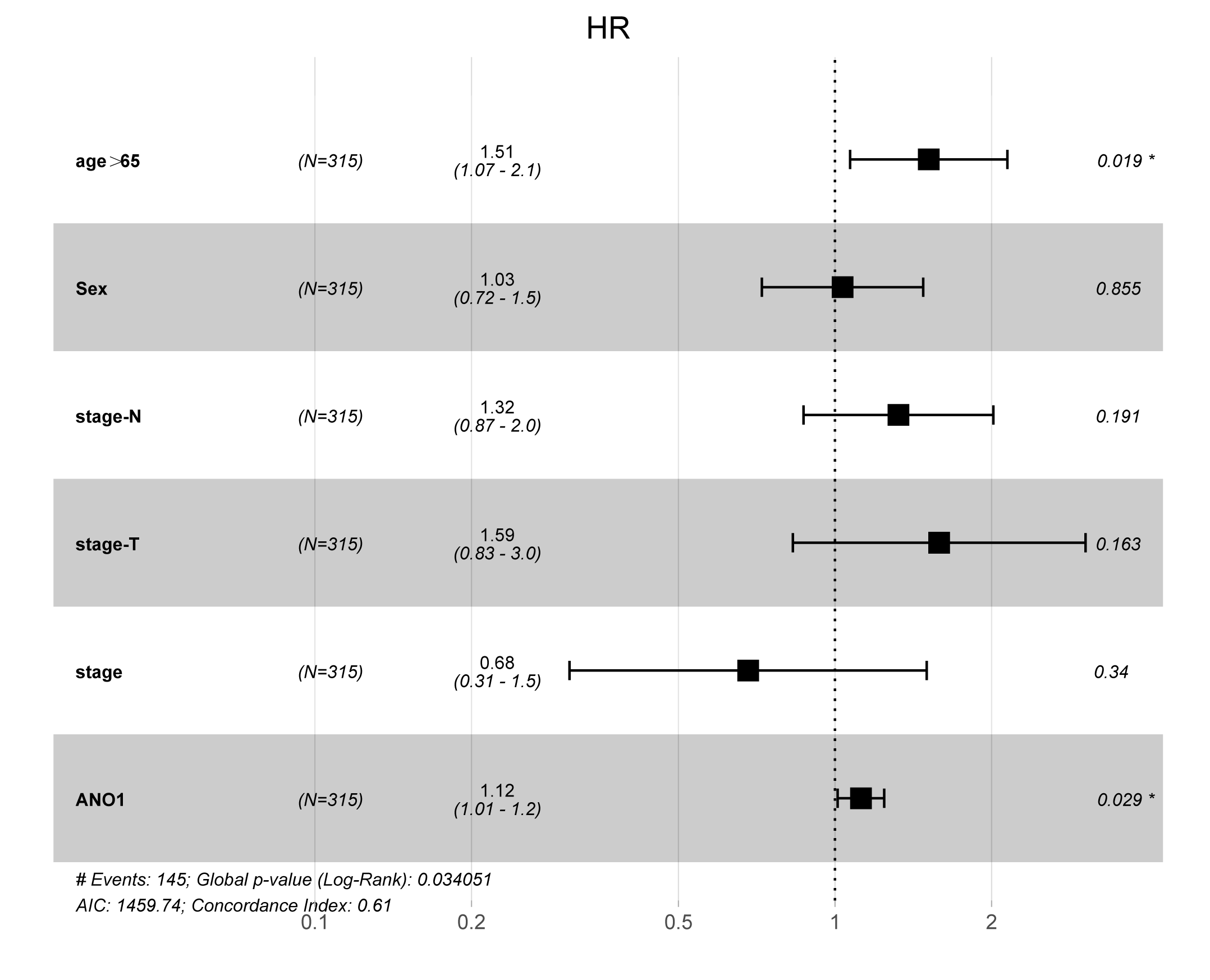

Supplement: S2 File — (ZIP) [file pone.0329622.s002.zip › 多因素Cox分析-46-tiff/ANO1.tif]

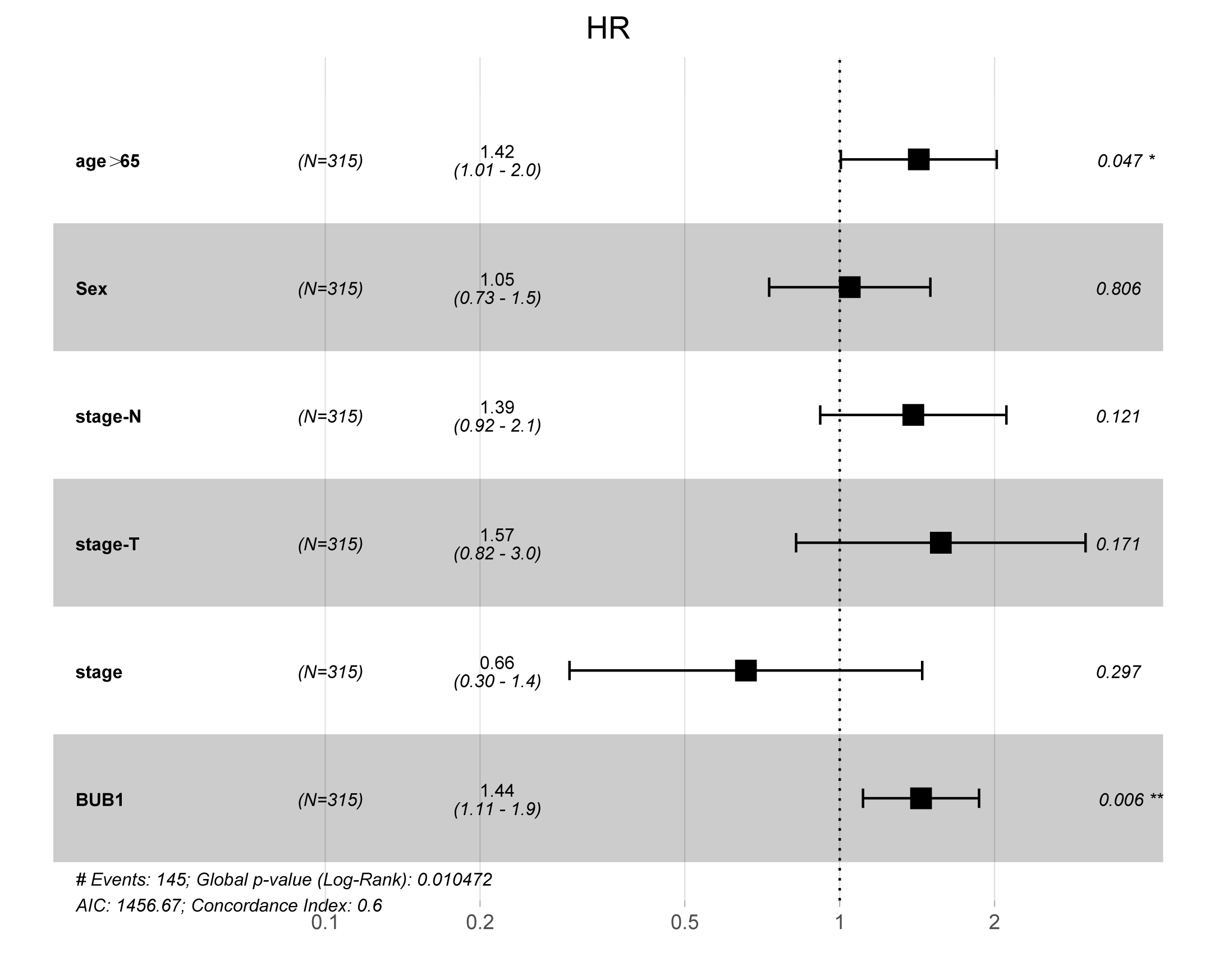

Supplement: S2 File — (ZIP) [file pone.0329622.s002.zip › 多因素Cox分析-46-tiff/BUB1.tif]

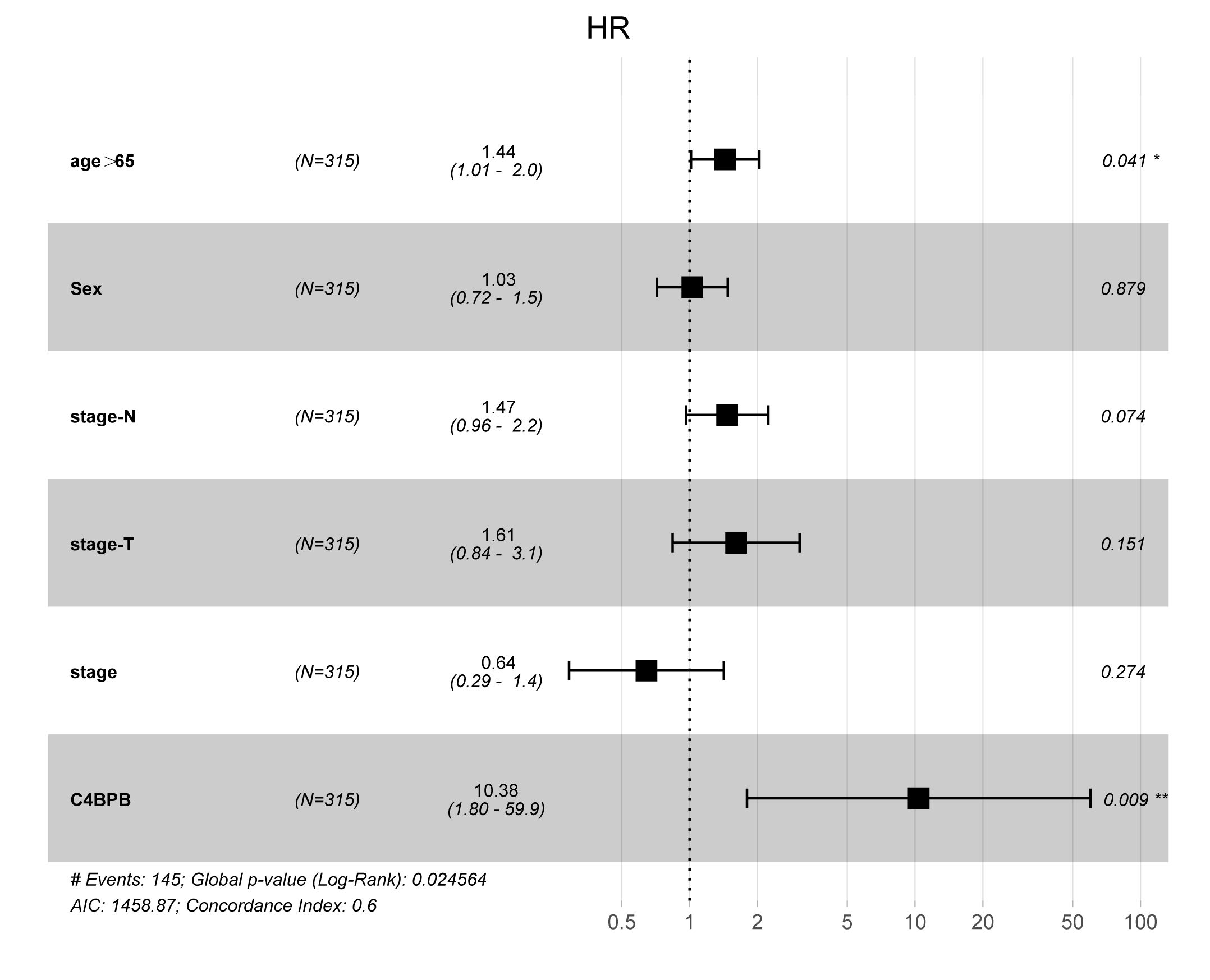

Supplement: S2 File — (ZIP) [file pone.0329622.s002.zip › 多因素Cox分析-46-tiff/C4BPB.tif]

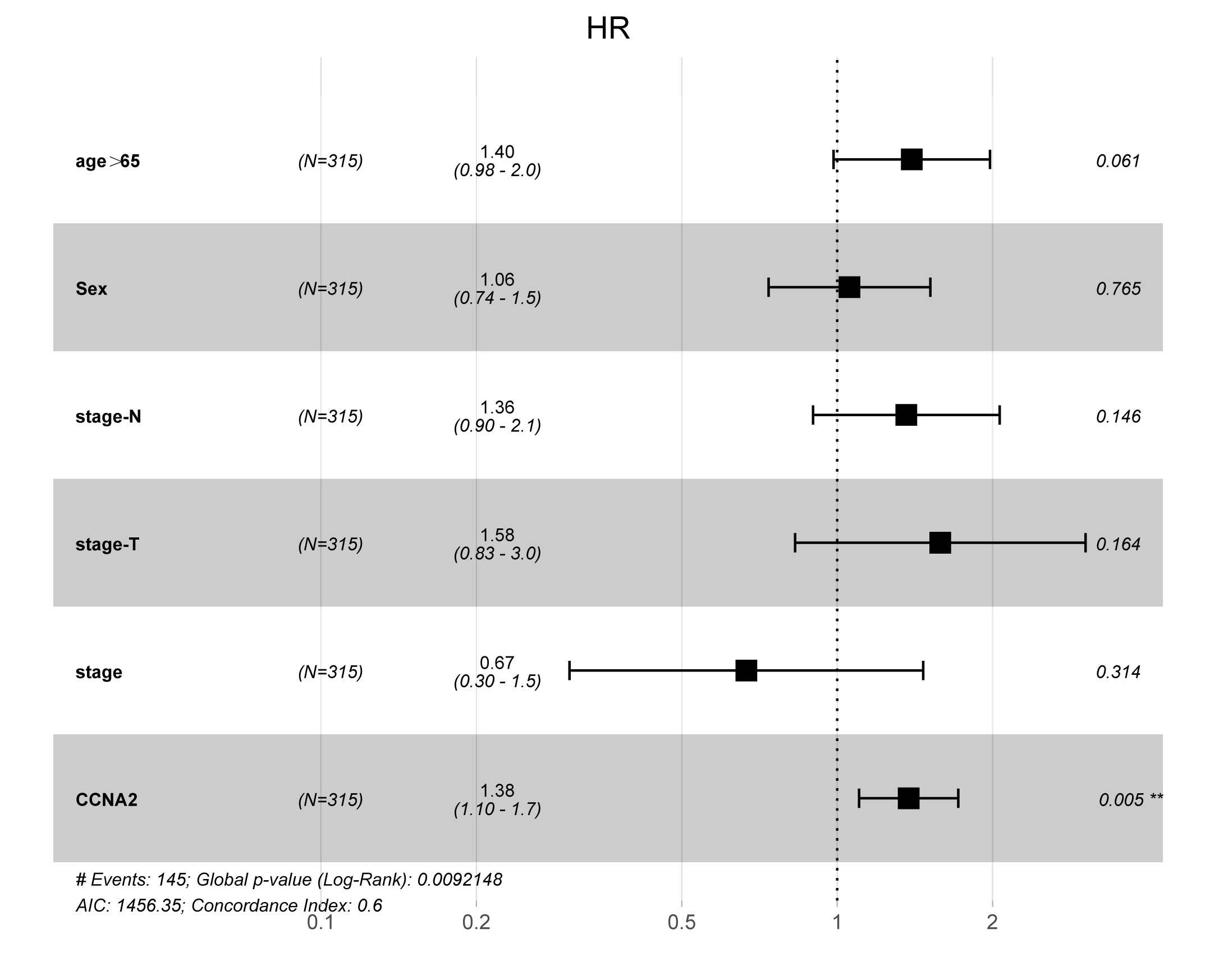

Supplement: S2 File — (ZIP) [file pone.0329622.s002.zip › 多因素Cox分析-46-tiff/CCNA2.tif]

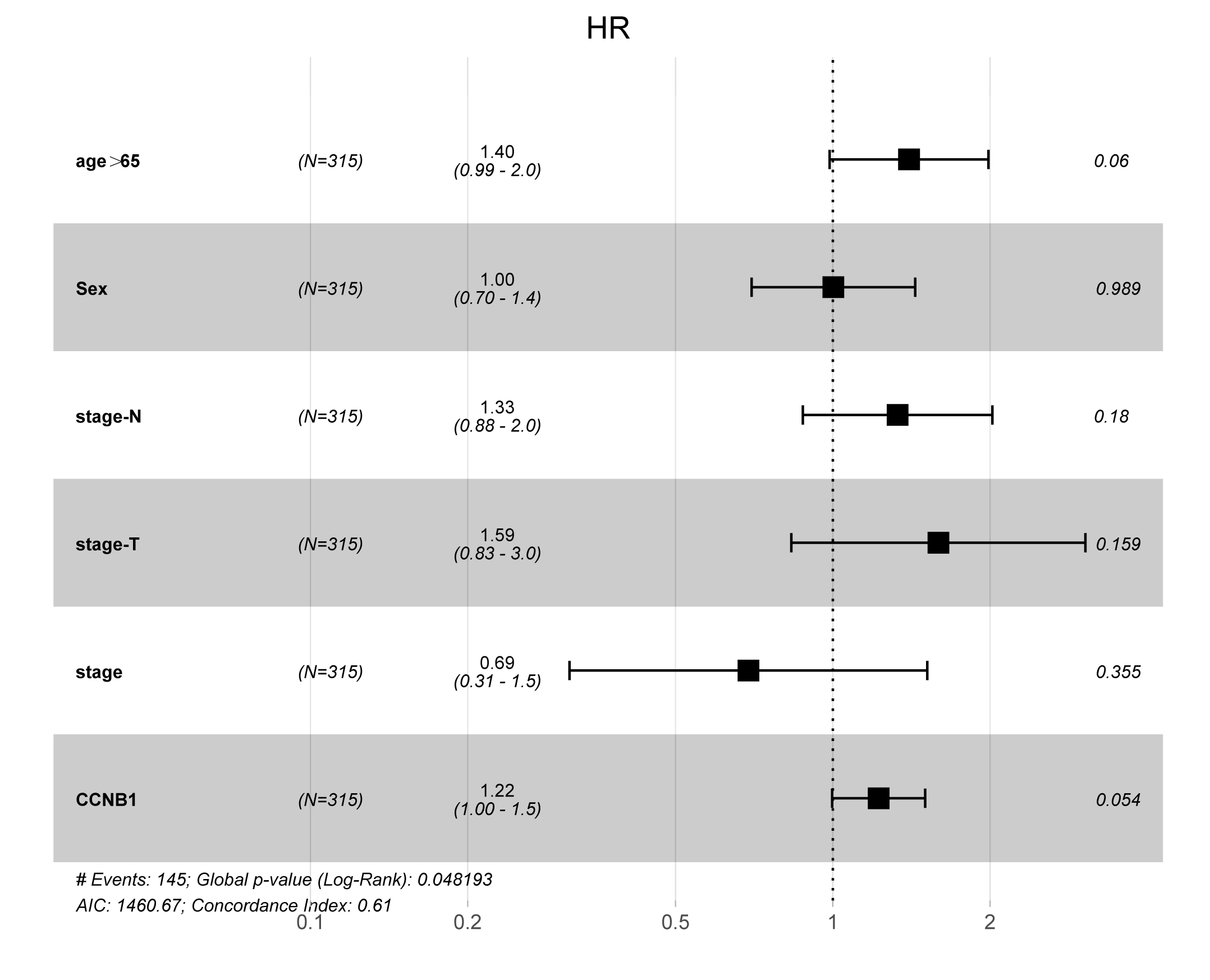

Supplement: S2 File — (ZIP) [file pone.0329622.s002.zip › 多因素Cox分析-46-tiff/CCNB1.tif]

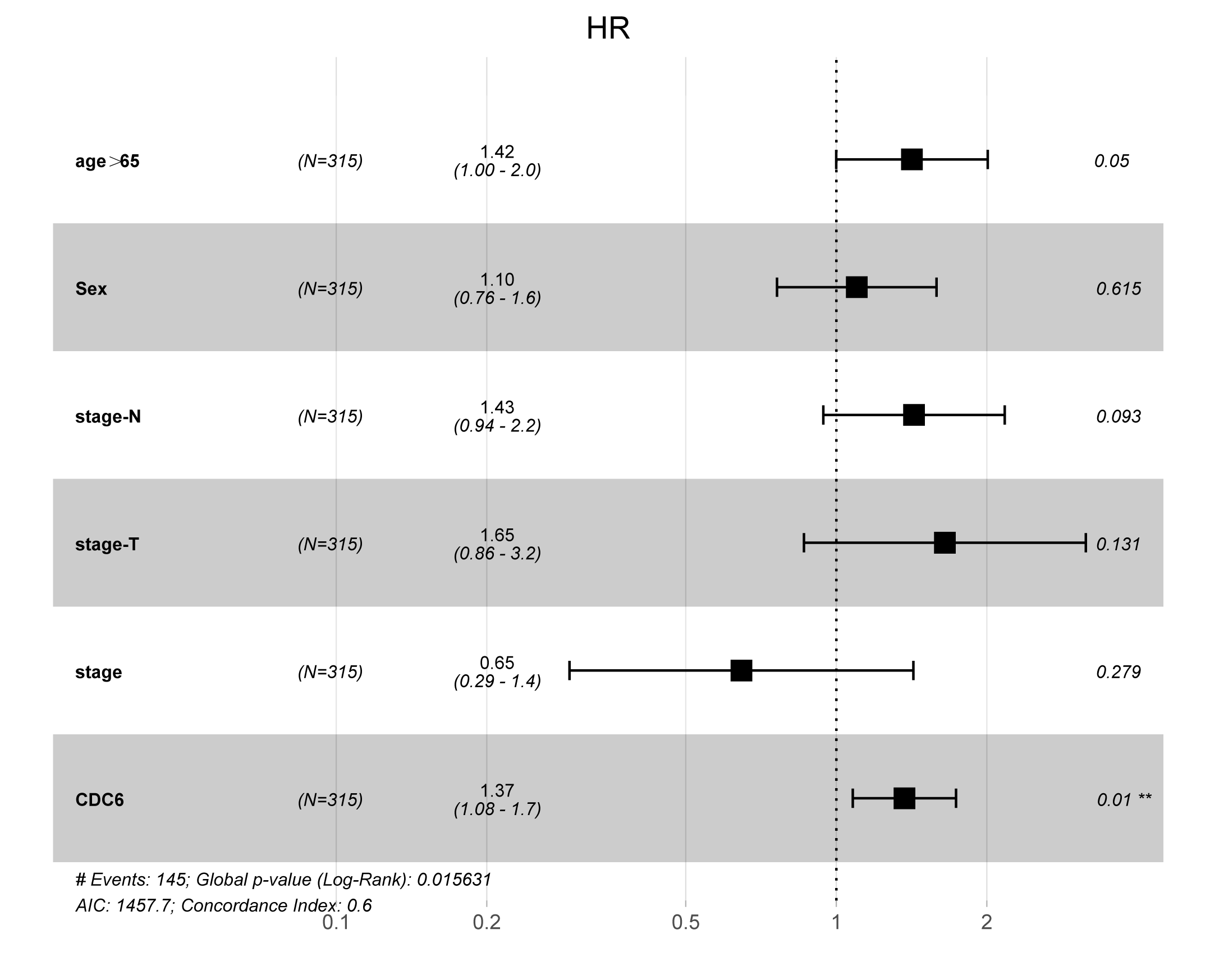

Supplement: S2 File — (ZIP) [file pone.0329622.s002.zip › 多因素Cox分析-46-tiff/CDC6.tif]

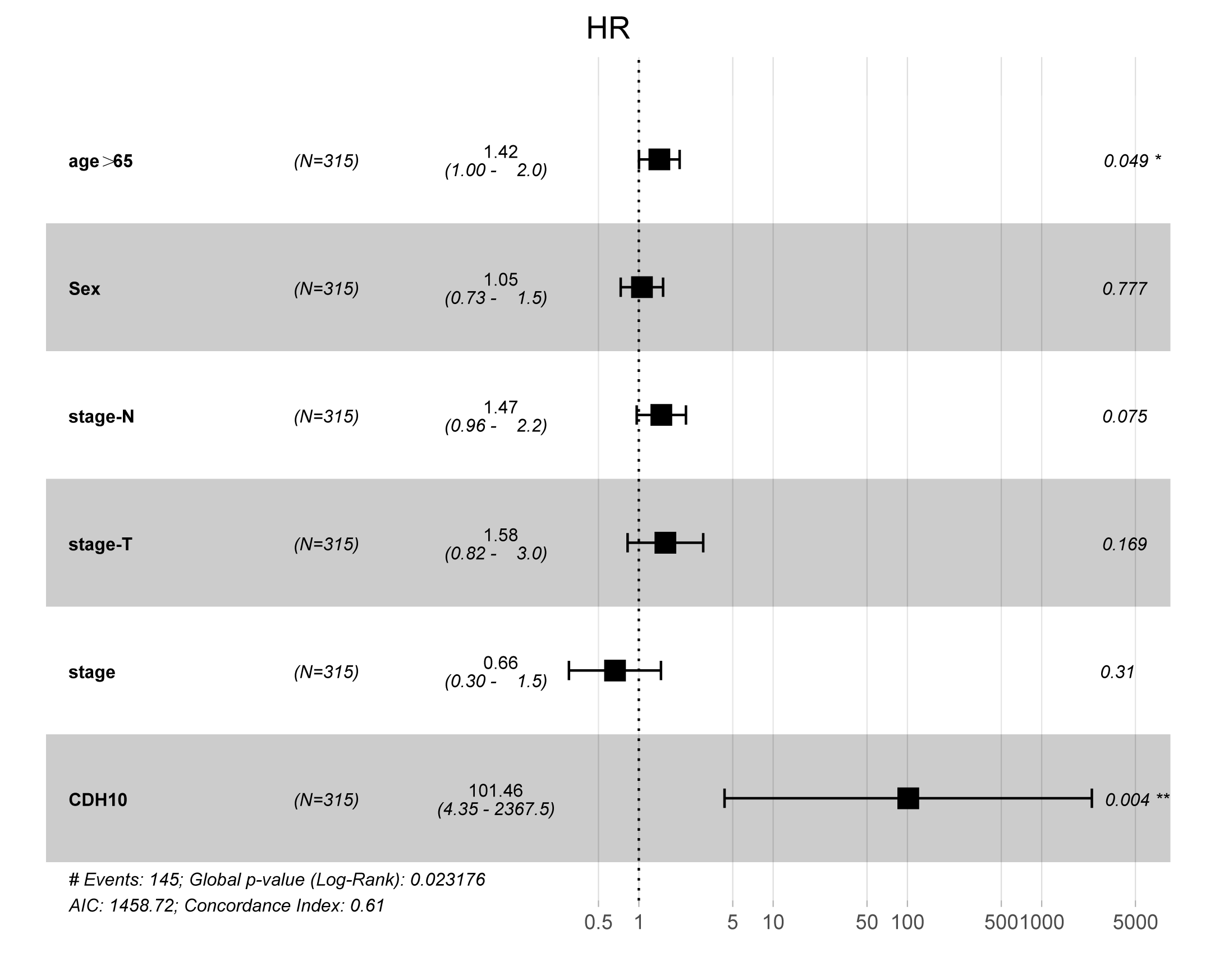

Supplement: S2 File — (ZIP) [file pone.0329622.s002.zip › 多因素Cox分析-46-tiff/CDH10.tif]

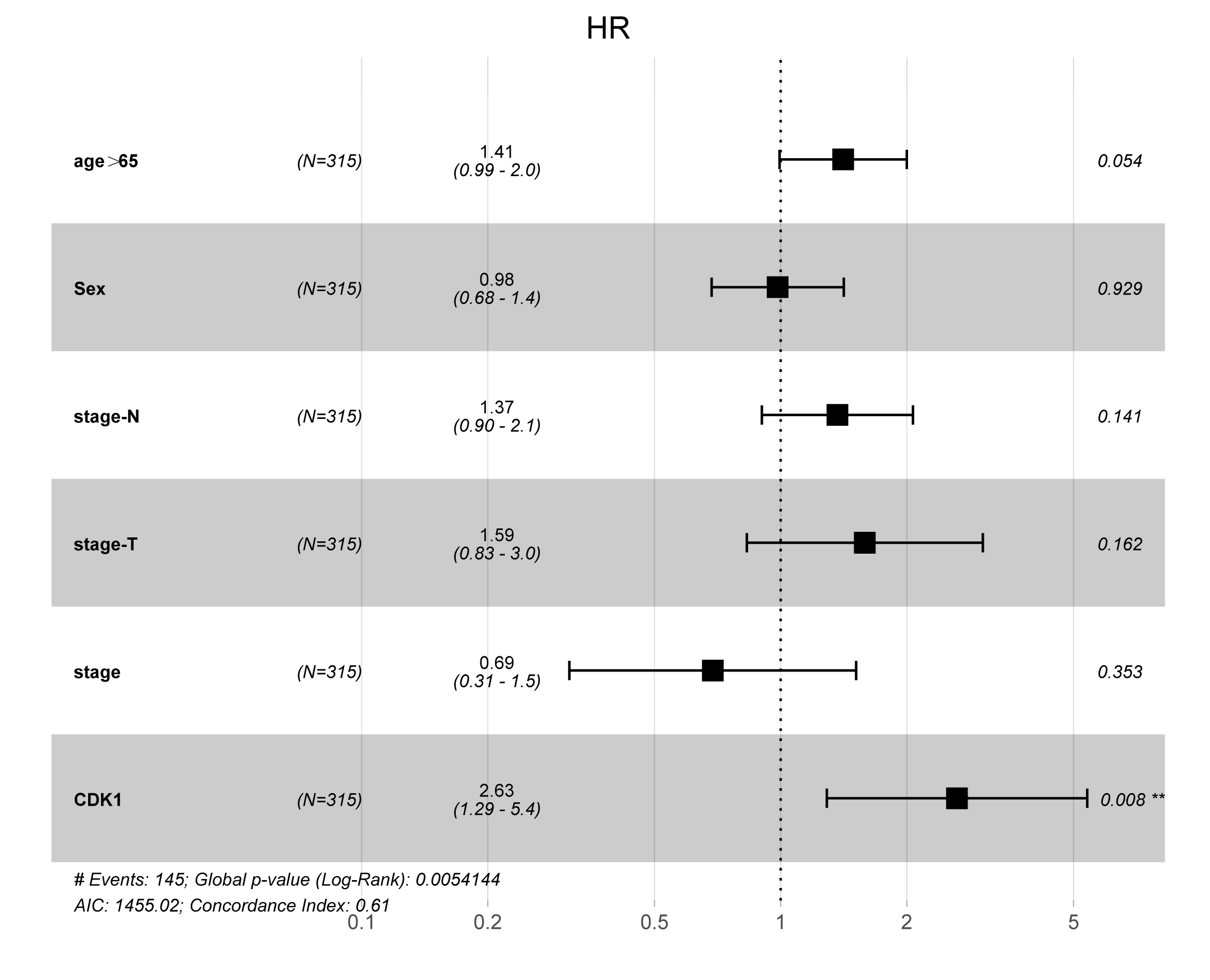

Supplement: S2 File — (ZIP) [file pone.0329622.s002.zip › 多因素Cox分析-46-tiff/CDK1.tif]

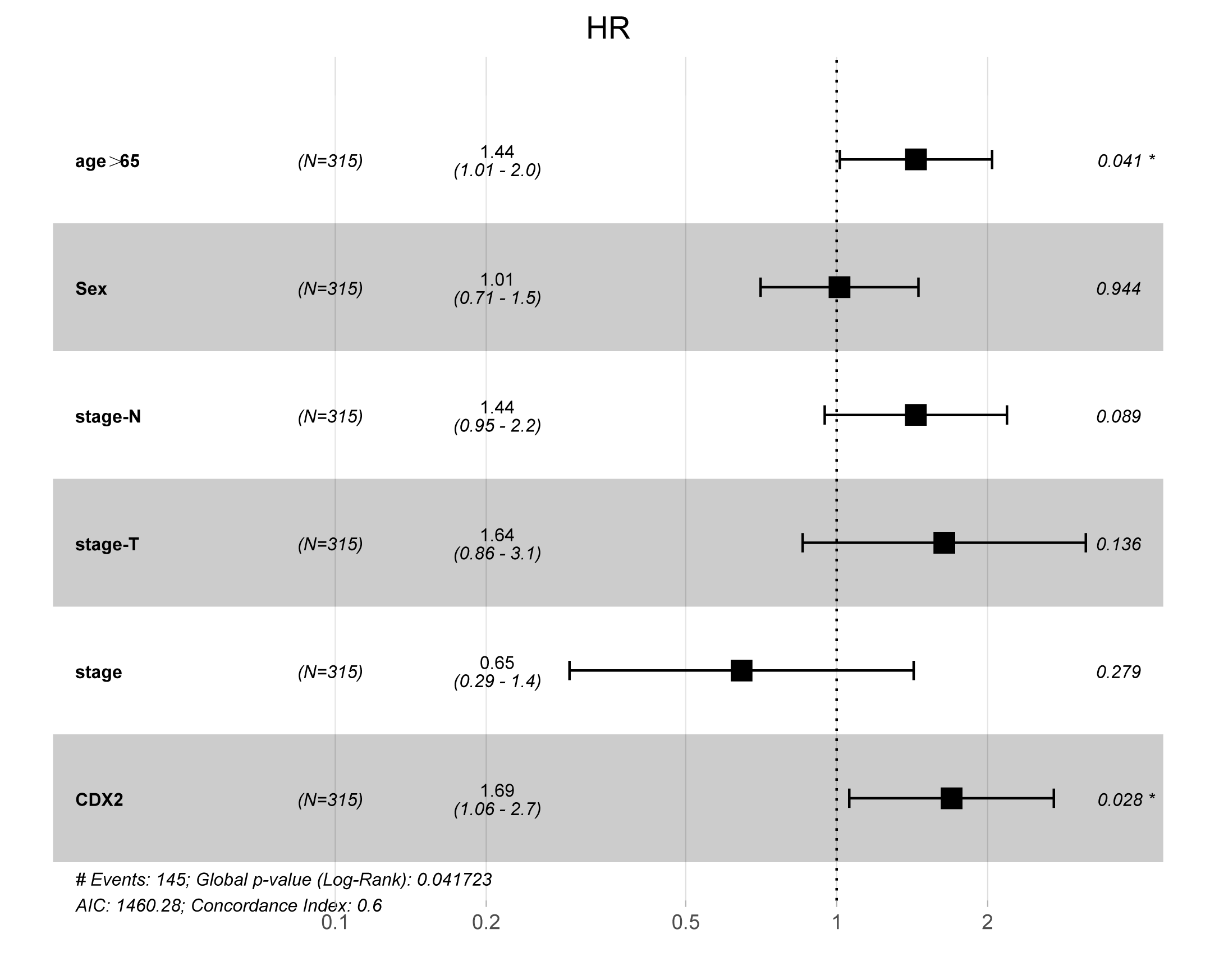

Supplement: S2 File — (ZIP) [file pone.0329622.s002.zip › 多因素Cox分析-46-tiff/CDX2.tif]

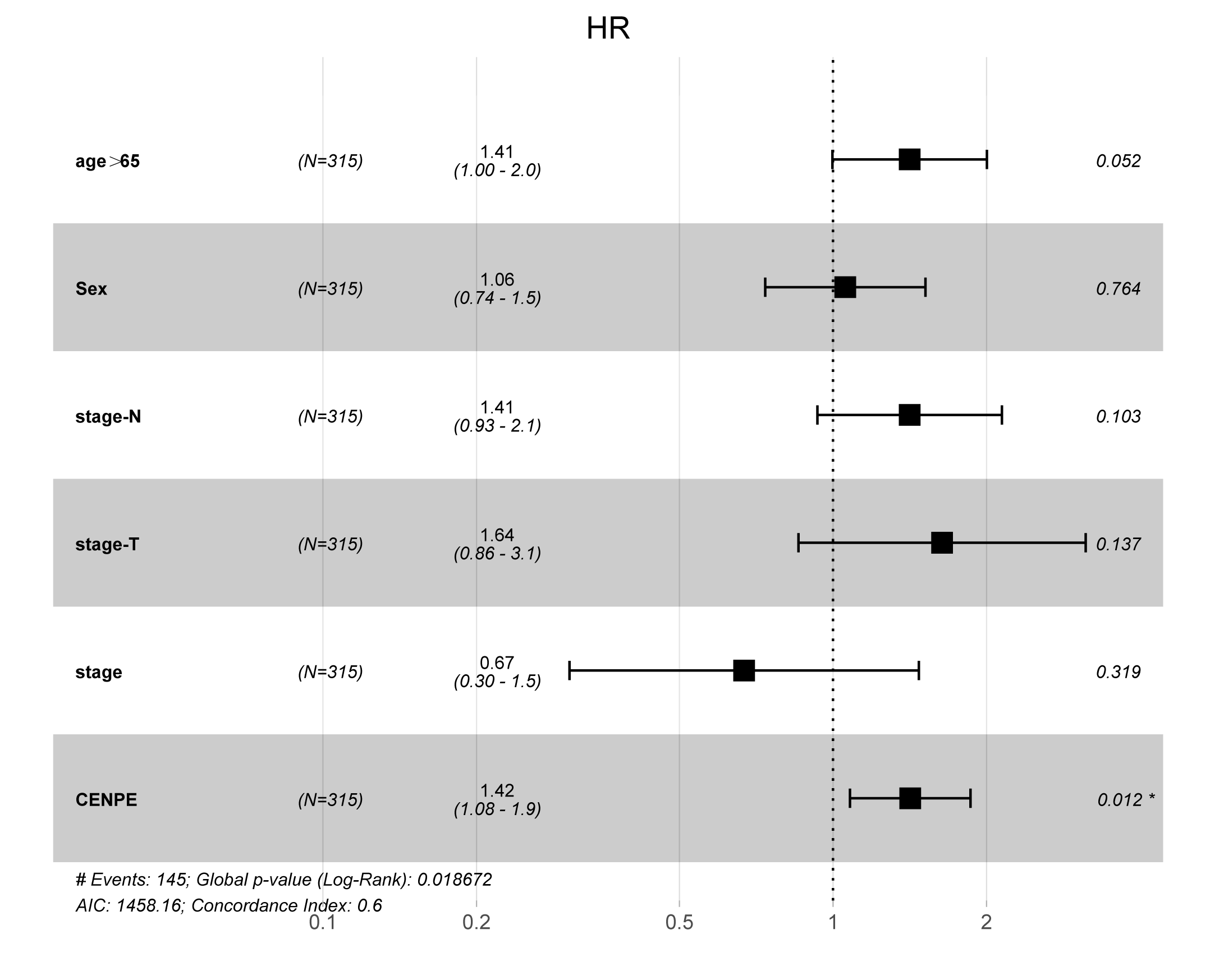

Supplement: S2 File — (ZIP) [file pone.0329622.s002.zip › 多因素Cox分析-46-tiff/CENPE.tif]

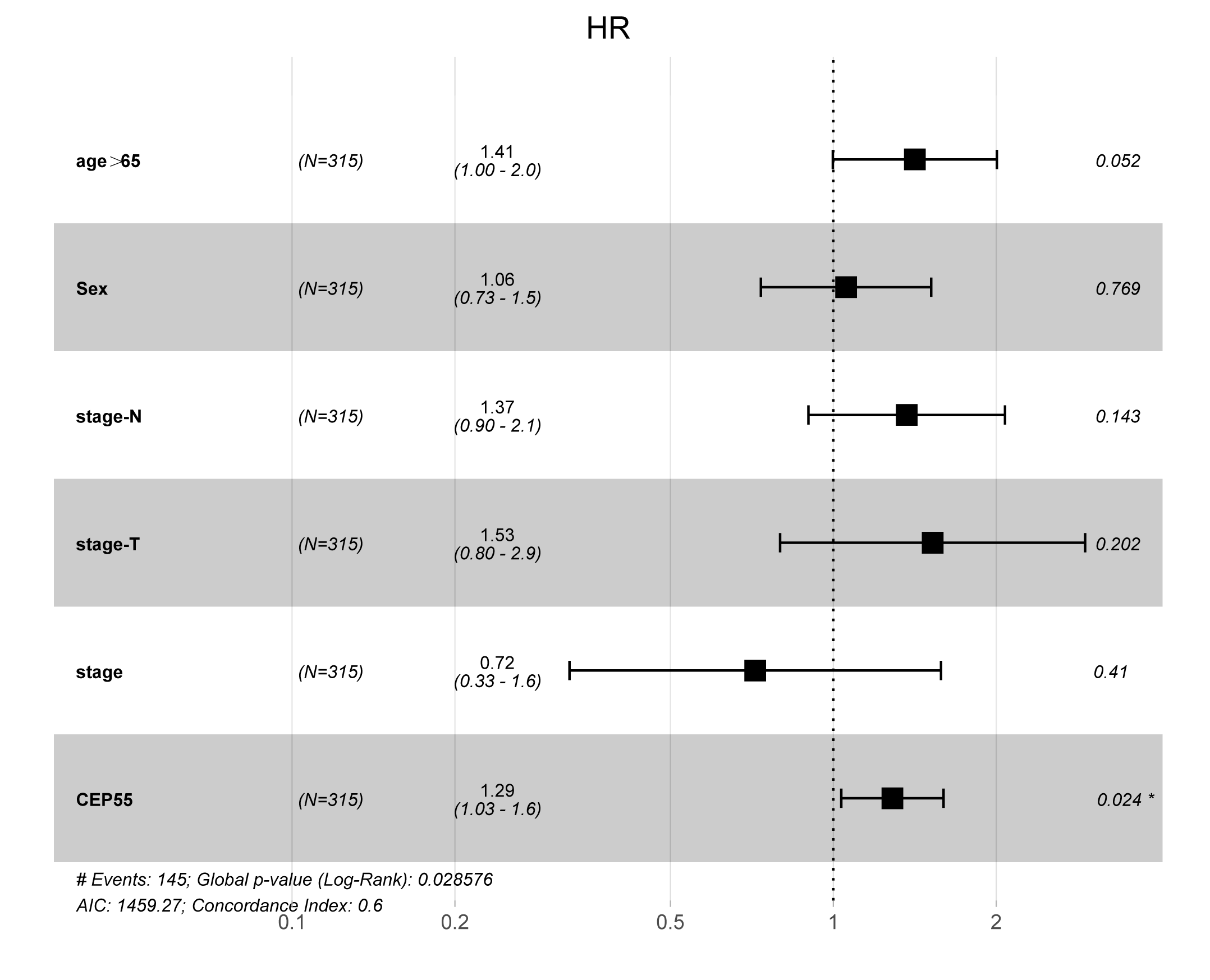

Supplement: S2 File — (ZIP) [file pone.0329622.s002.zip › 多因素Cox分析-46-tiff/CEP55.tif]

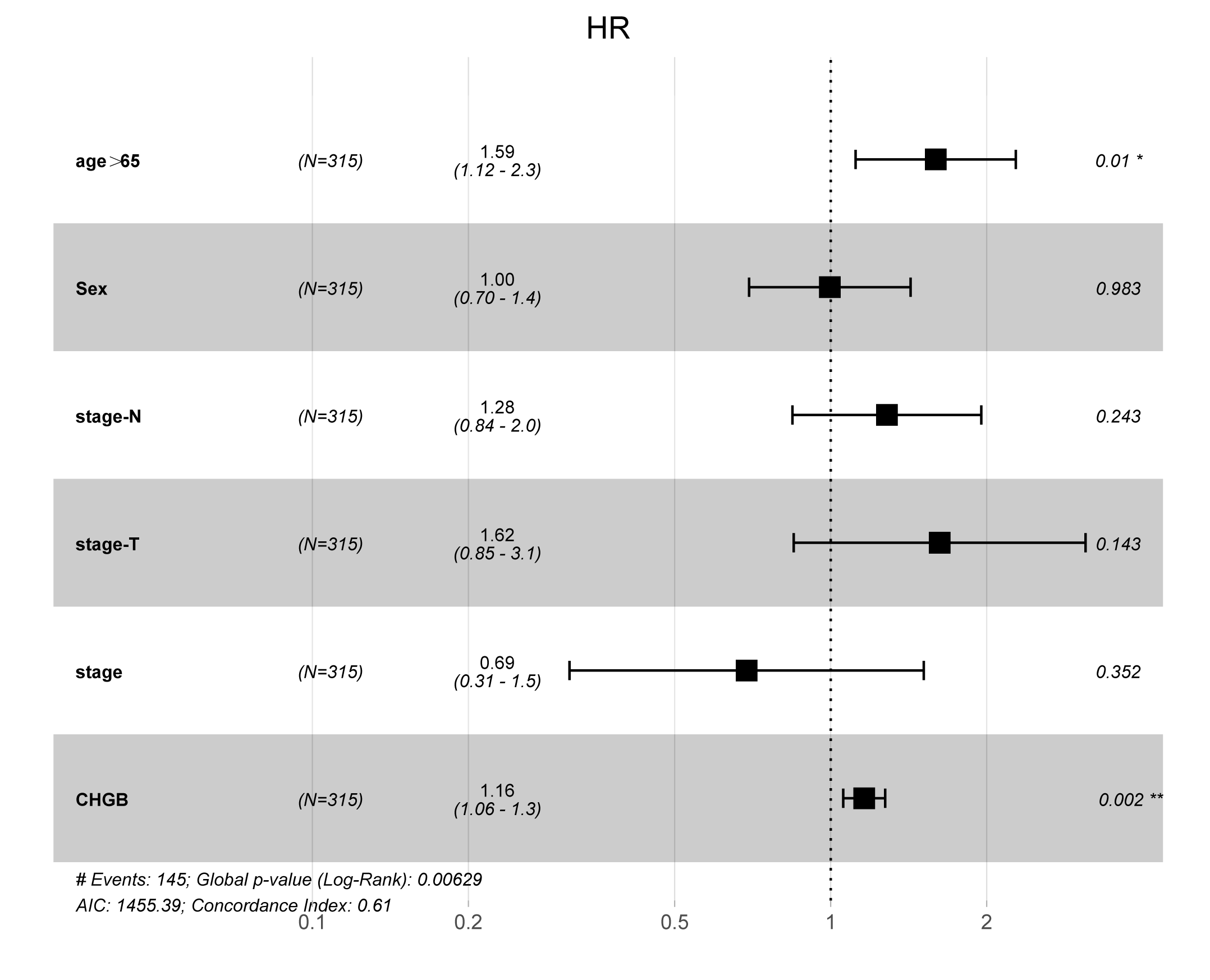

Supplement: S2 File — (ZIP) [file pone.0329622.s002.zip › 多因素Cox分析-46-tiff/CHGB.tif]

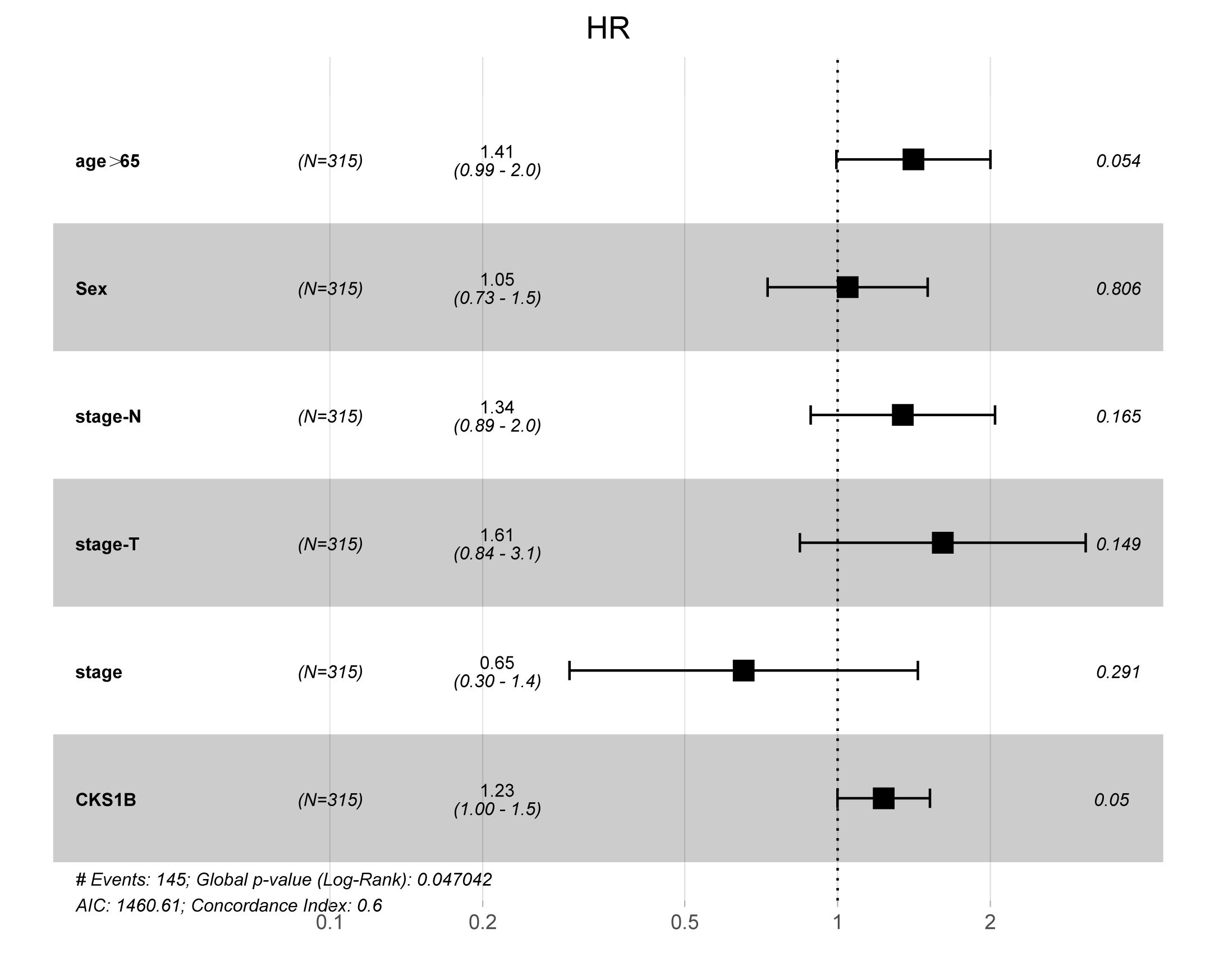

Supplement: S2 File — (ZIP) [file pone.0329622.s002.zip › 多因素Cox分析-46-tiff/CKS1B.tif]

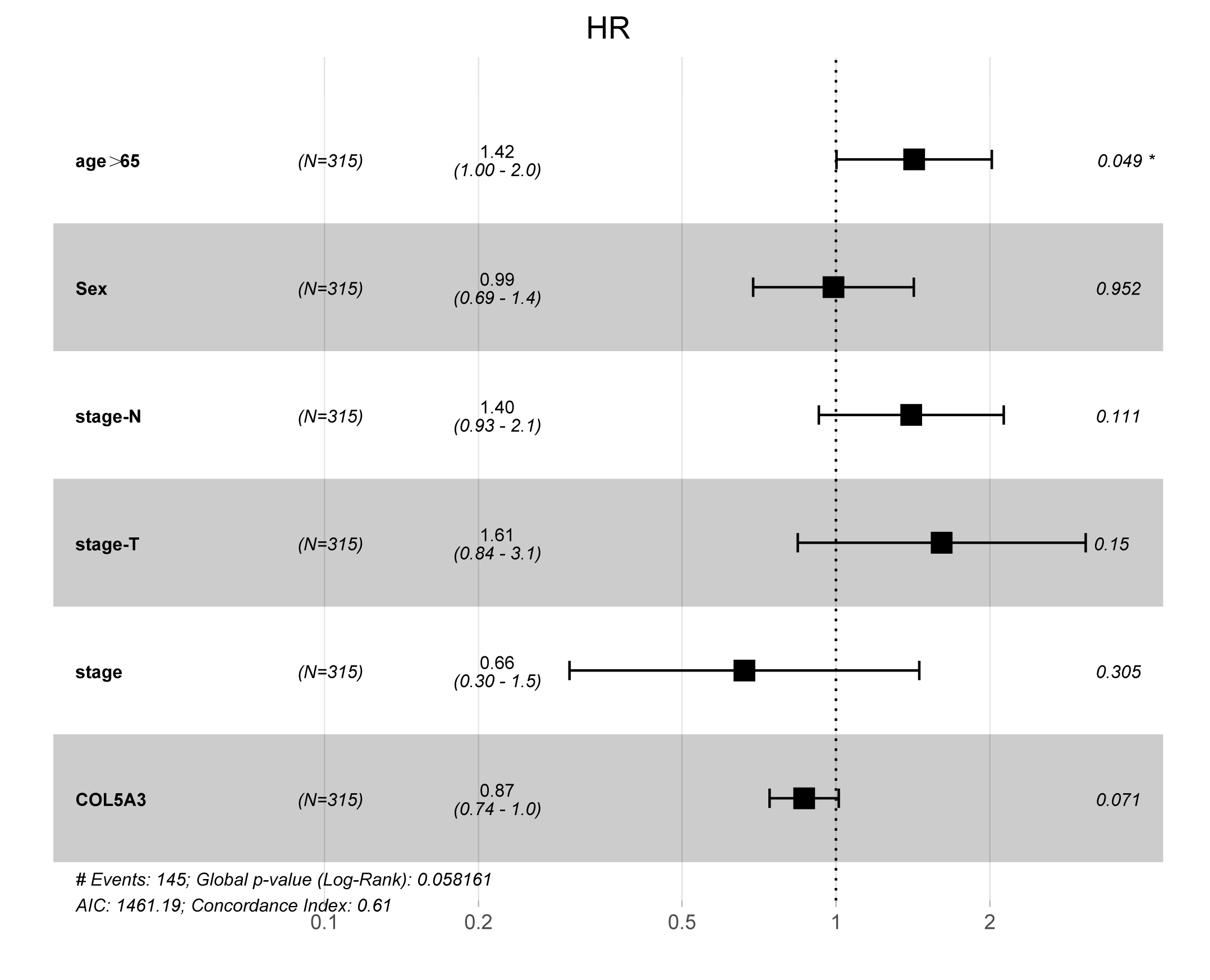

Supplement: S2 File — (ZIP) [file pone.0329622.s002.zip › 多因素Cox分析-46-tiff/COL5A3.tif]

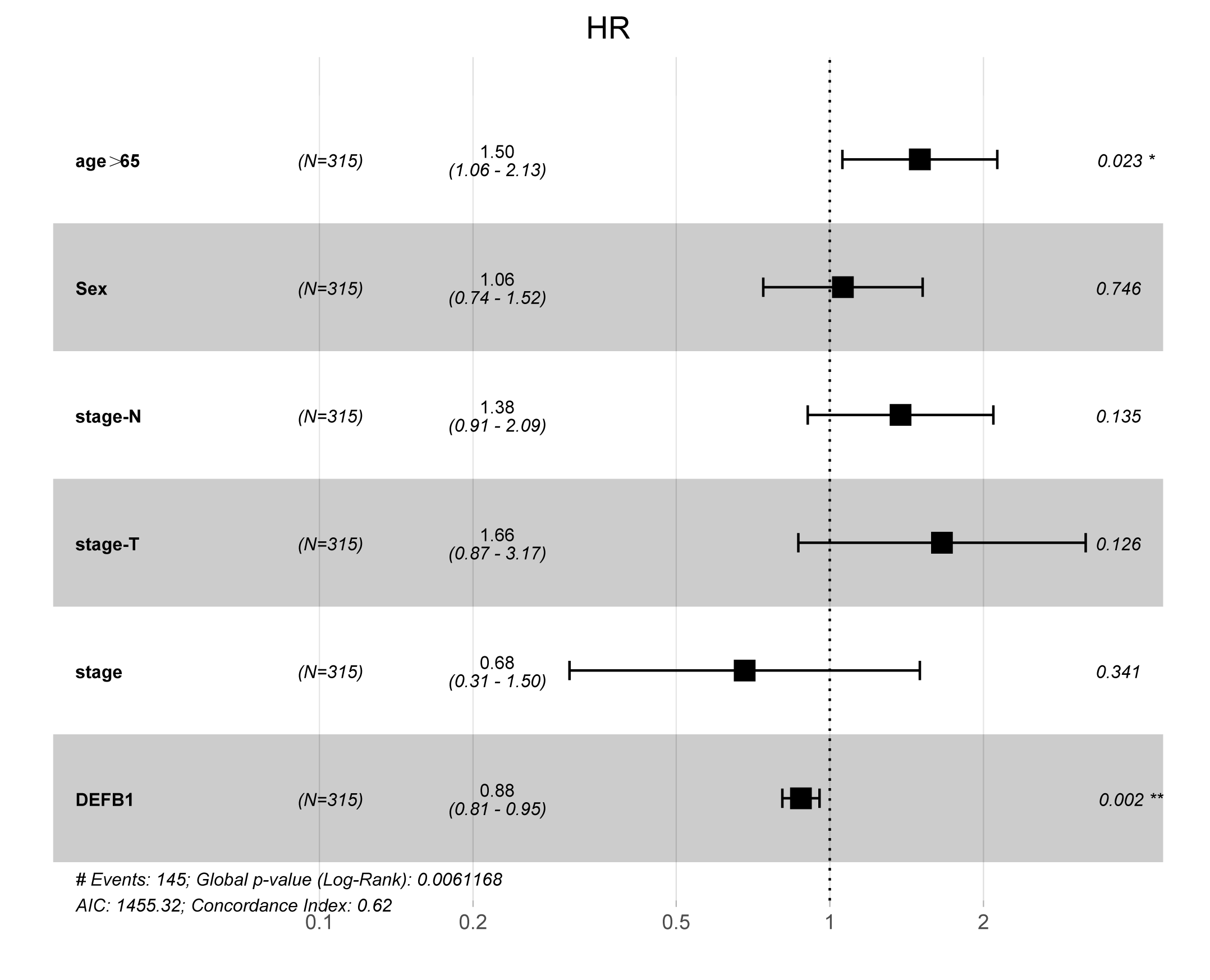

Supplement: S2 File — (ZIP) [file pone.0329622.s002.zip › 多因素Cox分析-46-tiff/DEFB1.tif]

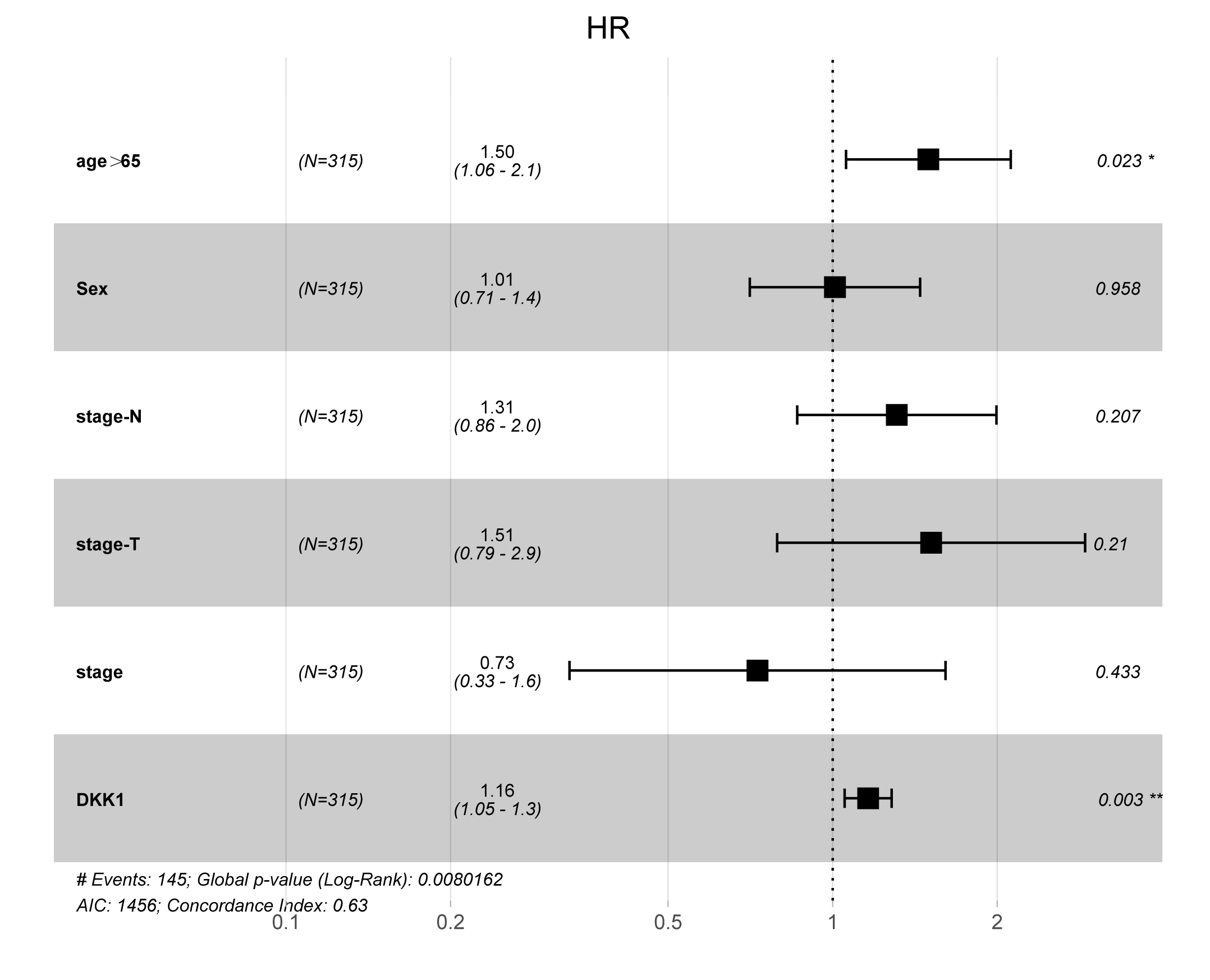

Supplement: S2 File — (ZIP) [file pone.0329622.s002.zip › 多因素Cox分析-46-tiff/DKK1.tif]

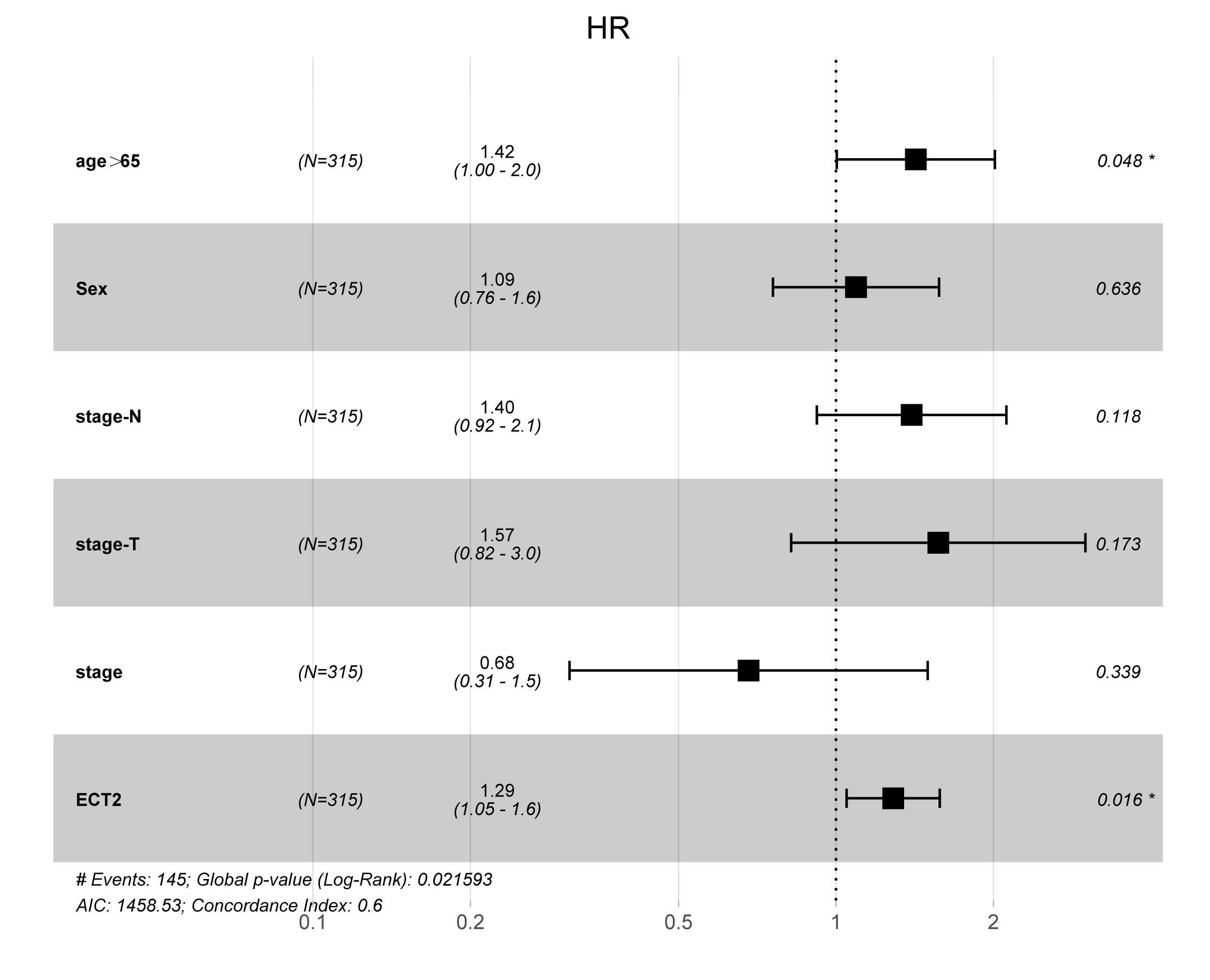

Supplement: S2 File — (ZIP) [file pone.0329622.s002.zip › 多因素Cox分析-46-tiff/ECT2.tif]

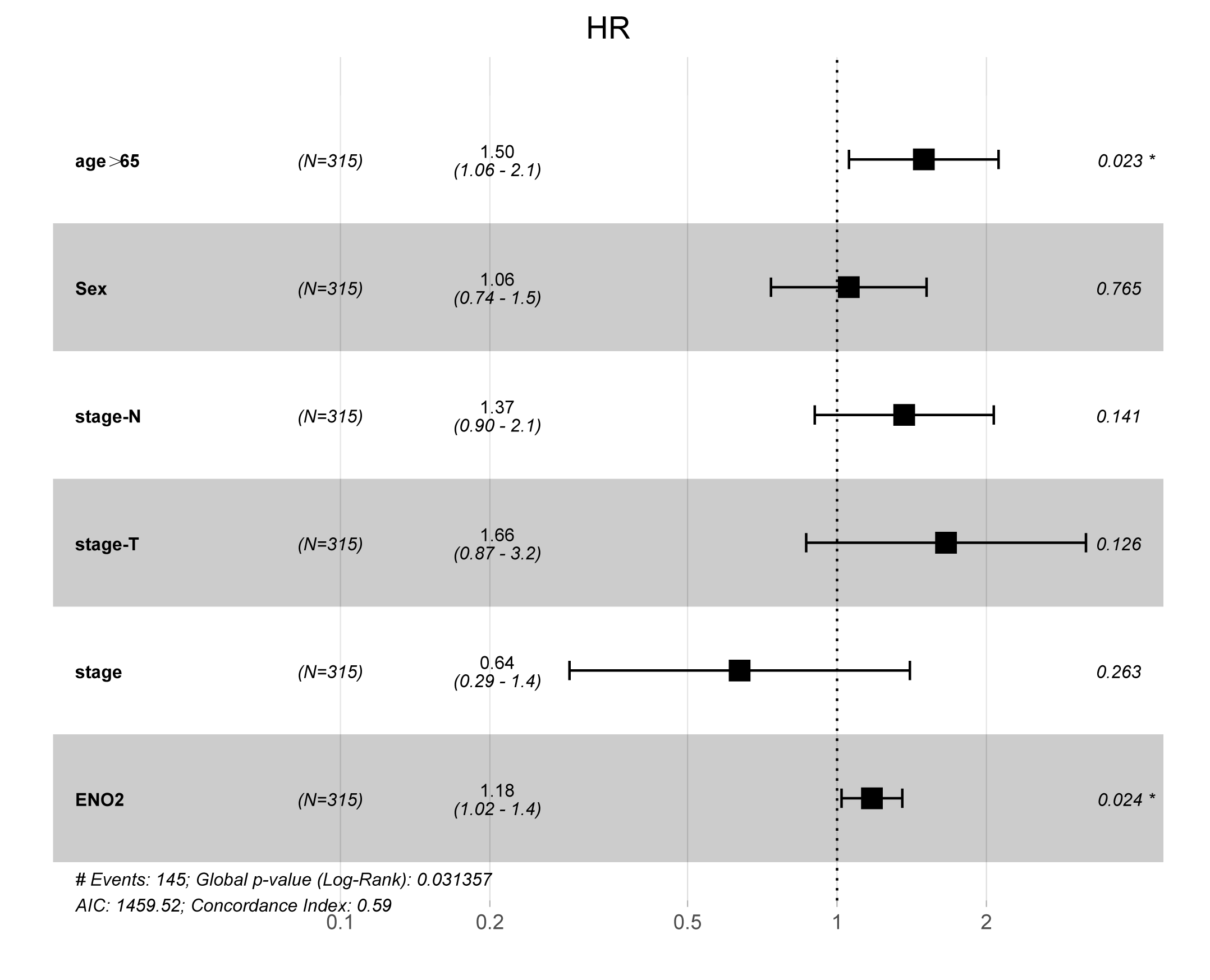

Supplement: S2 File — (ZIP) [file pone.0329622.s002.zip › 多因素Cox分析-46-tiff/ENO2.tif]

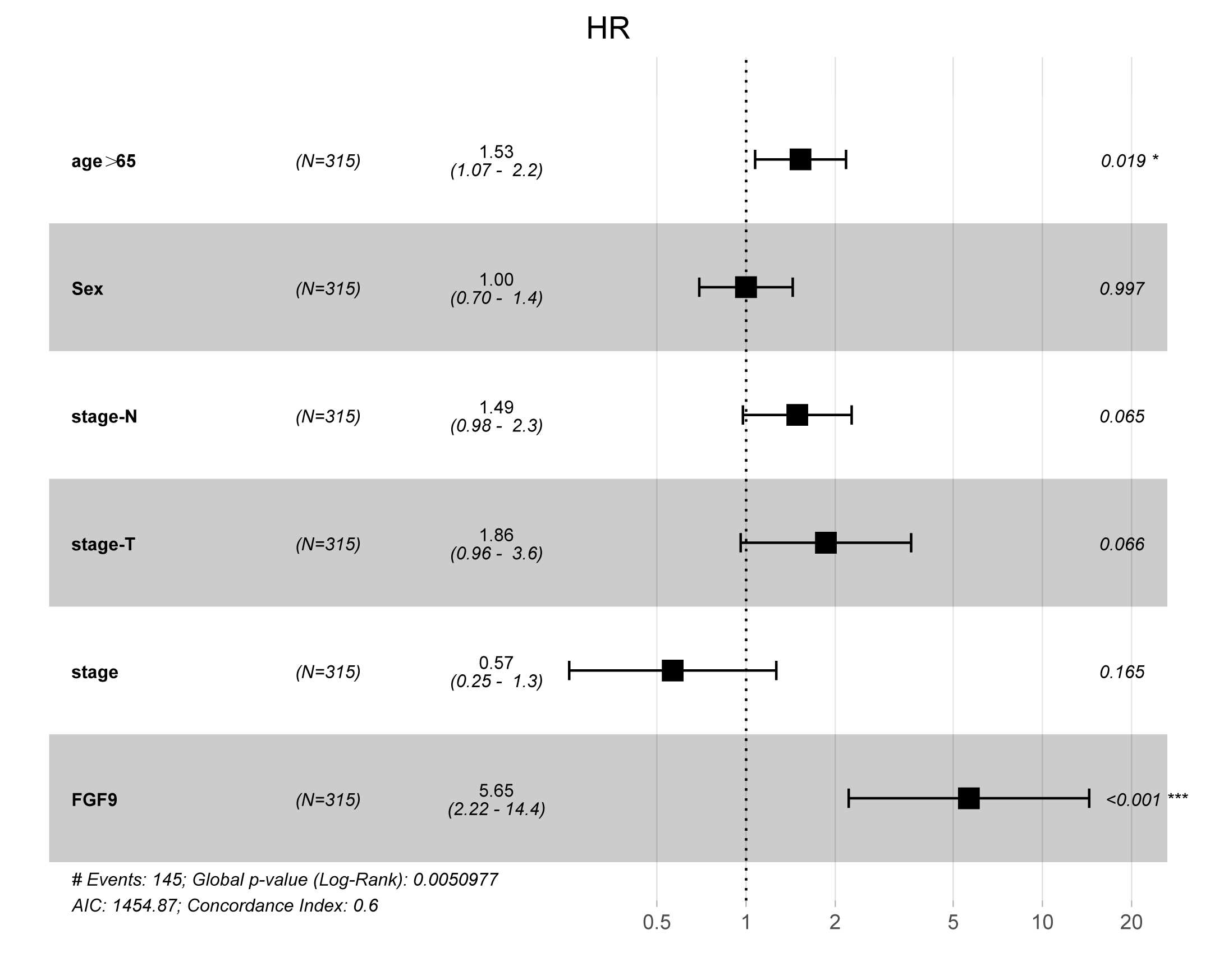

Supplement: S2 File — (ZIP) [file pone.0329622.s002.zip › 多因素Cox分析-46-tiff/FGF9.tif]

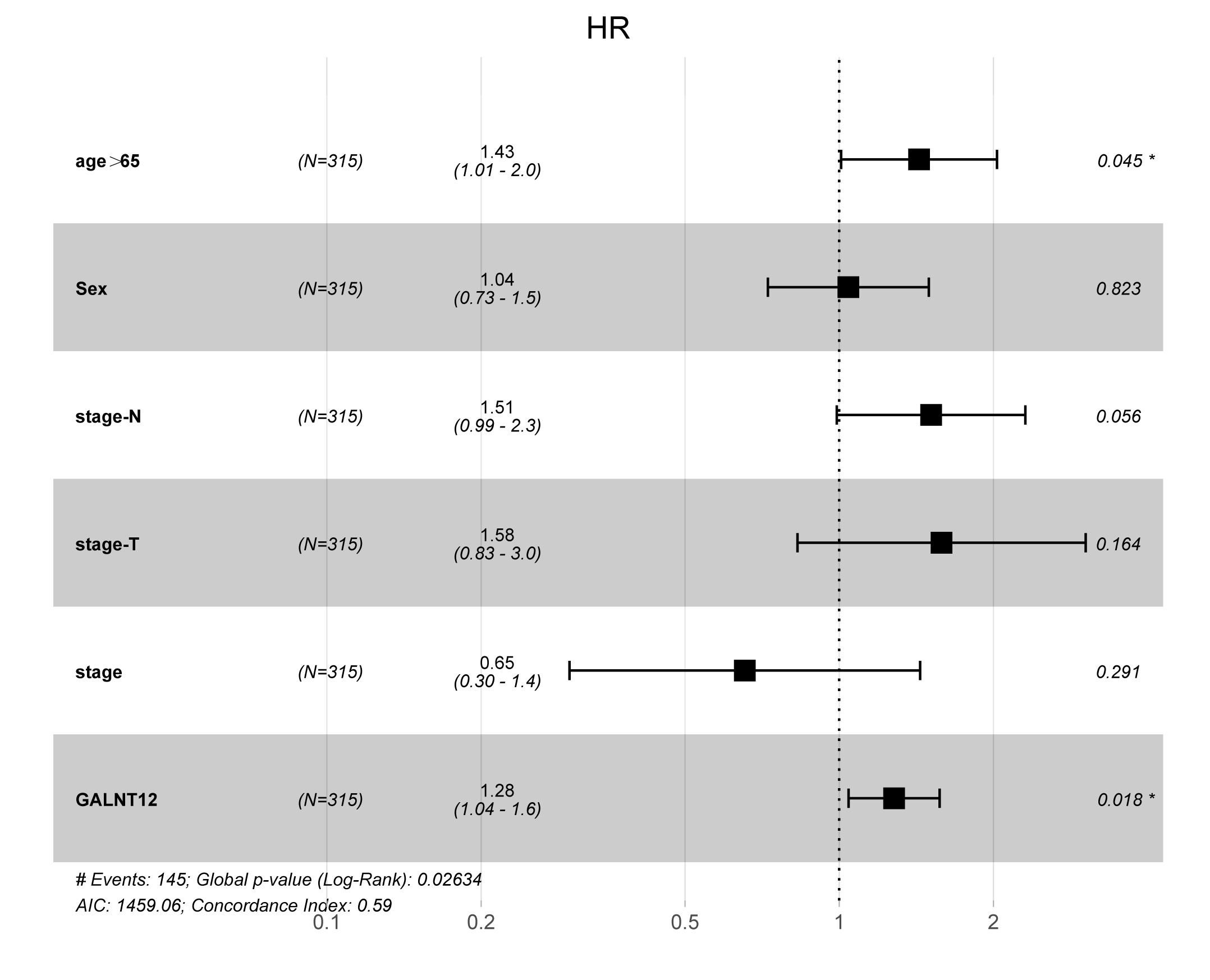

Supplement: S2 File — (ZIP) [file pone.0329622.s002.zip › 多因素Cox分析-46-tiff/GALNT12.tif]

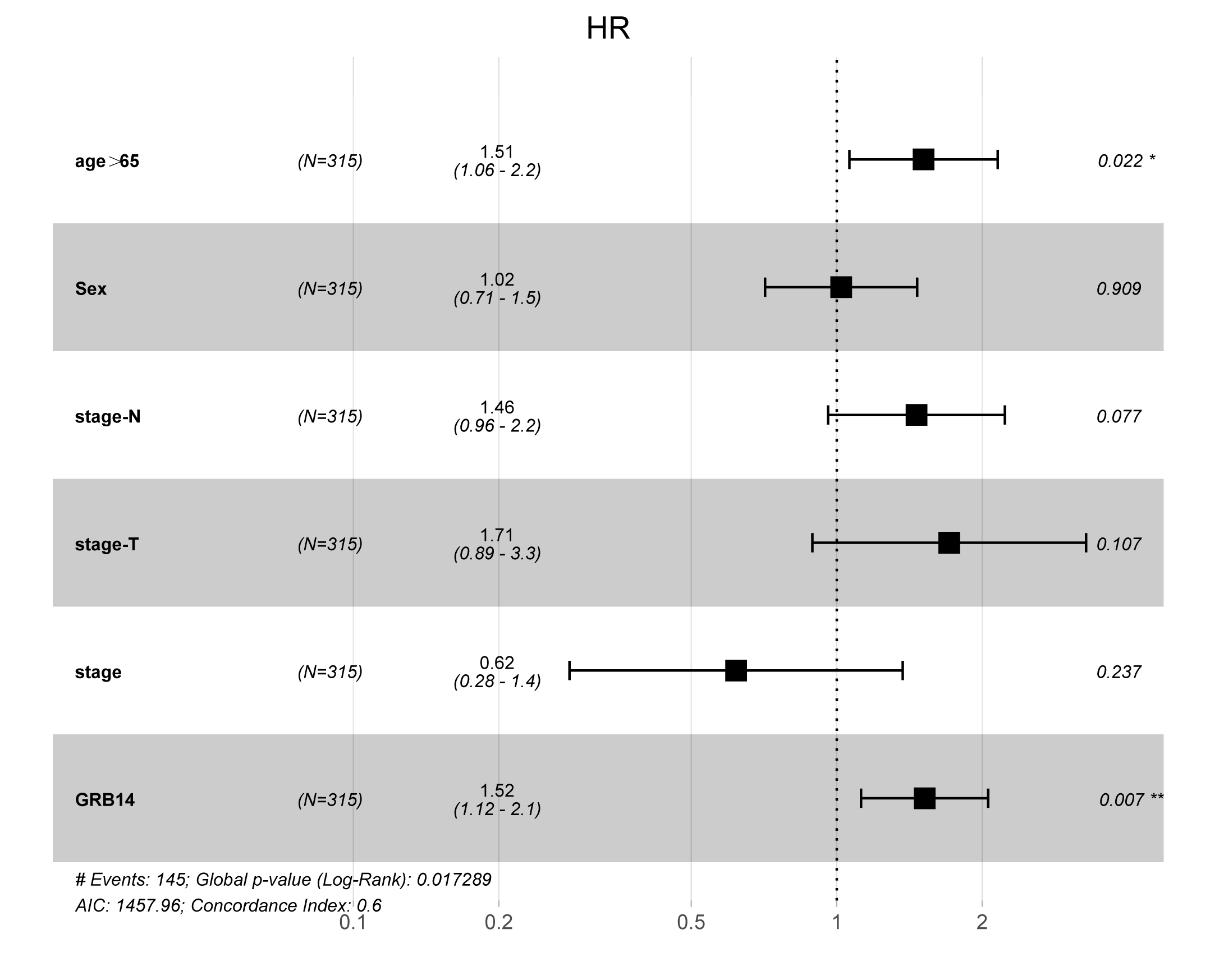

Supplement: S2 File — (ZIP) [file pone.0329622.s002.zip › 多因素Cox分析-46-tiff/GRB14.tif]

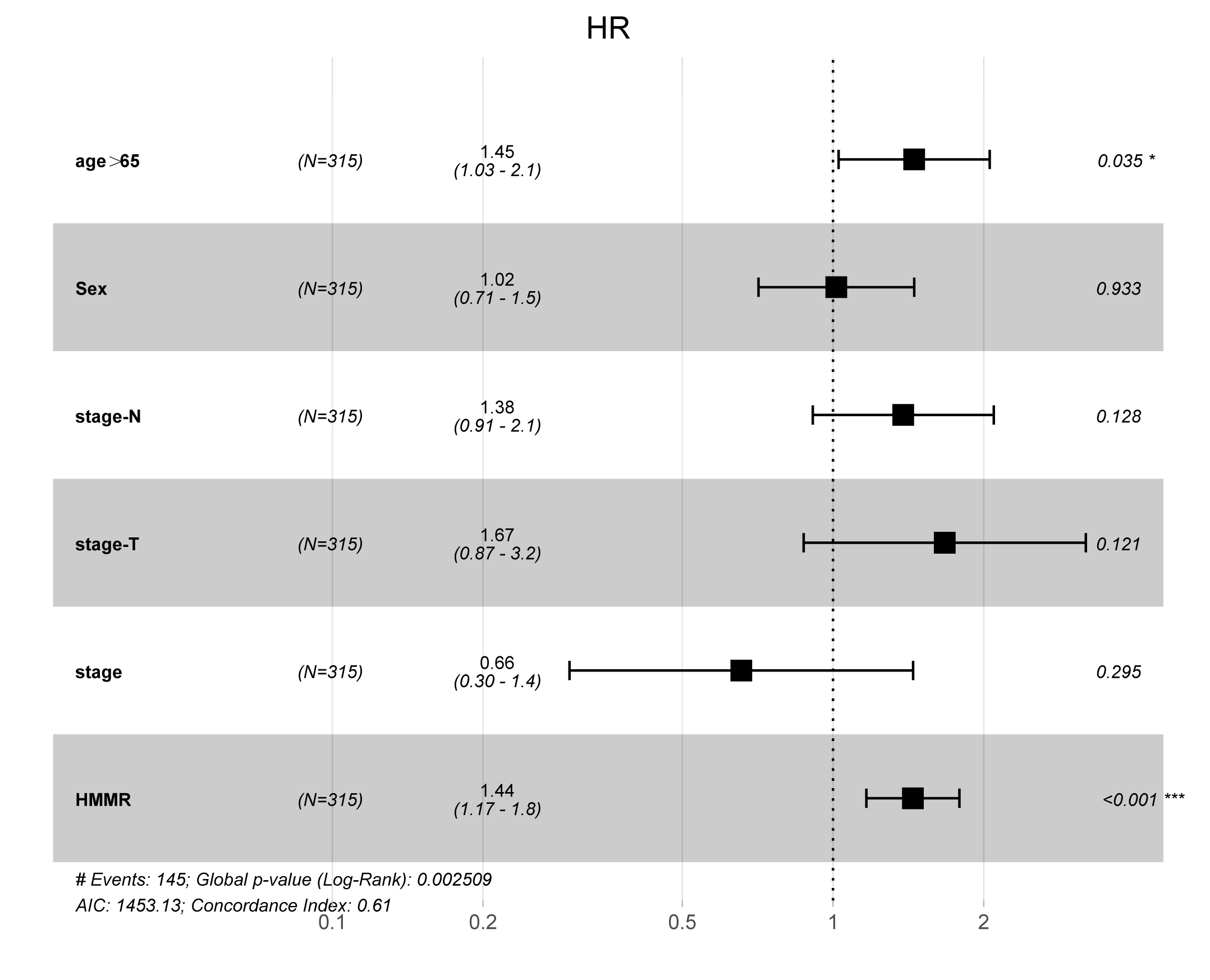

Supplement: S2 File — (ZIP) [file pone.0329622.s002.zip › 多因素Cox分析-46-tiff/HMMR.tif]

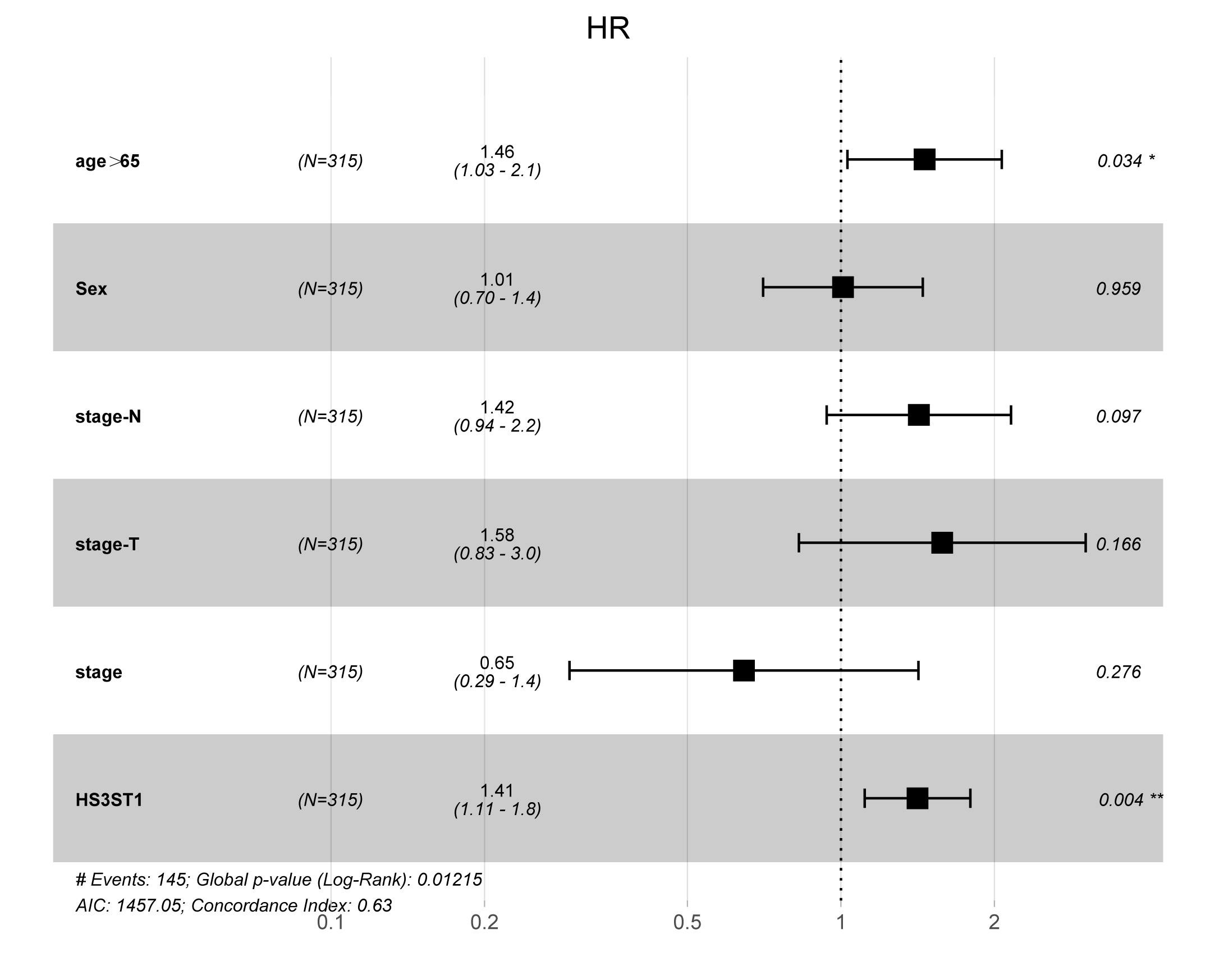

Supplement: S2 File — (ZIP) [file pone.0329622.s002.zip › 多因素Cox分析-46-tiff/HS3ST1.tif]

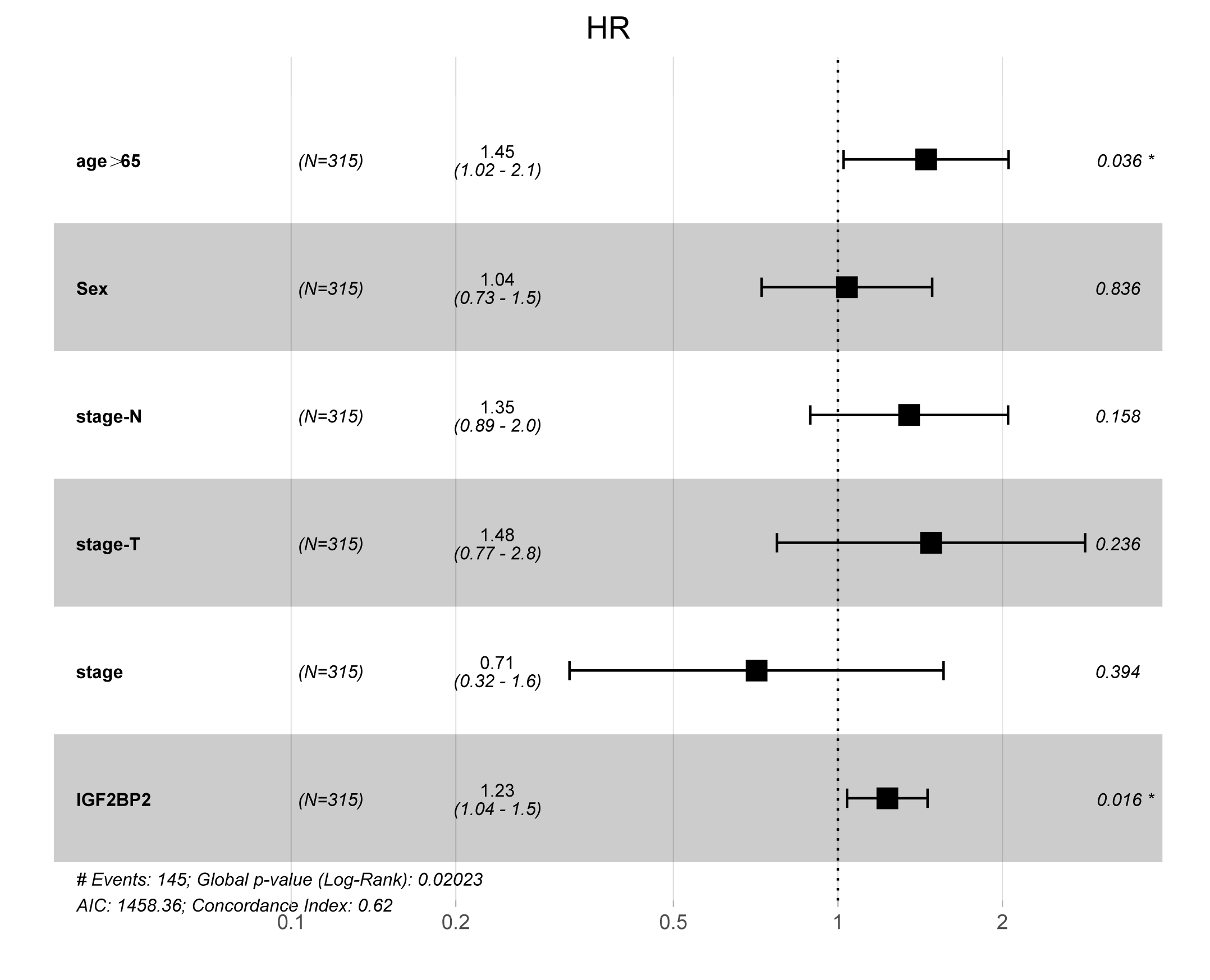

Supplement: S2 File — (ZIP) [file pone.0329622.s002.zip › 多因素Cox分析-46-tiff/IGF2BP2.tif]

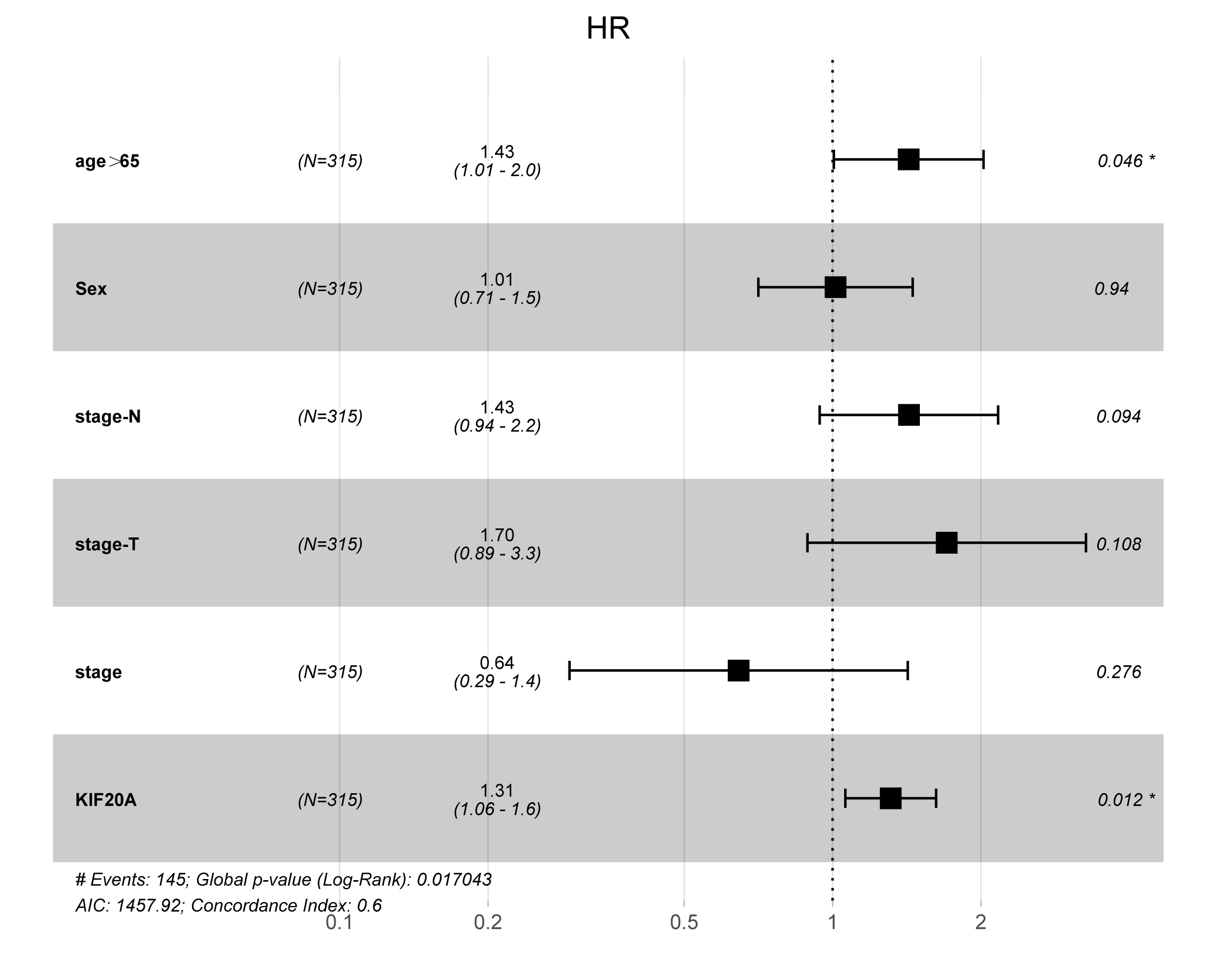

Supplement: S2 File — (ZIP) [file pone.0329622.s002.zip › 多因素Cox分析-46-tiff/KIF20A.tif]

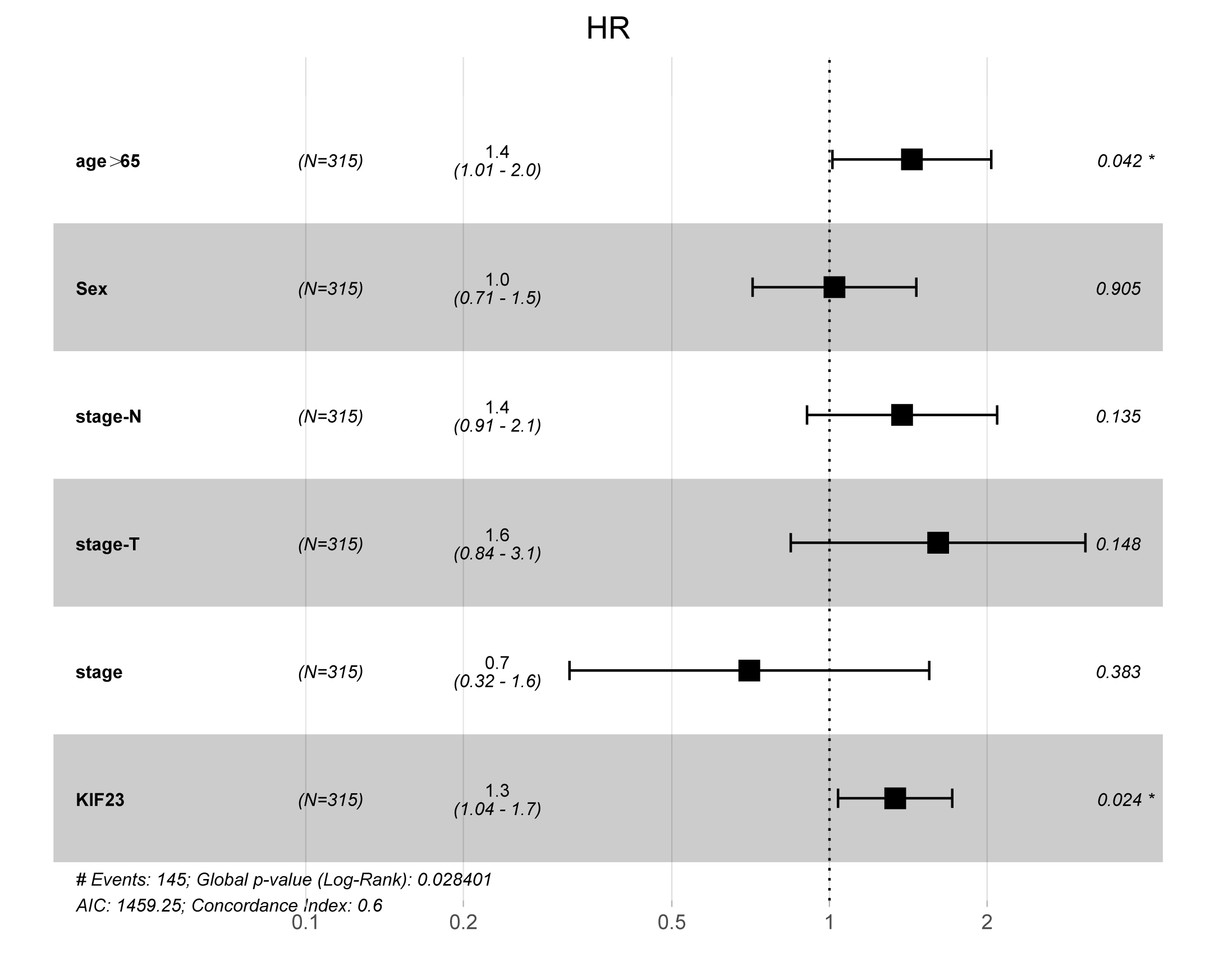

Supplement: S2 File — (ZIP) [file pone.0329622.s002.zip › 多因素Cox分析-46-tiff/KIF23.tif]

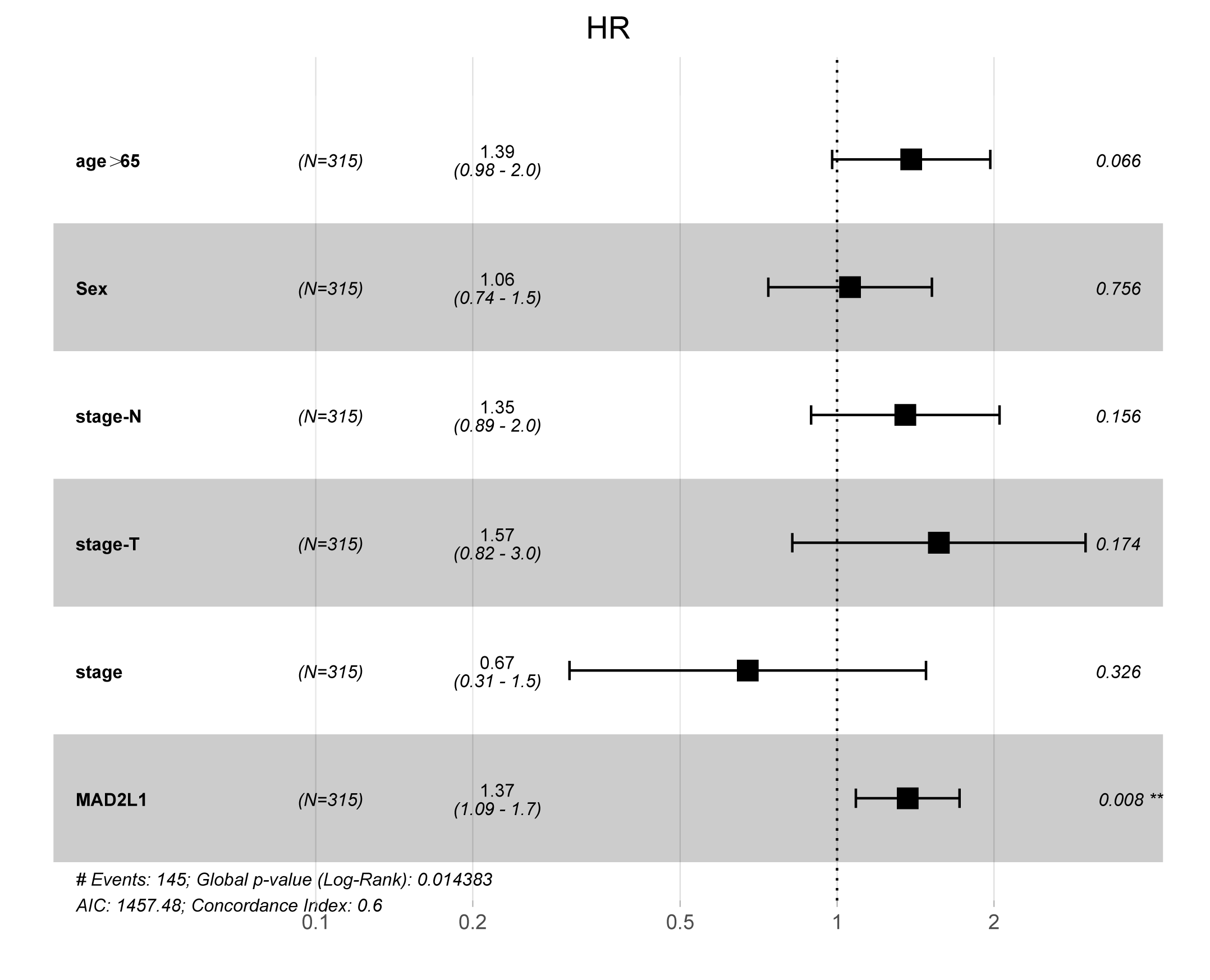

Supplement: S2 File — (ZIP) [file pone.0329622.s002.zip › 多因素Cox分析-46-tiff/MAD2L1.tif]

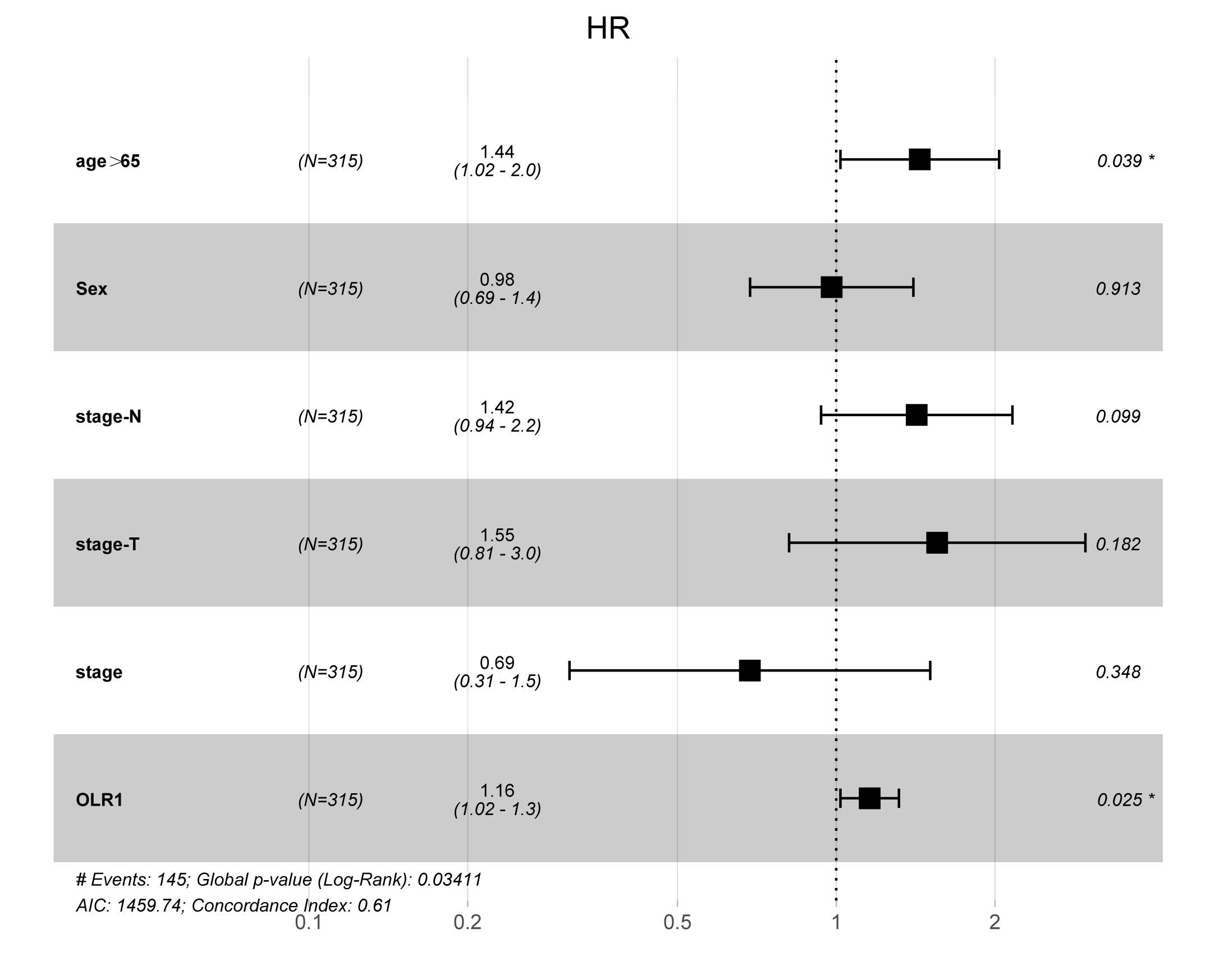

Supplement: S2 File — (ZIP) [file pone.0329622.s002.zip › 多因素Cox分析-46-tiff/OLR1.tif]

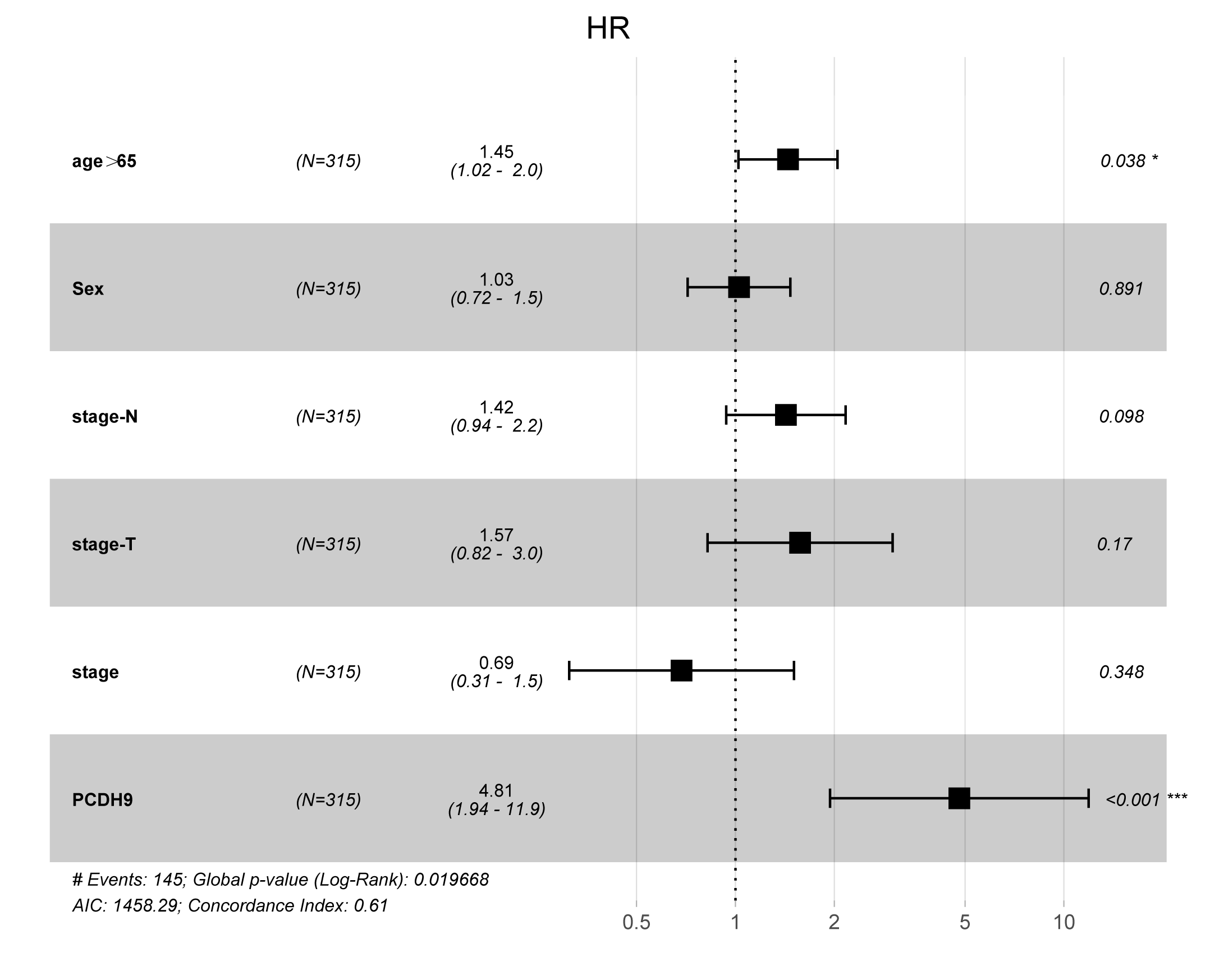

Supplement: S2 File — (ZIP) [file pone.0329622.s002.zip › 多因素Cox分析-46-tiff/PCDH9.tif]

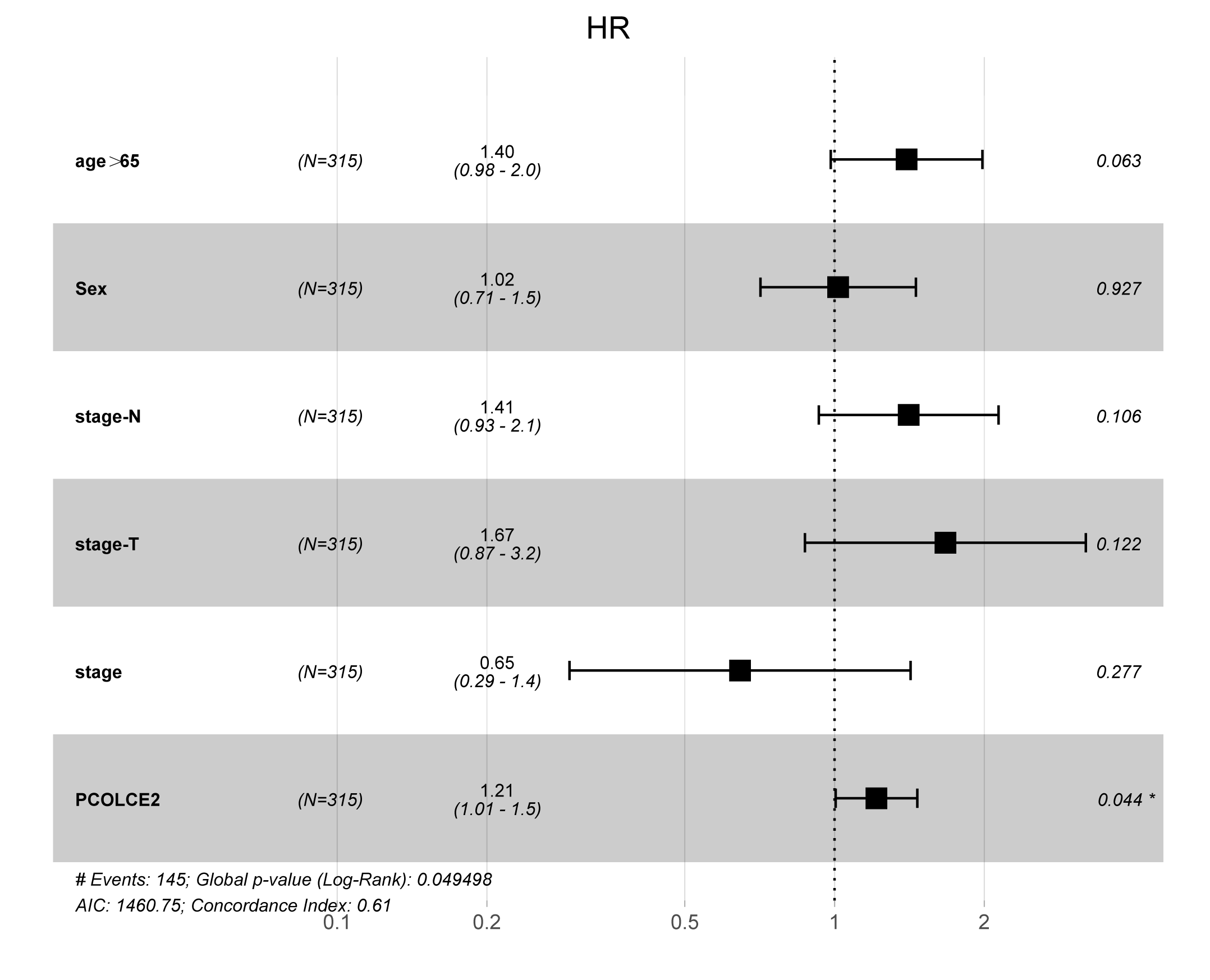

Supplement: S2 File — (ZIP) [file pone.0329622.s002.zip › 多因素Cox分析-46-tiff/PCOLCE2.tif]

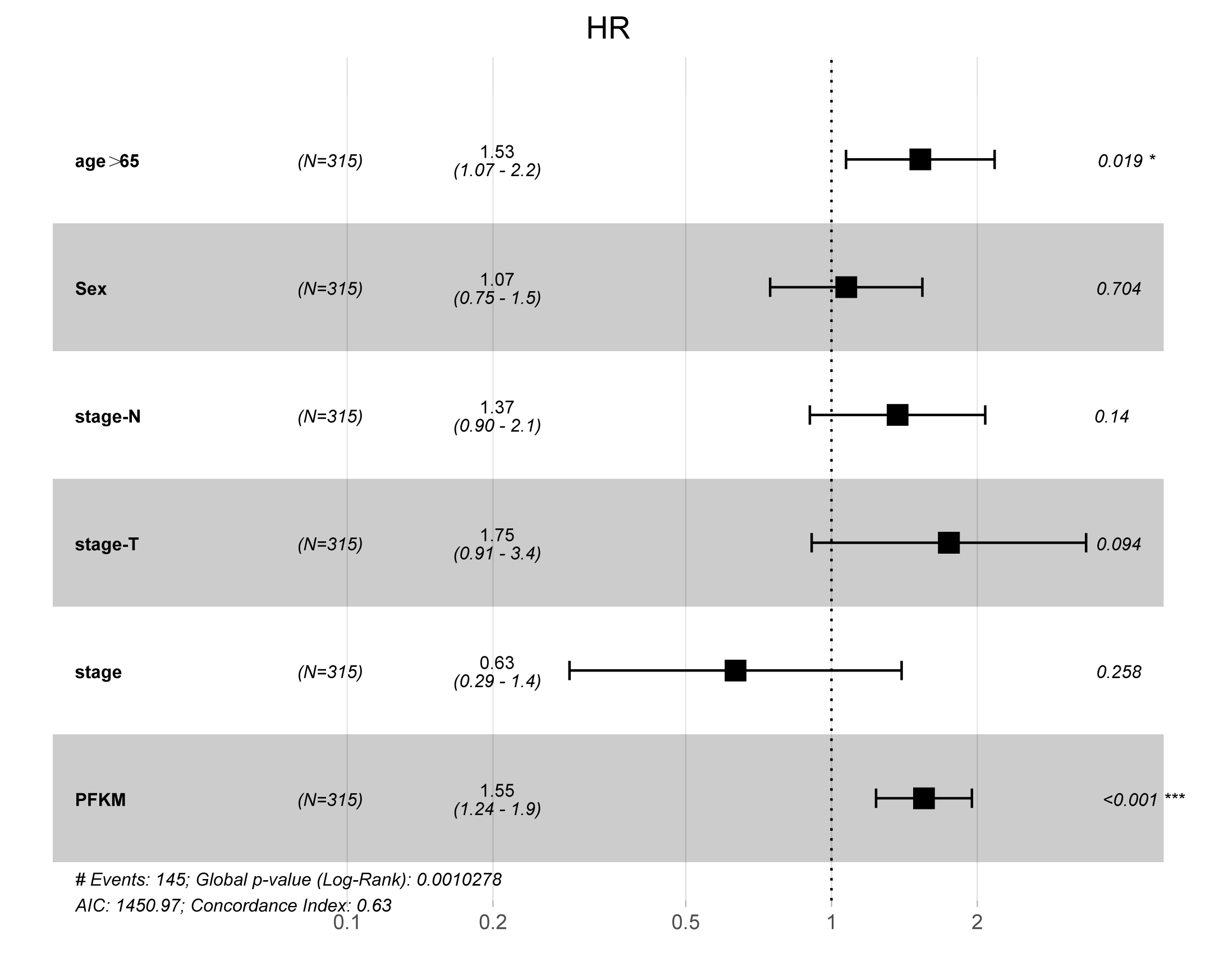

Supplement: S2 File — (ZIP) [file pone.0329622.s002.zip › 多因素Cox分析-46-tiff/PFKM.tif]

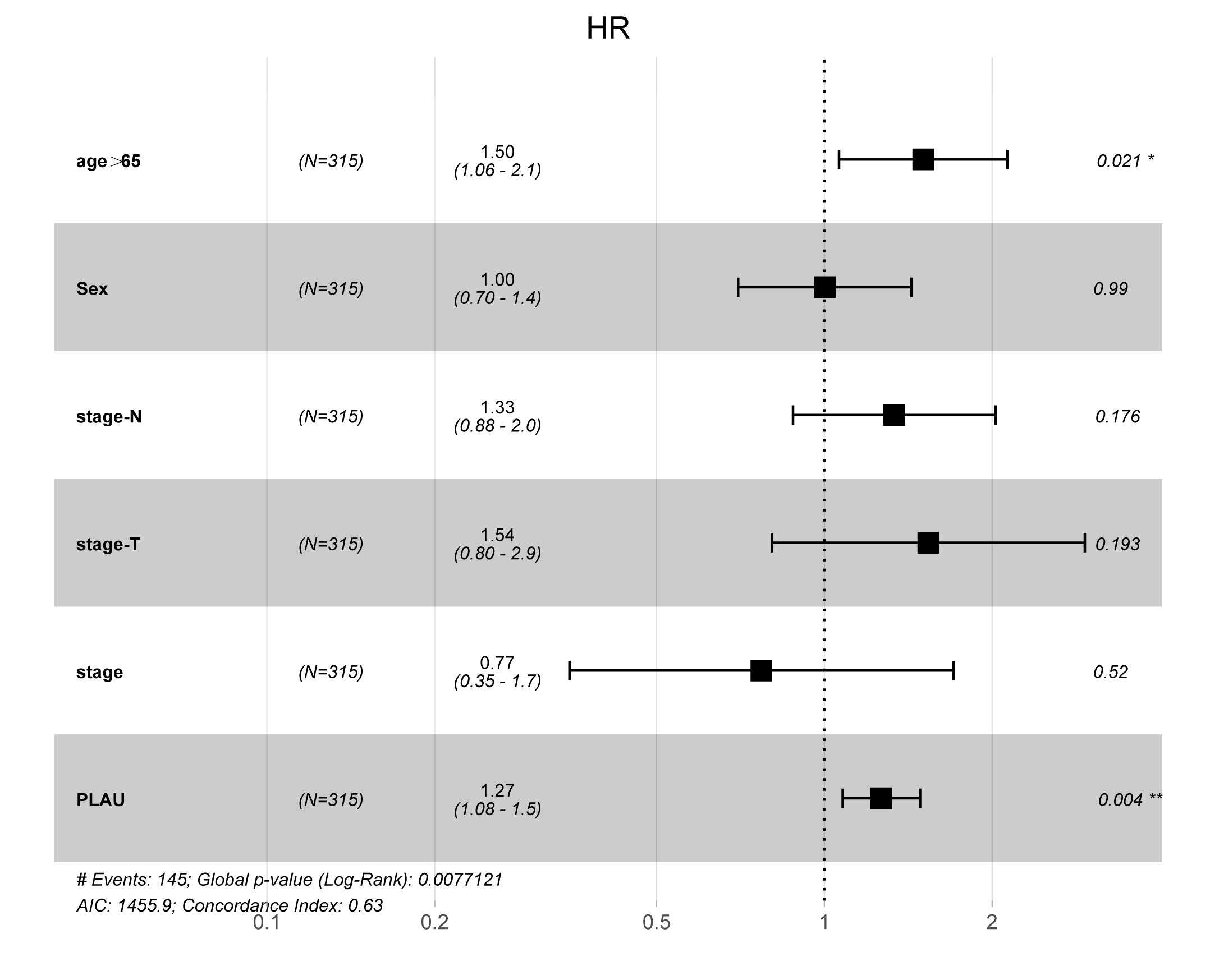

Supplement: S2 File — (ZIP) [file pone.0329622.s002.zip › 多因素Cox分析-46-tiff/PLAU.tif]

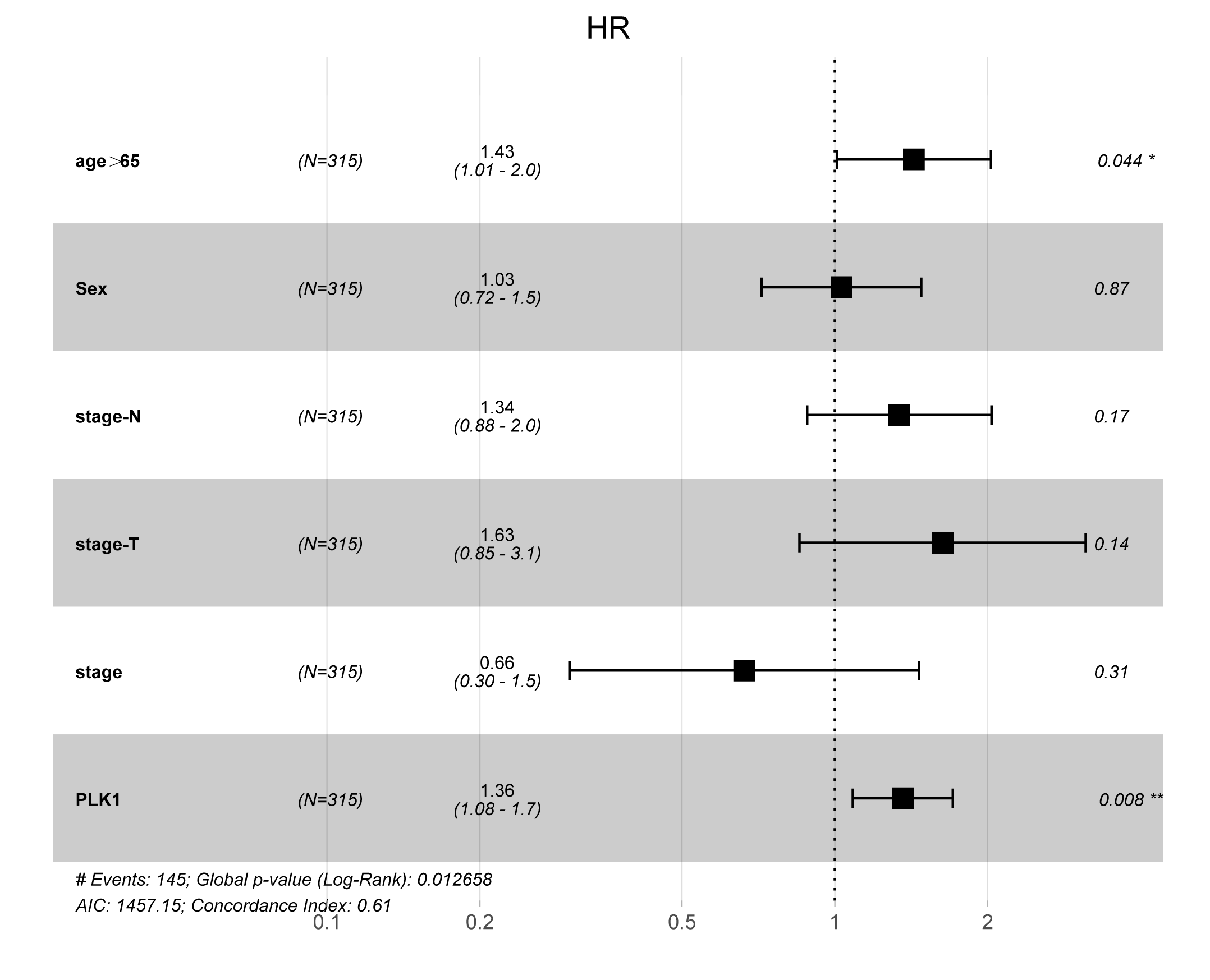

Supplement: S2 File — (ZIP) [file pone.0329622.s002.zip › 多因素Cox分析-46-tiff/PLK1.tif]

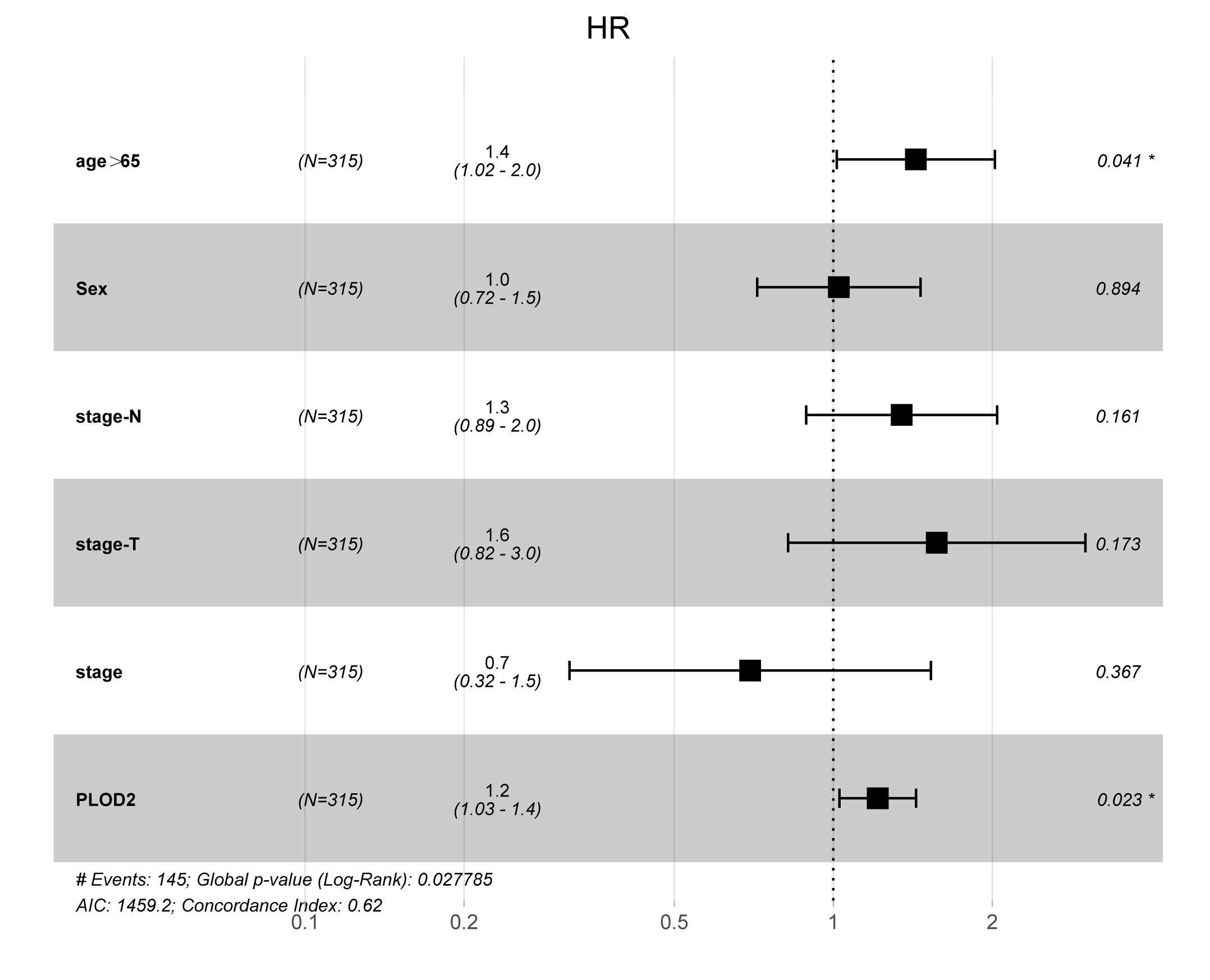

Supplement: S2 File — (ZIP) [file pone.0329622.s002.zip › 多因素Cox分析-46-tiff/PLOD2.tif]

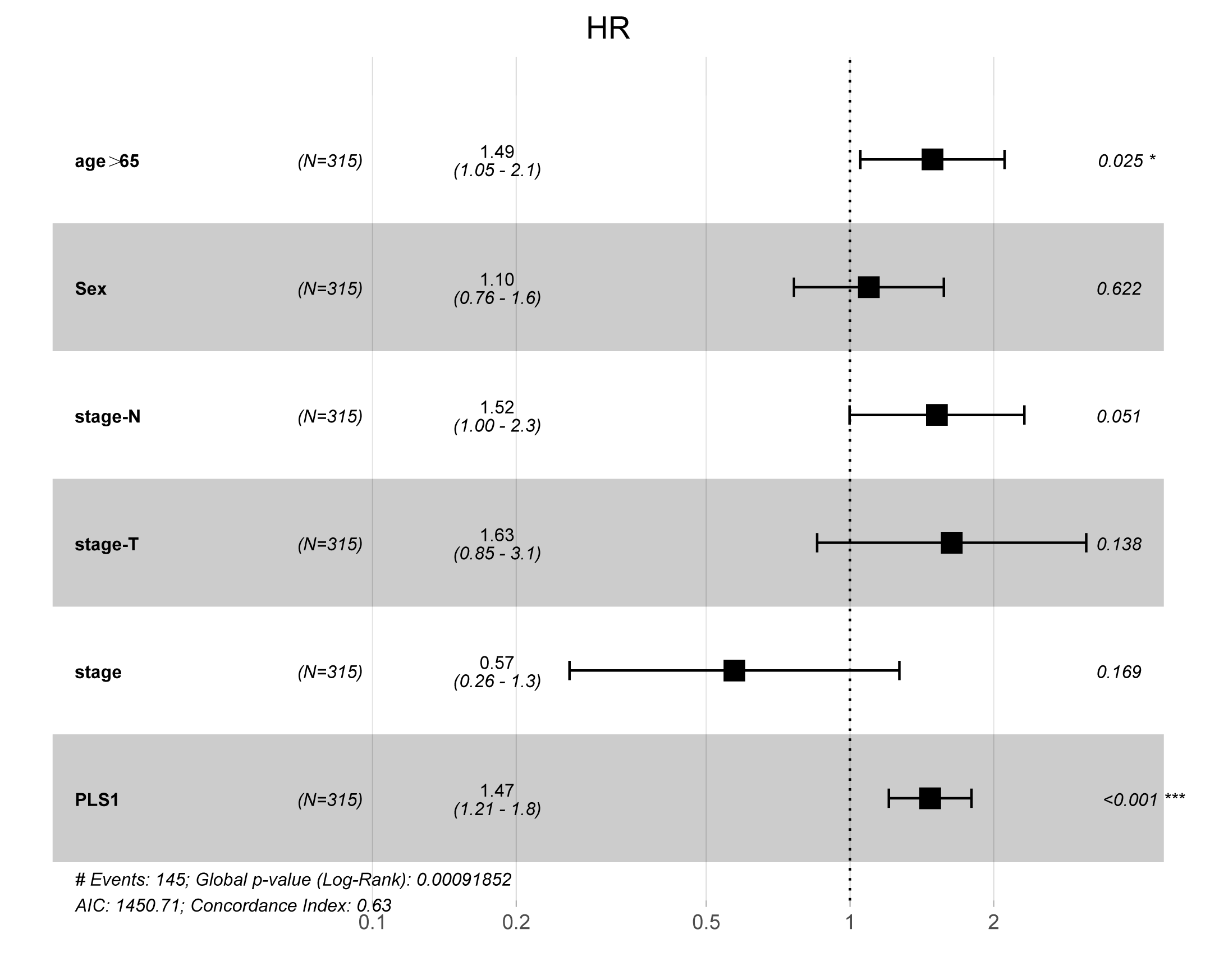

Supplement: S2 File — (ZIP) [file pone.0329622.s002.zip › 多因素Cox分析-46-tiff/PLS1.tif]

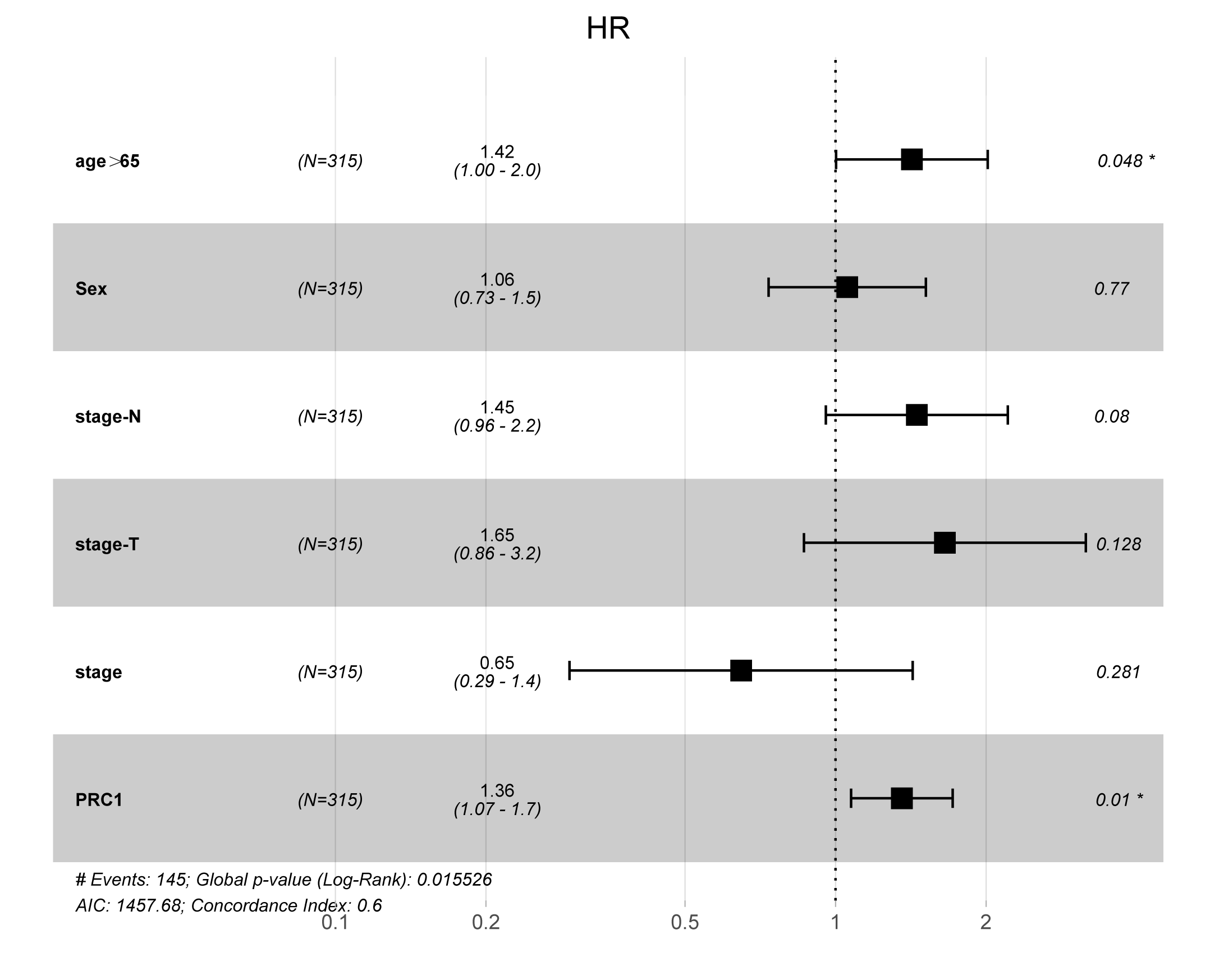

Supplement: S2 File — (ZIP) [file pone.0329622.s002.zip › 多因素Cox分析-46-tiff/PRC1.tif]

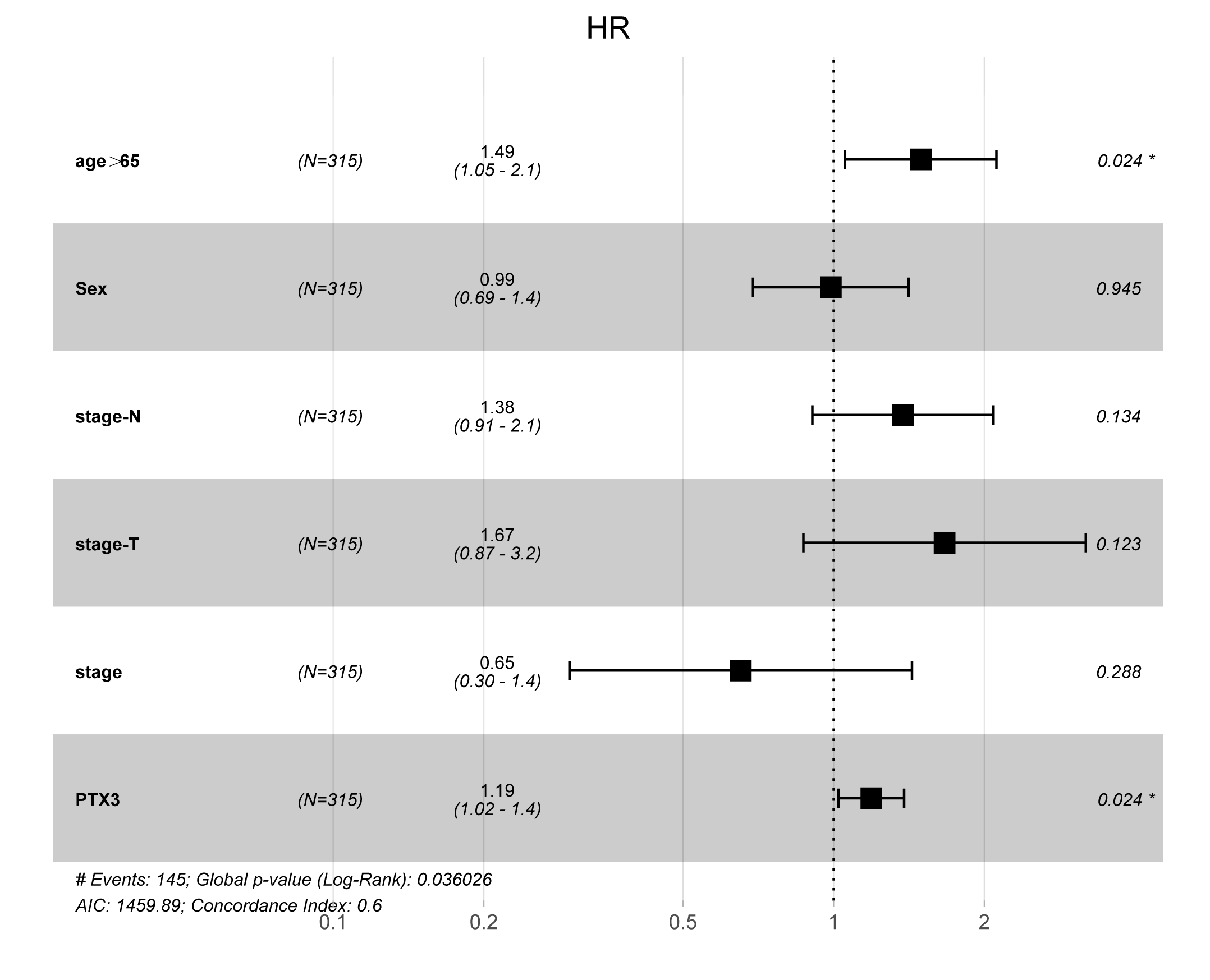

Supplement: S2 File — (ZIP) [file pone.0329622.s002.zip › 多因素Cox分析-46-tiff/PTX3.tif]

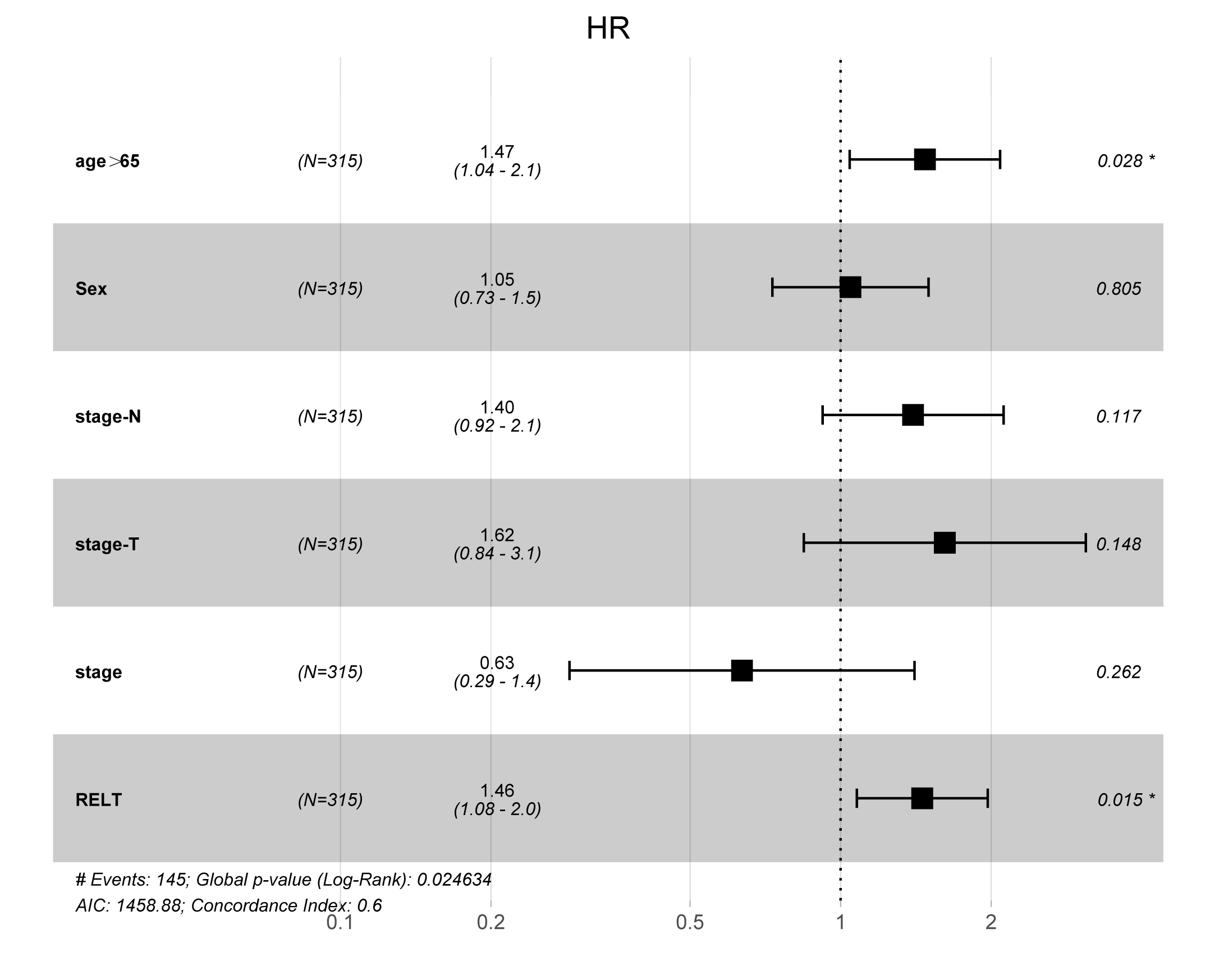

Supplement: S2 File — (ZIP) [file pone.0329622.s002.zip › 多因素Cox分析-46-tiff/RELT.tif]

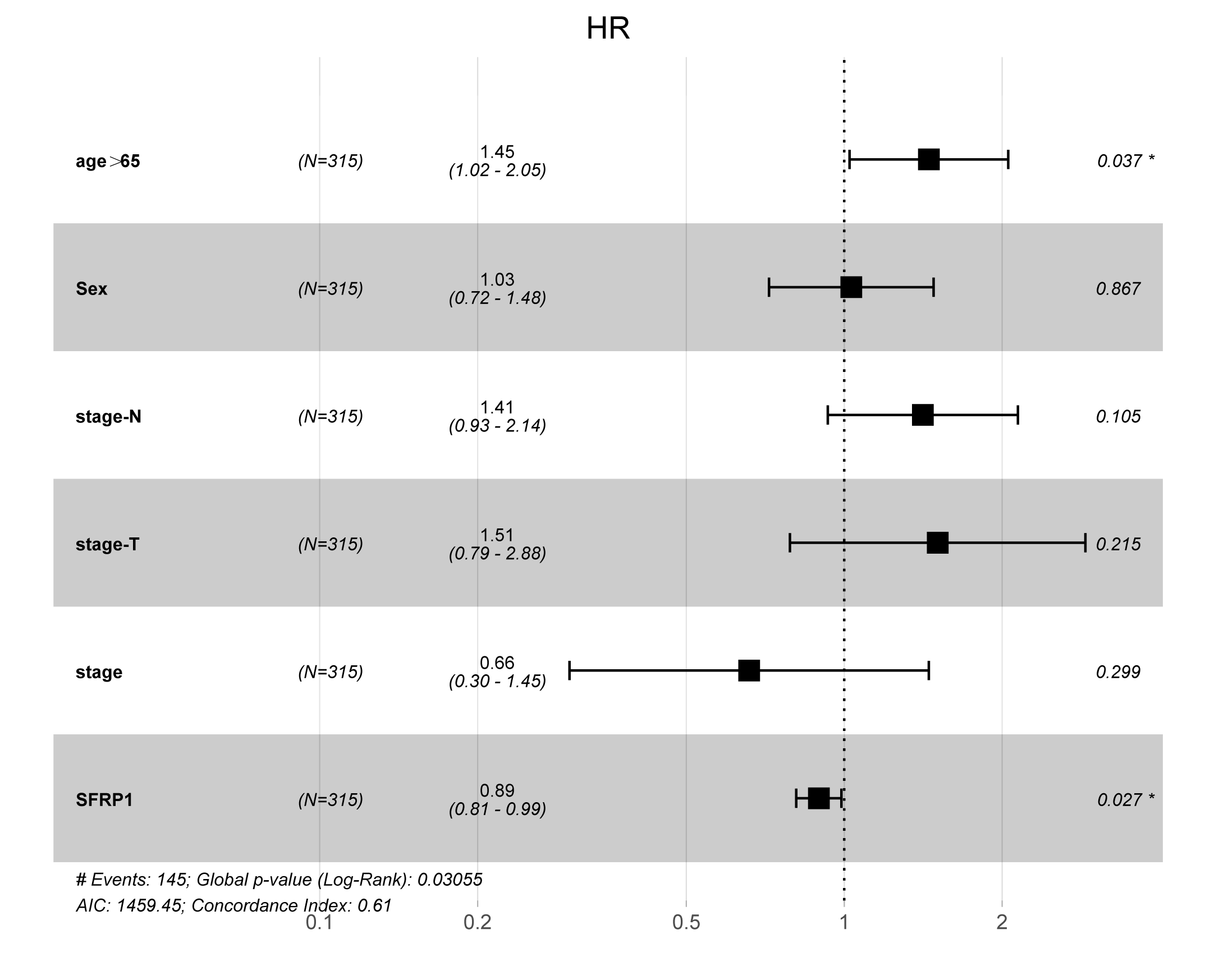

Supplement: S2 File — (ZIP) [file pone.0329622.s002.zip › 多因素Cox分析-46-tiff/SFRP1.tif]

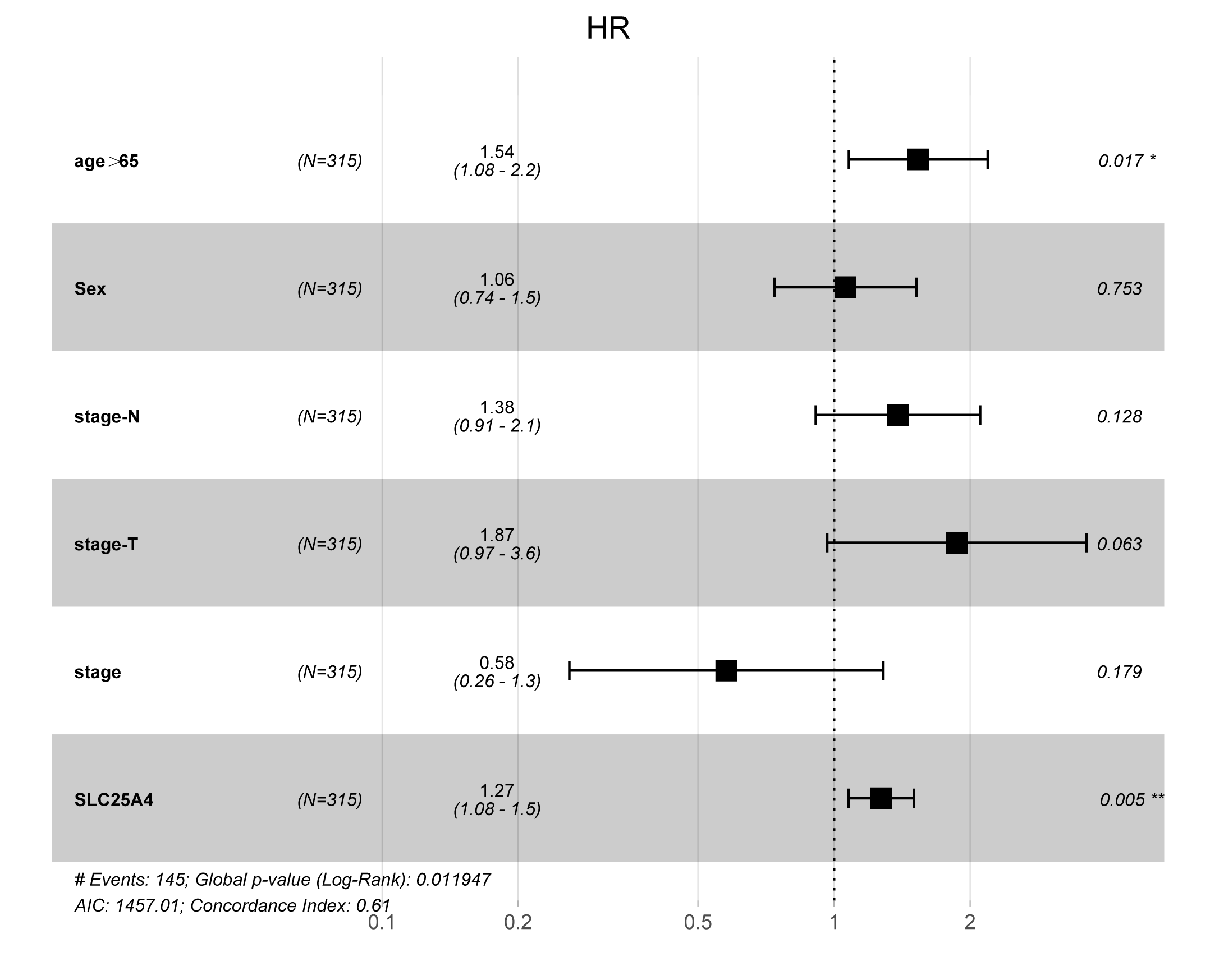

Supplement: S2 File — (ZIP) [file pone.0329622.s002.zip › 多因素Cox分析-46-tiff/SLC25A4.tif]

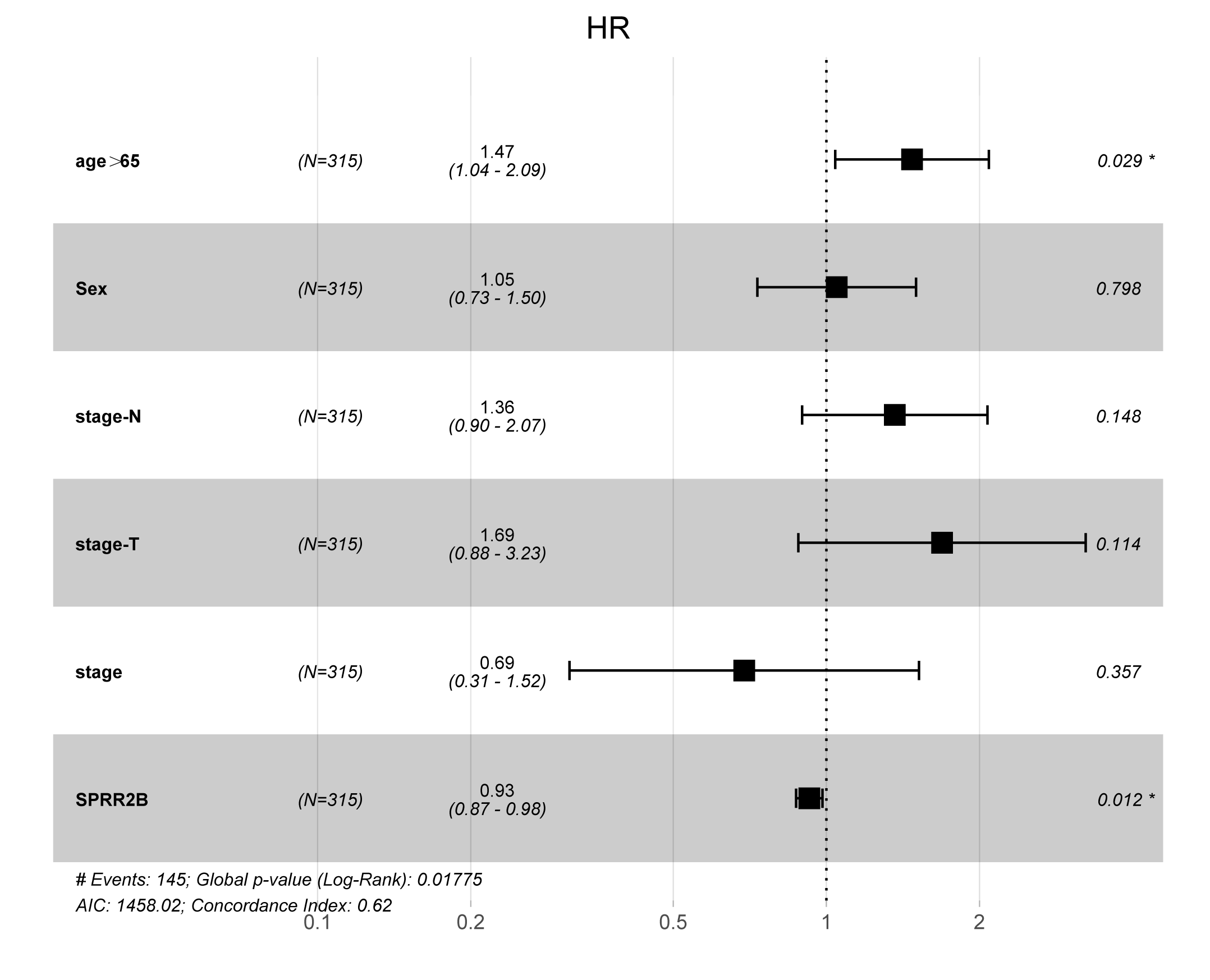

Supplement: S2 File — (ZIP) [file pone.0329622.s002.zip › 多因素Cox分析-46-tiff/SPRR2B.tif]

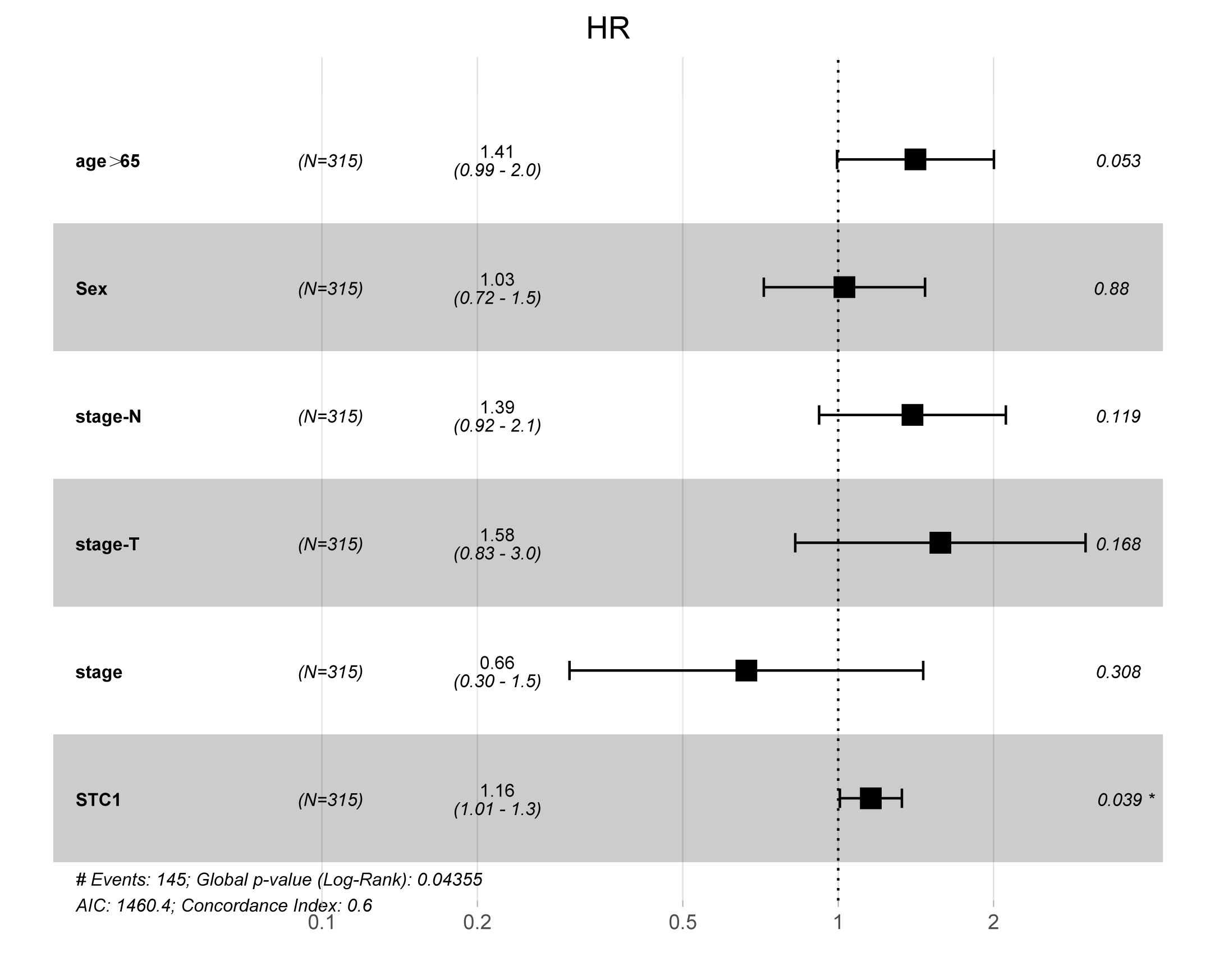

Supplement: S2 File — (ZIP) [file pone.0329622.s002.zip › 多因素Cox分析-46-tiff/STC1.tif]

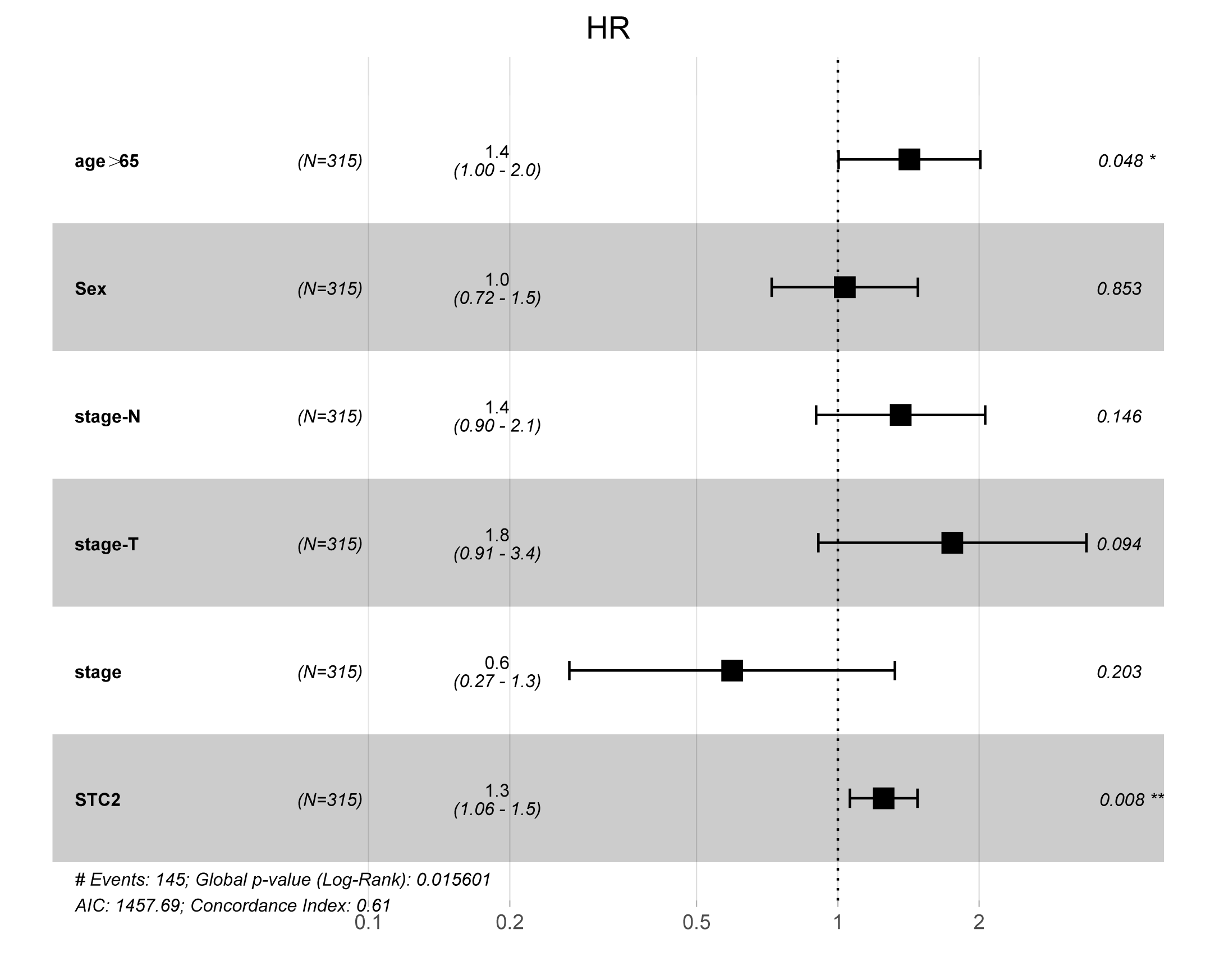

Supplement: S2 File — (ZIP) [file pone.0329622.s002.zip › 多因素Cox分析-46-tiff/STC2.tif]

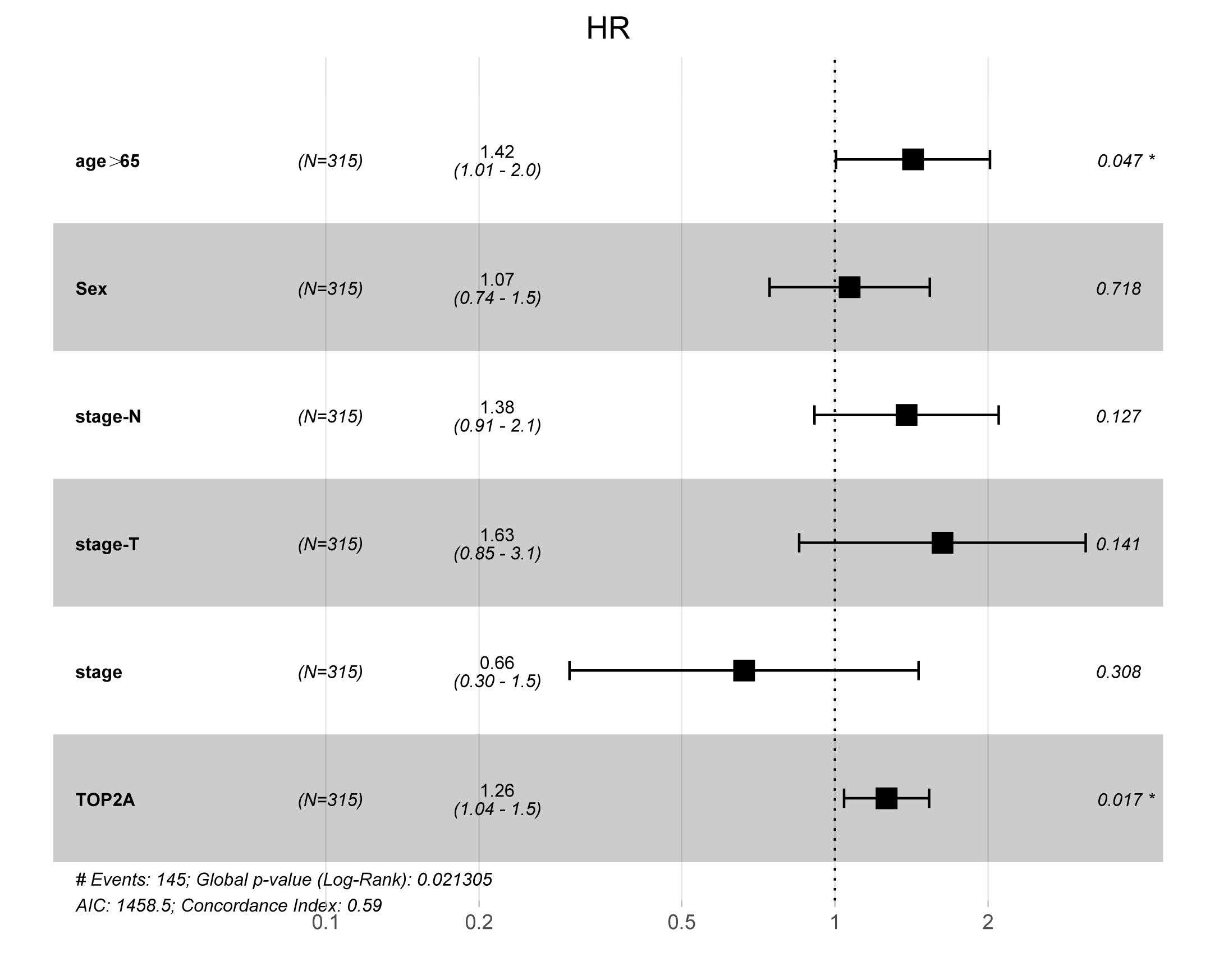

Supplement: S2 File — (ZIP) [file pone.0329622.s002.zip › 多因素Cox分析-46-tiff/TOP2A.tif]

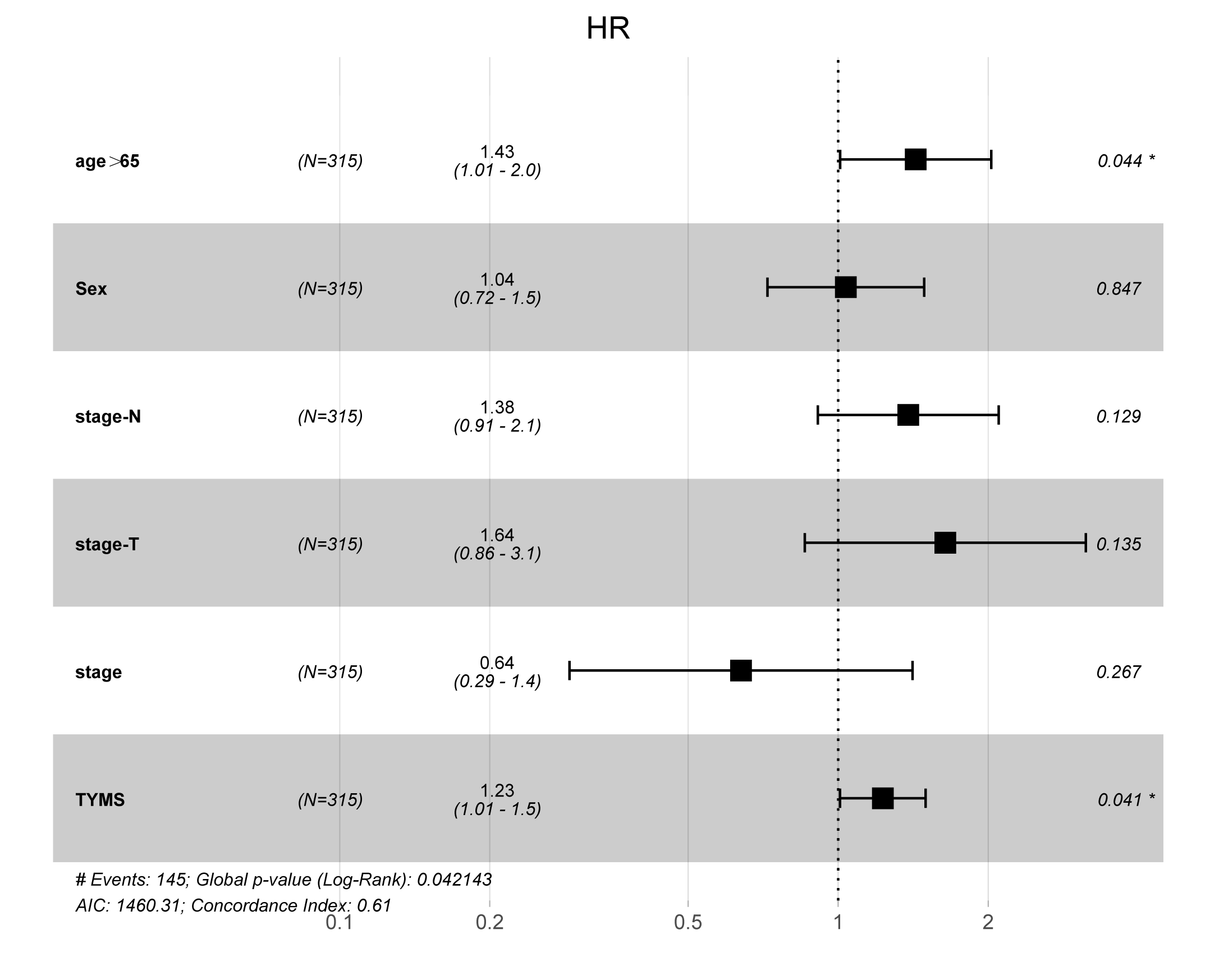

Supplement: S2 File — (ZIP) [file pone.0329622.s002.zip › 多因素Cox分析-46-tiff/TYMS.tif]

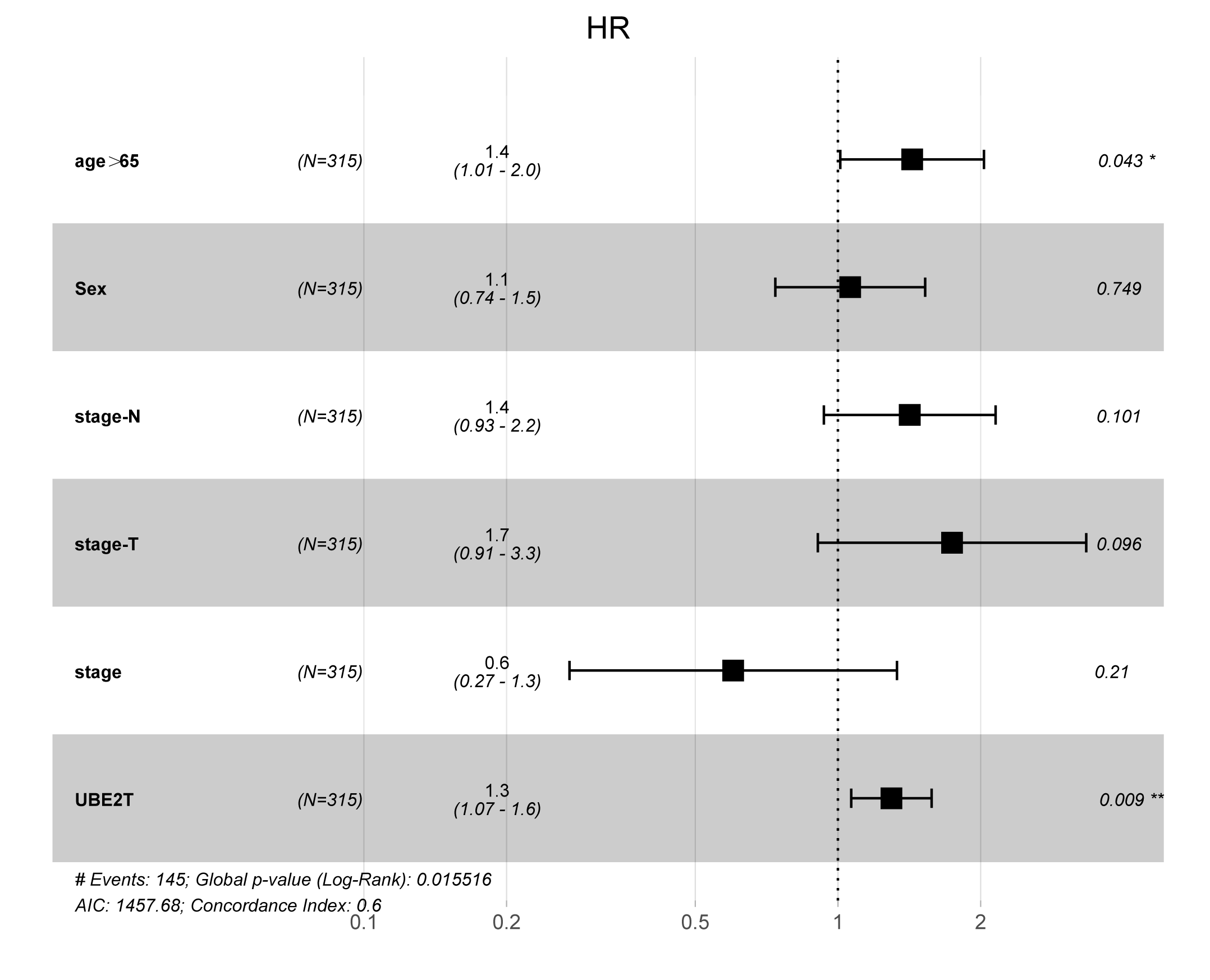

Supplement: S2 File — (ZIP) [file pone.0329622.s002.zip › 多因素Cox分析-46-tiff/UBE2T.tif]

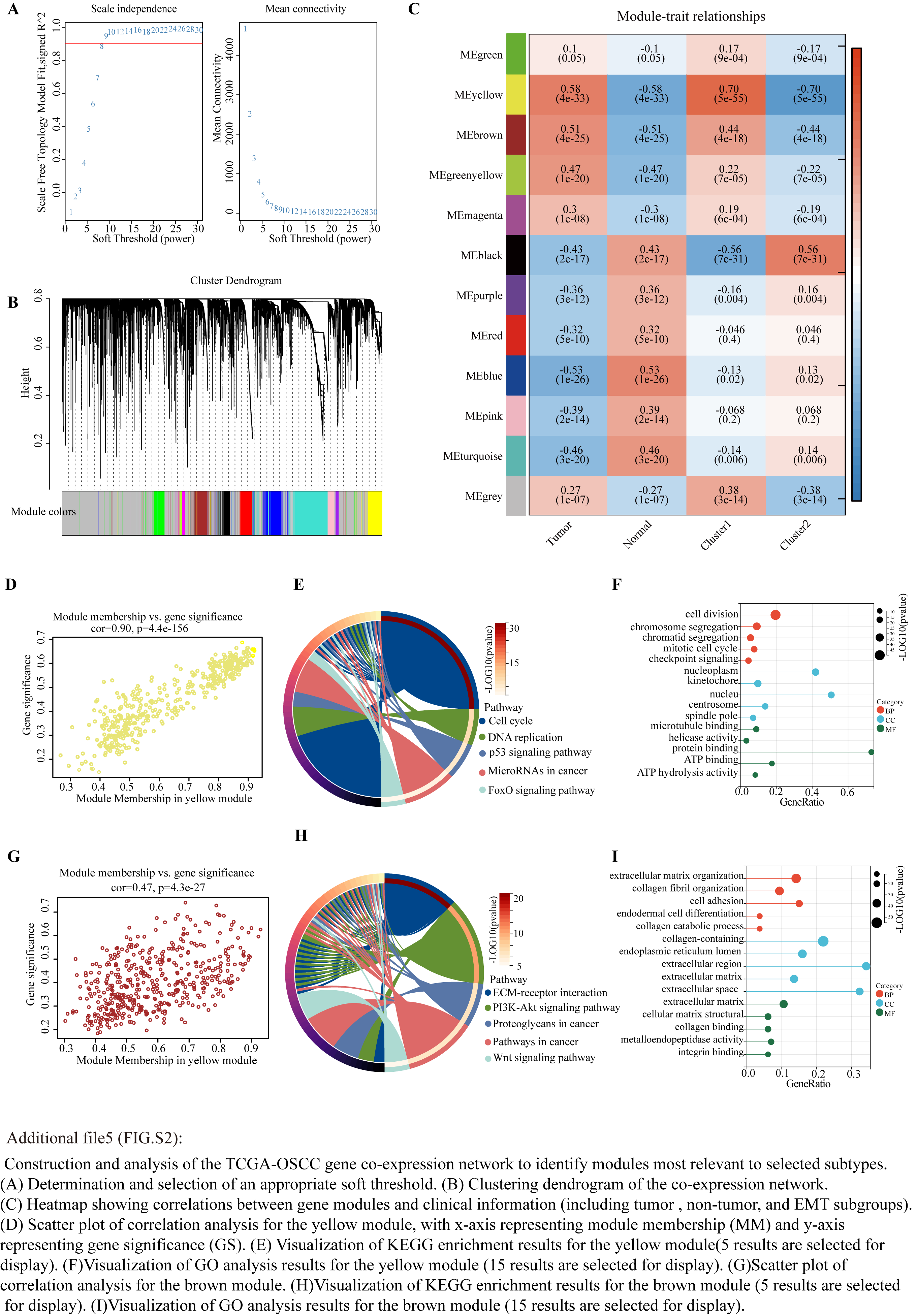

Supplement: S2 Fig — (TIF) [file pone.0329622.s011.tif]

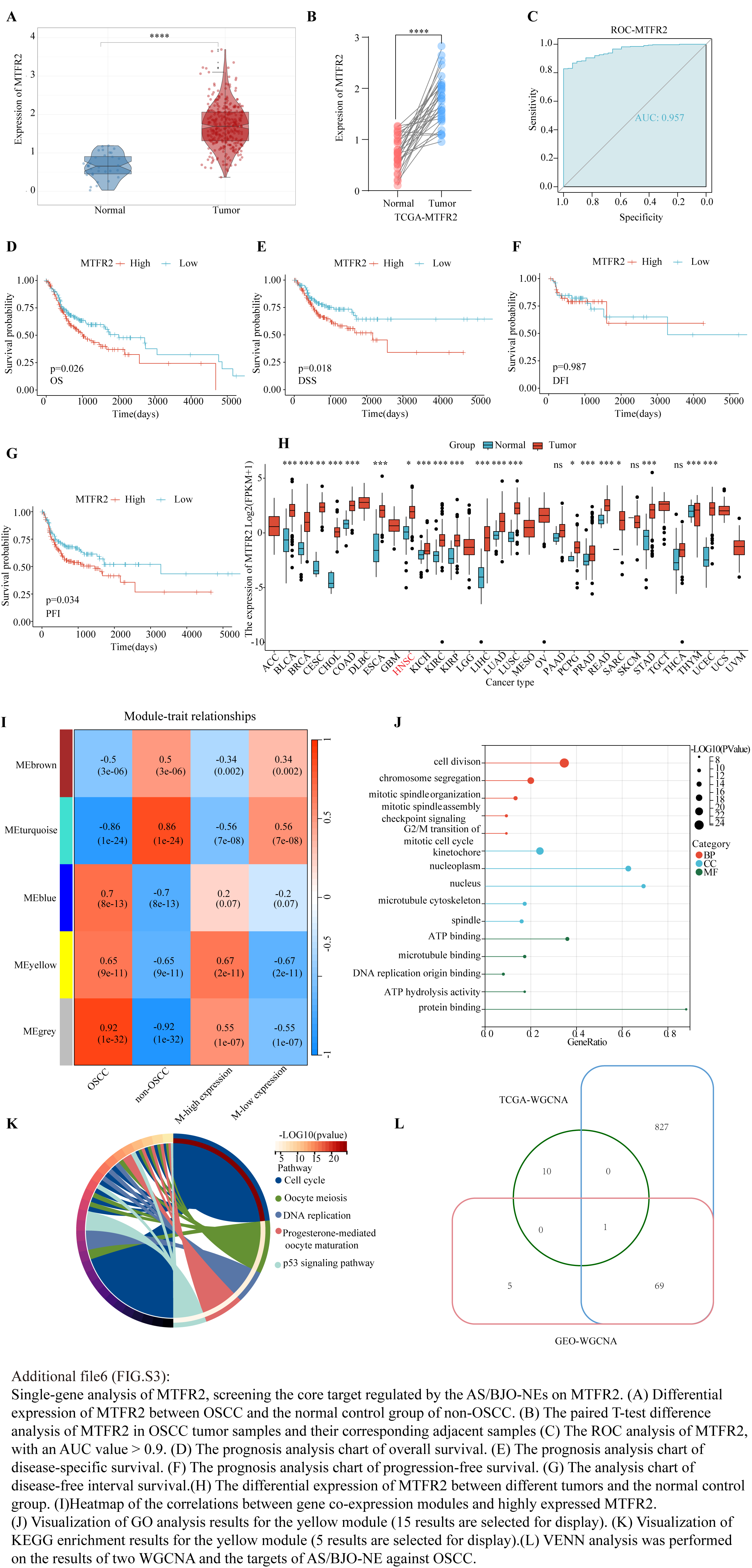

Supplement: S3 Fig — (TIF) [file pone.0329622.s012.tif]
