# Supplementary material for: Mechanism of Astragaloside-Brucea javanica oil nanoemulsion against oral squamous cell carcinoma through CDK1/MTFR2: Network pharmacology, bioinformatics, and experimental studies
Source: PLoS One. 2025 Aug 1;20(8):e0329622. doi: 10.1371/journal.pone.0329622 (PMC12316279; doi:10.1371/journal.pone.0329622)
Supplement: S1 Table — (PDF) [file pone.0329622.s003.pdf]

1、 CDK1（human）-shRNA lentivirus

Gene name：CDK1（GENE_ID：983 RefSeq ID：NM_001786.5）

Genetic origin： ☑shRNA clone

Species：Human

Name of the vector ：PLVX-shRNA2-Puro（#ZVE1006，Zolgene Biotechnology）

Component sequence：hU6-MCS-CMV-ZsGreen1-PGK-Puro

Cloning site：BamHI(GGATCC)-EcoRI(GAATTC)

Vector map:

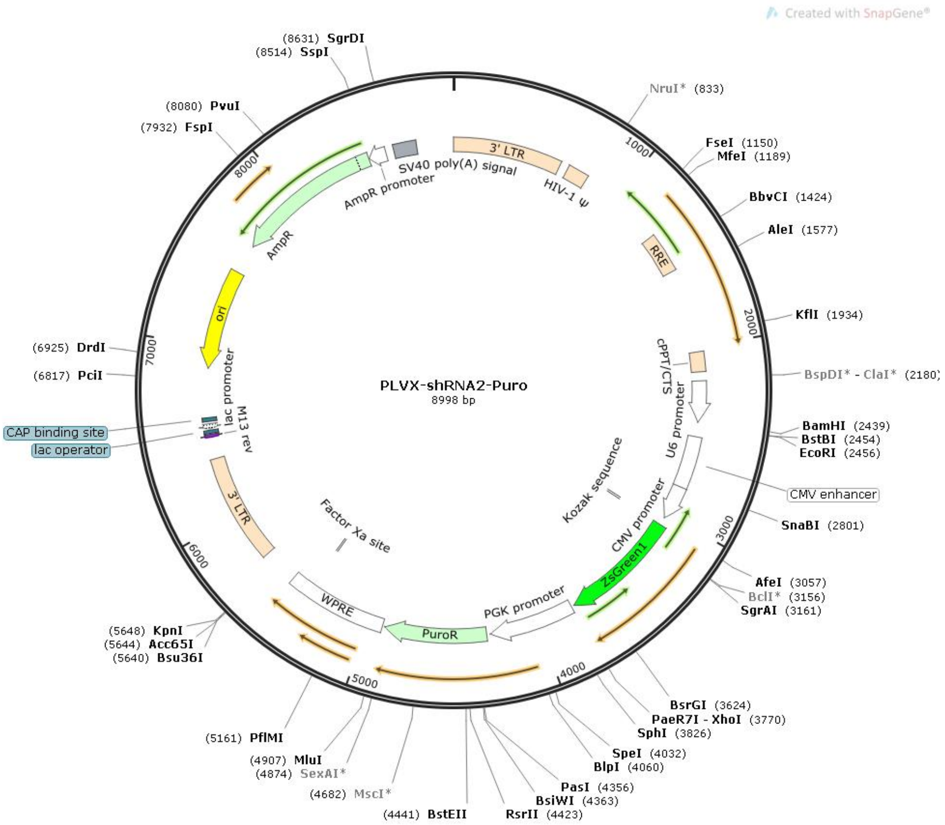

Sequence information:

zolgene	ShRNA Information	订单编号：	SO. 2024053016
		订单时间：	2024. 5. 15

NO.	Species	Name	5’	Sh	Loop	Sh	3’
1	Human（NM_001786.5）	CDK1-shRNA1-F	GATCC	GCTGTACTTCGTCTTCTAATT	TTCAAGAGA	AATTAGAAGACGAAGTACAGC	TTTTTG
			Sequence（5’ to 3’）	62	GATCCGCTGTACTTCGTCTTCTAATTTTCAAGAGAAATTAGAAGACGAAGTACAGCTTTTTG		
2		CDK1-shRNA1-R	AATTCAAAAA	GCTGTACTTCGTCTTCTAATT	TCTCTTGAA	AATTAGAAGACGAAGTACAGC	G
			Sequence（5’ to 3’）	62	AATTCAAAAAGCTGTACTTCGTCTTCTAATTTCTCTTGAAAATTAGAAGACGAAGTACAGCG		
3	Human（NM_001786.5）	CDK1-shRNA2-F	GATCC	GTGGAATCTTTACAGGACTAT	TTCAAGAGA	ATAGTCCTGTAAAGATTCCAC	TTTTTG
			Sequence（5’ to 3’）	62	GATCCGTGGAATCTTTACAGGACTATTTCAAGAGAATAGTCCTGTAAAGATTCCACTTTTTG		
4		CDK1-shRNA2-R	AATTCAAAAA	GTGGAATCTTTACAGGACTAT	TCTCTTGAA	ATAGTCCTGTAAAGATTCCAC	G
			Sequence（5’ to 3’）	62	AATTCAAAAAGTGGAATCTTTACAGGACTATTCTCTTGAAATAGTCCTGTAAAGATTCCACG		
5	Human（NM_001786.5）	CDK1-shRNA3-F	GATCC	GATTCAGAAATTGATCAACTC	TTCAAGAGA	GAGTTGATCAATTTCTGAATC	TTTTTG
			Sequence（5’ to 3’）	62	GATCCGATTCAGAAATTGATCAACTCTTCAAGAGAGAGTTGATCAATTTCTGAATCTTTTTG		
6		CDK1-shRNA3-R	AATTCAAAAA	GATTCAGAAATTGATCAACTC	TCTCTTGAA	GAGTTGATCAATTTCTGAATC	G
			Sequence（5’ to 3’）	62	AATTCAAAAAGATTCAGAAATTGATCAACTCTCTCTTGAAGAGTTGATCAATTTCTGAATCG		
	空载：	hU6-MCS-CMV-ZsGreen-PGK-Puro					

2、 CDK1（NM_001786.5）Overexpression lentivirus

Gene name：CDK1（GENE_ID：983 RefSeq ID：NM_001786.5）

Genetic origin： ☑Total gene synthesis

Species：Human

Name of the vector ：pCDH-CMV-MCS-EF1-copGFP-T2A-Puro（#ZVE1003，Zolgene Biotechnology）

Component sequence：CMV-MCS-EF1-copGFP-T2A-Puro

Cloning site：NheI（GCTAGC）-BamHI（GGATCC）

Vector map:

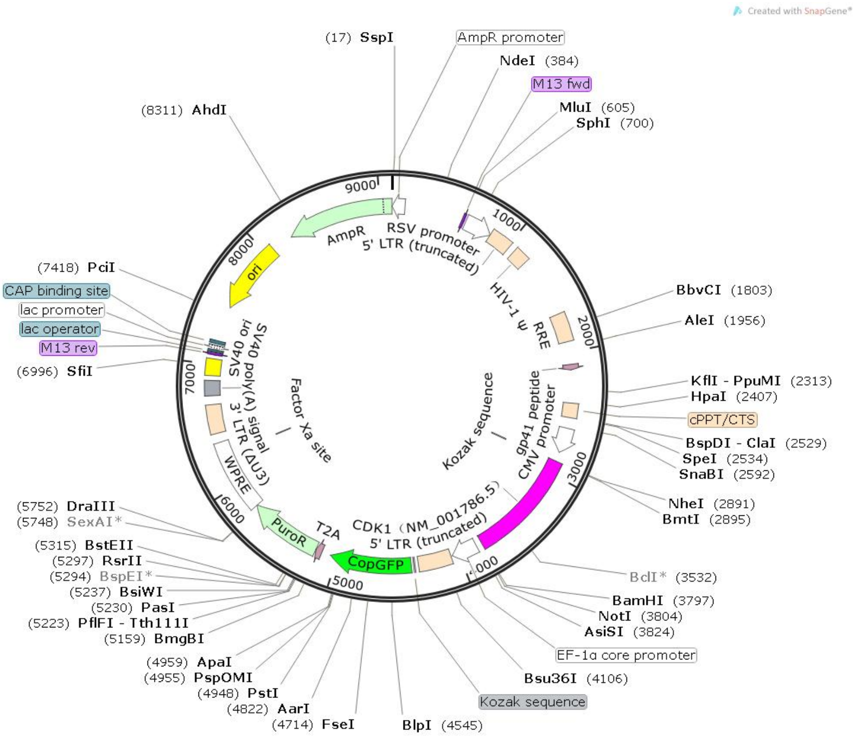

CDK1（NM_001786.5）重组质粒
9098 bp

Sequence information:

//GCTAGCGCCACCATGGAAGATTATACCAAAATAGAGAAAATTGGAGAAGGTACCTATGGAGTTGTGTATAAGGG
TAGACACAAAACTACAGGTCAAGTGGTAGCCATGAAAAAAATCAGACTAGAAAGTGAAGAGGAAGGGGTTCCTA
GTACTGCAATTCGGGAAATTTCTCTATTAAAGGAACTTCGTCATCCAAATATAGTCAGTCTTCAGGATGTGCTTATGC
AGGATTCCAGGTTATATCTCATCTTTGAGTTTCTTTCCATGGATCTGAAGAAATACTTGGATTCTATCCCTCCTGGTC
AGTACATGGATTCTTCACTTGTTAAGAGTTATTTATACCAAATCCTACAGGGGATTGTGTTTTGTCACTCTAGAAGA
GTTCTTCACAGAGACTTAAAACCTCAAAATCTCTTGATTGATGACAAAGGAACAATTAAACTGGCTGATTTTGGCC
TTGCCAGAGCTTTTGGAATACCTATCAGAGTATATACACATGAGGTAGTAACACTCTGGTACAGATCTCCAGAAGTA
TTGCTGGGGTCAGCTCGTTACTCAACTCCAGTTGACATTTGGAGTATAGGCACCATATTTGCTGAACTAGCAACTA
AGAAACCACTTTTCCATGGGGATTCAGAAATTGATCAACTCTTCAGGATTTTCAGAGCTTTGGGCACTCCCAATAA
TGAAGTGTGGCCAGAAGTGGAATCTTTACAGGACTATAAGAATACATTTCCCAAATGGAAACCAGGAAGCCTAGC
ATCCCATGTCAAAAACTTGGATGAAAATGGCTTGGATTTGCTCTCGAAAATGTTAATCTATGATCCAGCCAAACGAA
TTTCTGGCAAAATGGCACTGAATCATCCATATTTTAATGATTTGGACAATCAGATTAAGAAGATGTAGGGATCC//
