# Supplementary material for: Mechanism of Astragaloside-Brucea javanica oil nanoemulsion against oral squamous cell carcinoma through CDK1/MTFR2: Network pharmacology, bioinformatics, and experimental studies
Source: PLoS One. 2025 Aug 1;20(8):e0329622. doi: 10.1371/journal.pone.0329622 (PMC12316279; doi:10.1371/journal.pone.0329622)
Supplement: S7 Table — (PDF) [file pone.0329622.s009.pdf]

Table5. MMPBSA binding energy results.

Acting Force	Value(kj/mol)
Molecular mechanics	-149.654
Polar solvation free energy	137.407
Nonpolar solvation free energy	-19.389
Van der Waals force	-36.713
Electrostatic potential energy	-112.942
